# The gut microbiota of Labrador retriever puppies: a longitudinal cohort study

**DOI:** 10.1186/s42523-025-00464-2

**Published:** 2025-10-17

**Authors:** Charlotte S C Woolley, Adrian Muwonge, Barend M de C Bronsvoort, Jeffrey J Schoenebeck, Ian G Handel, Katie Chamberlain, Erica Rose, Dylan N Clements

**Affiliations:** 1 Centre for Epidemiology Versus Arthritis, Centre for Musculoskeletal Research, Division of Musculoskeletal and Dermatological Sciences, University of Manchester, Manchester, M13 9PL UK; 2 The Roslin institute and Royal (Dick) School of Veterinary Studies, University of Edinburgh, Edinburgh, EH25 9RG UK

**Keywords:** Epidemiology, Labrador retrievers, 16S rRNA, Longitudinal studies

## Abstract

**Background:**

Most research into the development of the canine gut microbiota has featured cross-sectional studies, and there has been limited exploratory research into how it is affected by external factors. We aimed to longitudinally characterise the gut microbiota and its development in Labrador Retriever puppies and identify whether alterations in the gut microbiota are associated with factors related to demography, lifestyle, antibiotic usage and gastrointestinal health.

**Results:**

76 Labrador Retriever puppies were recruited via Dogslife, a UK-based online cohort study. Faecal samples were collected at three to four, seven, and 12 months of age and analysed using 16 S rRNA gene sequencing alongside questionnaire data. Alpha and beta diversity were assessed using linear mixed effects models and permutational multivariate analysis, accounting for repeated measures. Differential abundance was evaluated using multivariable association with linear models. Associations were identified between puppies’ gut microbiota and age, sex, coat colour, household smoking status, dietary indiscretions (e.g. household waste, coprophagia), contact with other dogs and horses, recent oral/injected antibiotic use, and recent vomiting and diarrhoea. The greatest source of variation was individual identity, explaining approximately 25% of alpha diversity and 50% of beta diversity. Alpha diversity declined between three and 12 months, with age-related shifts in community composition and dispersion. Coprophagia was associated with increased alpha diversity and contributed to variation in community structure. Antibiotic use was associated with reduced alpha diversity, altered composition, and changes in taxa across *Firmicutes*, *Proteobacteria*, and *Tenericutes*. These effects were largely transient, with the largest shifts occurring within one week of treatment. Puppies with recent diarrhoea showed increased alpha diversity and differential abundance in several taxa within four weeks of the episode. *Helicobacter* was more frequently detected in samples from puppies with recent diarrhoea.

**Conclusions:**

This longitudinal study characterises the development of gut microbiota in Labrador Retriever puppies and identifies associations with demographic, environmental, and health-related factors. These findings underscore the value of longitudinal sampling in microbiome research, offer novel insights for owners and veterinarians, and lay a foundation for future studies investigating causal mechanisms and potential interventions.

**Supplementary Information:**

The online version contains supplementary material available at 10.1186/s42523-025-00464-2.

## Background

The canine gut microbiota, similar to the human gut microbiota, is vastly diverse and abundant in bacterial species [1–3]. In humans, large scale studies such as the Human Microbiome Project (HMP) and the integrated HMP have helped to characterise the human gut microbiota and established a baseline for ecological states within the healthy general adult population, demographically stratified subpopulations and specific disease states [4–7]. Despite these advances in the human field, most studies in dogs have focused on the characterisation of organisms rather than associations with intrinsic, demographic and environmental factors and their impact on canine health [8].

Bacteria play a key role in the development of their hosts’ gastrointestinal tract. In children, associations between the maturation of the immune system and the gut microbiota have been described [9]. The immune system of dogs becomes mature at approximately six months of age [10], but associations of age with the canine gut microbiota are not fully understood [11, 12]. Several cross-sectional studies have shown that the intestinal microbial communities of dogs are distinct at different ages [13–17]. The largest canine microbiome study to date is a cross-sectional study of the gut microbiota of over 900 dogs of mixed ages and breeds, recruited from the Dog Aging Project (DAP). A preprint of their results revealed a gradual shift in the composition of the gut microbiota as dogs aged [18]. In humans, the microbial differences between individuals are greater than the changes within individuals over time, which calls into question the validity of cross-sectional studies in this context [19, 20]. Small scale, yet highly controlled longitudinal studies, have demonstrated changes in the diversity and composition of the gut microbiota of dogs with age [21, 22]. Vilson and colleagues [23] reported one of the largest longitudinal canine gut microbiota studies: a cohort of 168 German Shepherd puppies and 30 bitches in Sweden. The composition, but not the diversity, of the faecal microbiota in puppies differed from seven weeks of age to 15 to 18 months of age. Other intrinsic factors that have been shown to affect the canine gut microbiota include neutering status [24], morphological size [25] and breed [23, 26].

The caretaking choices that dog owners make are likely to have a large effect on the composition and diversity of their dogs’ gut microbiota. Domesticated dogs experience major changes in their gut microbiota due to cohabitation with humans [17], and their microbiota are associated with those of the humans with which they cohabitate [27, 28]. Vilson and colleagues [23] demonstrated that German Shepherds who lived in large cities had greater diversity of their gut microbiota than did those in the countryside. In humans, research has associated rural living or the ‘farm-like effect’ with changes in the composition of the microbiome and a protective effect against allergies [29]. Another environmental factor of importance is diet, with studies having shown associations between the gut microbiota and macronutrient composition, fibre and protein content and diet type (commercial vs. home prepared) [18, 30].

Gastrointestinal symptoms a common source of morbidity among dogs [31, 32], and recent outbreaks of symptoms have occurred on a national scale [33], presenting prevention and management challenges to both owners and veterinarians. Several studies revealed associations between canine gastrointestinal diseases and the gut microbiota, including irritable bowel disease [34–36], chronic enteropathy [37, 38], acute [36, 39, 40] and chronic [41] diarrhoea and cancer [42]. However, many of these studies were cross-sectional, so it is not clear whether changes in the gut microbiota precede or succeed the onset of symptoms. Antibiotics are often used to treat gastrointestinal issues and have been shown to have significant long term effects on the human microbiome [43]. Similar effects have been shown in experimental studies in dogs [44, 45], but it is unclear what the effects are in the long-term.

It is vital that we improve our understanding of the development of the canine gut microbiota beyond classification and investigate its interactions with the host, associations with environment and ability to mediate disease [12]. This will allow informed advice to be given to dog owners and veterinarians and expose key areas for further research to improve canine health outcomes [46]. The aims of this study are to (1) longitudinally characterise the gut microbiota of Dogslife (a cohort of Kennel Club registered Labrador Retrievers) puppies via 16 S ribosomal RNA (rRNA) gene sequencing and (2) identify whether alterations in the gut microbiota are associated with factors related to demography, lifestyle, antibiotic usage and gastrointestinal health collected via specialised digestive health questionnaires (DHQs).

## Methods

### Recruitment

All participants in this study were recruited via the Dogslife project, a web-based cohort study of the health of pedigree UK Kennel Club registered Labrador Retrievers, which began in July 2010. Details of Dogslife’s design have been published previously [47, 48]. This study aimed to recruit registered puppies whose owners were willing to provide faecal samples and DHQs at three timepoints (referred to as “waves”), when they were three to four, seven and 12 months of age. These age groups were chosen for a variety of reasons; three to four months because the gut microbiota of puppies postweaning has been shown to be different [13], seven months as this is shortly after puppies have reached immunological maturity [10] but are still growing and 12 months because puppies are almost fully grown [49]. The cutoff age of 114 days was used to allow approximately one week for the outward postage of sample packs and sample collection before puppies turned four months (121 days) old.

Recruitment for newly registered Dogslife members to this study identified 363 puppies registered between the 1st of October 2017 and the 20th of June 2018 as possible participants. Among 363 puppies, 140 (38.57%) were older than the maximum target age (114 days old), three (0.83%) were not healthy (defined as those displaying symptoms of acute illness or diagnosed with chronic conditions), 12 (3.31%) had owners who had not consented to be contacted, and three (0.83%) were not included due to administrative error, which resulted in 205 (56.47%) puppies being targeted for recruitment. These owners were contacted by the Dogslife secretary by telephone and/or email to ask for their participation in the study.

### Sample collection, processing and storage

The sampling packs contained faecal sampling instructions, a DHQ, prepaid and addressed UN3773 compliant Safebox packaging (Safebox™, Royal Mail Group Ltd.) and a PERFORMAbiome GUT (a prototype of OMINgene GUT) sample collection kit (PERFORMAbiome GUT PB-200, DNA Genotek Inc., Ottawa, ON, Canada). If puppy owners returned their sample and it was evident that it had not been collected correctly, they were asked to repeat the sample. Puppy owners were reminded to return their samples up to three times before they were considered to have dropped out. Upon arrival, the samples were stored at room temperature. Approximately once every one to two weeks, samples were aliquoted into cryovials in a class II microbiological safety cabinet, ensuring high standards of hygiene to prevent contamination. The samples were stored at -80 °C in batches as recommended per the manufacturer’s instructions. Negative controls, including only the PERFORMAbiome GUT solution, were processed and stored periodically with the samples.

### Questionnaire data collection, data cleaning and variable selection

Data were collected from Dogslife questionnaires for recruited puppies from when they were registered to six months after each owner’s last sample collection was used in this study. Upon registration, all owners supplied demographic and geographic information about their puppies. Owners were reminded monthly via email to complete online lifestyle and health questionnaires until their puppies were aged one and quarterly when their puppies were aged over one.

The DHQ (see Additional File 1) was designed on the basis of a combination of yes/no, free text and five-point Likert scale questions using bias-minimising criteria according to published guidelines [50–52]. Themes within the questionnaire were based on factors hypothesised to affect either the gut microbiota or gastrointestinal health on the basis of published peer-reviewed scientific literature, including associations with age [13– 17, 21– 23, 53], contact with humans and other animals [17, 27, 28, 54, 55], rural living factors [23, 56], diet [30, 57, 58], supplementation [8, 59, 60], antibiotics [43–45] and gastrointestinal disease [34–37, 39–42]. DHQs were piloted on ten dog owners of the Small Animal Veterinary Hospital at the Royal (Dick) School of Veterinary Studies to identify any obvious areas of ambiguity or missing information, which allowed rapid, practical and cost-effective initial checks of their suitability. Further ongoing piloting of the DHQs was performed, as they were returned during wave one of the sample collections, to identify any areas of owner misunderstanding, and they were adjusted as necessary.

### Faecal DNA extraction and 16 S sequencing

Microbial DNA was extracted from the faecal samples and purified via QIAmp PowerFecal DNA Kits (QIAGEN, Benelux B.V., Hulsterweg 82, 5912 PL Venlo, The Netherlands) in randomly selected batches across all waves of sample collection, with at least one negative control processed during the storage stage in each batch. The manufacturer’s instructions were followed except for steps one to four, which were modified as advised by the PERFORMAbiome GUT microbial DNA purification protocol. Quantification and integrity checks were performed with a NanoDrop (Thermo Fisher; Massachusetts, USA) and 3% agarose gel (VWR Biotechnology; Pennsylvania, USA) electrophoresis.

To assess the variation in canine samples due to laboratory techniques, eight canine samples were subjected to repeated DNA extraction and polymerase chain reaction (PCR) amplification. Control samples from a mock microbial community [61] composed of 20 bacterial species (5% of each) were included in each of three 96 well plates containing sample aliquots. Therefore, a total of 242 samples (15 negative controls, 3 mock community controls and 224 canine samples, including 8 repeats) were submitted for library preparation and 16 S rRNA gene sequencing, which was performed by external technicians at the Integrated Microbiome Resource (Integrated Microbiome Resource, Dalhousie University, Halifax, Nova Scotia, Halifax, B3H 4R2, Canada). The IMR protocols are available on their website [62]. In brief, the V3 variable region of the 16 S rRNA gene was amplified via PCR using dual-index custom primers 341 F (5′- CCTACGGGAGGCAGCAG-3′) and 518R (5′- ATTACCGCGGCTGCTGG-3′), as described by Pollock and colleagues [63]. The PCR products were verified visually with gels, and the reactions were pooled in one plate, cleaned and normalised to make a library that was fluorometrically quantified prior to sequencing with 300 + 300 bp paired end V3 chemistry using Illumina MiSeq.

### Data analysis

The datasets generated during and/or analysed during the current study are available in the Edinburgh University Datashare repository at 10.7488/ds/3857 [64]. All the results are presented in a html output in Additional File 2, and the programmatic code for the data analysis is available in Additional File 3. The raw amplicon reads were analysed using QIIME2 (release 2019.10) [65] based on standard workflows [66]. Paired end reads were demultiplexed and checked for quality. Reads were filtered by trimming the first ten base pairs (bp), truncating forward reads at 148 bp and reverse reads at 158 bp and clustering into amplicon sequence variants (ASVs) using the DADA2 pipeline [67]. ASVs were aligned, uninformative sites were masked, and a phylogenetic tree was generated and rooted. The taxonomy of ASVs was classified against the SILVA SSU Ref NR dataset V.132 at 99% sequence similarity [68] using a pre-trained naïve Bayes classifier trimmed to contain only the V3 variable region.

All further analyses were performed in R, using base R (version 4.3.1), the *tidyverse* [69] package to manipulate the data, functions in *phyloseq* [70], *microbiome* [71] packages for rRNA 16 S data handling and *ggplot2* [69], *ggpubr* [72], *kableExtra* [73] and *gridExtra* [74] to visualise and present the data, unless otherwise stated. Recruitment and retention data for the study were plotted in a Sankey diagram using the *Riverplot* [75] package. The questionnaire data were summarised in tables, and differences in the data between waves were analysed by extracting 95% confidence intervals from *t*-tests. Lifetime observed prevalence rates and 95% confidence intervals per wave for antibiotic usage, severe diarrhoea and severe vomiting were estimated via the *Prevalence* [76] package.

The ASV table, taxonomic classifications, phylogenetic tree and sample metadata were converted to a phyloseq object (a data structure containing pre-filtered and clustered phylogenetic sequencing data) using the *qiime2R* [77] package. Mitochondria and chloroplast sequences were removed from the data. The *decontam* [78] package was used to identify contaminants, using both negative controls and estimated DNA concentrations obtained during laboratory work as the method of detection. The reads were normalised ranked subsampling via the *SRS* [79] package. After sample filtering and processing of the data, the phylogenetic tree was re-rooted via the *multi2di* function in the *ape* [80] package to resolve multichotomies.

Alpha diversity was analysed using total richness (number of observed operational taxonomic units), the Shannon index, the inverse Simpson index and Faith’s phylogenetic diversity (PD), which were calculated using the *alpha* function in the *microbiome* package and the *pd* function in the *picante* [81] package. Linear mixed effects models were fitted to each alpha diversity metric via the *lmerTest* [82] package, with all processed variables of interest (see Additional File 4) used as predictors. A random effect term was added for individual puppies to account for repeated sampling. The fits of the models were checked via diagnostic plots. Standard model performance metrics, including R^2^ and the intraclass correlation coefficient (ICC), were calculated using the *performance* [83] package. Variables with a *P-*value < 0.05 in any of the final models were plotted against each of the alpha diversity metrics compared.

Beta diversity was analysed using Bray-Curtis, Jaccard, unweighted UniFrac (UF) and weighted UF distances, which were calculated via the *distance* and *UniFrac* functions in the *phyloseq* package. Permutational multivariate analysis of variance (PERMANOVA) testing [84, 85] was performed using the *adonis2* function in the *vegan* [86] package with 999 permutations, as advised elsewhere [87, 88]. To estimate the sole effect of individual puppies on explaining differences in gut composition, puppy identity was first included in separate single PERMANOVA models as the only predictor. All processed variables of interest (see Additional File 4) were then added to a second PERMANOVA model with “blocking” strata so that random shuffling was restricted within levels of individual puppies to account for repeated sampling. Variables with a *P-*value < 0.05 in any PERMANOVA analysis were plotted in principal coordinates analysis (PCoA) plots via the *plot_ordination* function in *phyloseq* for each of the beta diversity distances compared.

For each categorical (non-continuous) variable, the homogeneity of multivariate dispersions was analysed by using the permutational multivariate analysis of variance (PERMDISP) [89] technique with the *betadisper* function in *vegan* and analysis of variance (ANOVA) to test for differences. *P*-values for PERMDISP were adjusted with the Benjamini and Hochberg (BH) correction [90] using the *p.adjust* function in base R to control for false discovery rates during multiple testing, referred to as “*Q-*values”.

For the taxonomic and differential abundance analyses, the data were normalised to ‘relative’ abundances rather than counts. The relative abundance and frequency of detection of taxa were summarised by phylum, order, class, family and genus. Differential abundance analysis using microbiome multivariable association with linear models (MaAsLin) was performed on data without rare taxa by filtering out those with a prevalence of less than 10%. All variables of interest that were identified as significant in the alpha or beta diversity analyses were included in the models via the *MaAsLin2* [91] package. The analysis method chosen was the Tweedie compound Poisson linear model based on high levels of zero inflation in the data. Further transformation and filtering were disabled. *MaAsLin2* automatically adjusts *P*-values using the BH method. ASVs were named with the numbers with which they occurred combined with the ‘best hit’ (i.e., lowest level of taxonomy assigned). The negative log of the *Q-*values multiplied by the sign of the coefficient for each variable and model was visualised using the *pheatmap* function in the *ComplexHeatmaps* [92] package.

## Results

### Recruitment and retention to study

Among the 205 puppies targeted for recruitment, 83 (40.49%) had owners who agreed to participate and were sent a sampling pack. During wave one of the sample collections, the owners of six puppies dropped out, and one puppy had an unusable sample. Therefore, 76 puppies were included in the final study, and their summary statistics are shown in Table 1.

**Table 1:** Summary statistics of puppies (*n* = 76) recruited for the Dogslife gut microbiota study

Variable		Number of puppies	Percentage of puppies
Sex	Female Male	41 35	53.95 46.05
Coat colour	Black Chocolate Fox red Yellow	27 11 16 22	35.53 14.47 21.05 28.95
UK region	England south England midlands & Wales England north Scotland	23 28 13 12	30.26 36.84 17.11 15.79
Area classification	Rural Suburban Urban	28 18 30	36.84 23.68 39.47
Household type	One or more adult and one or more children More than 1 Adult Retired (Single/Couple) Single Adult	23 33 11 9	30.26 43.42 14.47 11.84
Household smoking status	No Yes	72 4	94.74 5.260

**Fig. 1:**
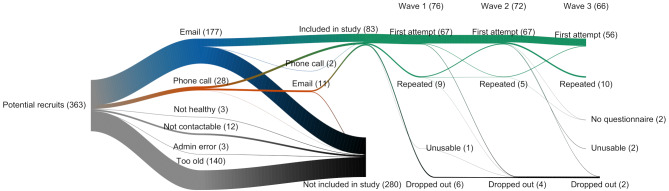
Recruitment, sampling success and retention of puppies recruited for the Dogslife gut microbiota study

Among these puppies, four dropped out during wave two, and 72 (94.74%) were retained. In wave three, two puppies dropped out, two had incomplete questionnaires, and two had unusable samples; thus, 66 (86.64%) were retained, as shown in Fig. 1.

**Fig. 2:**
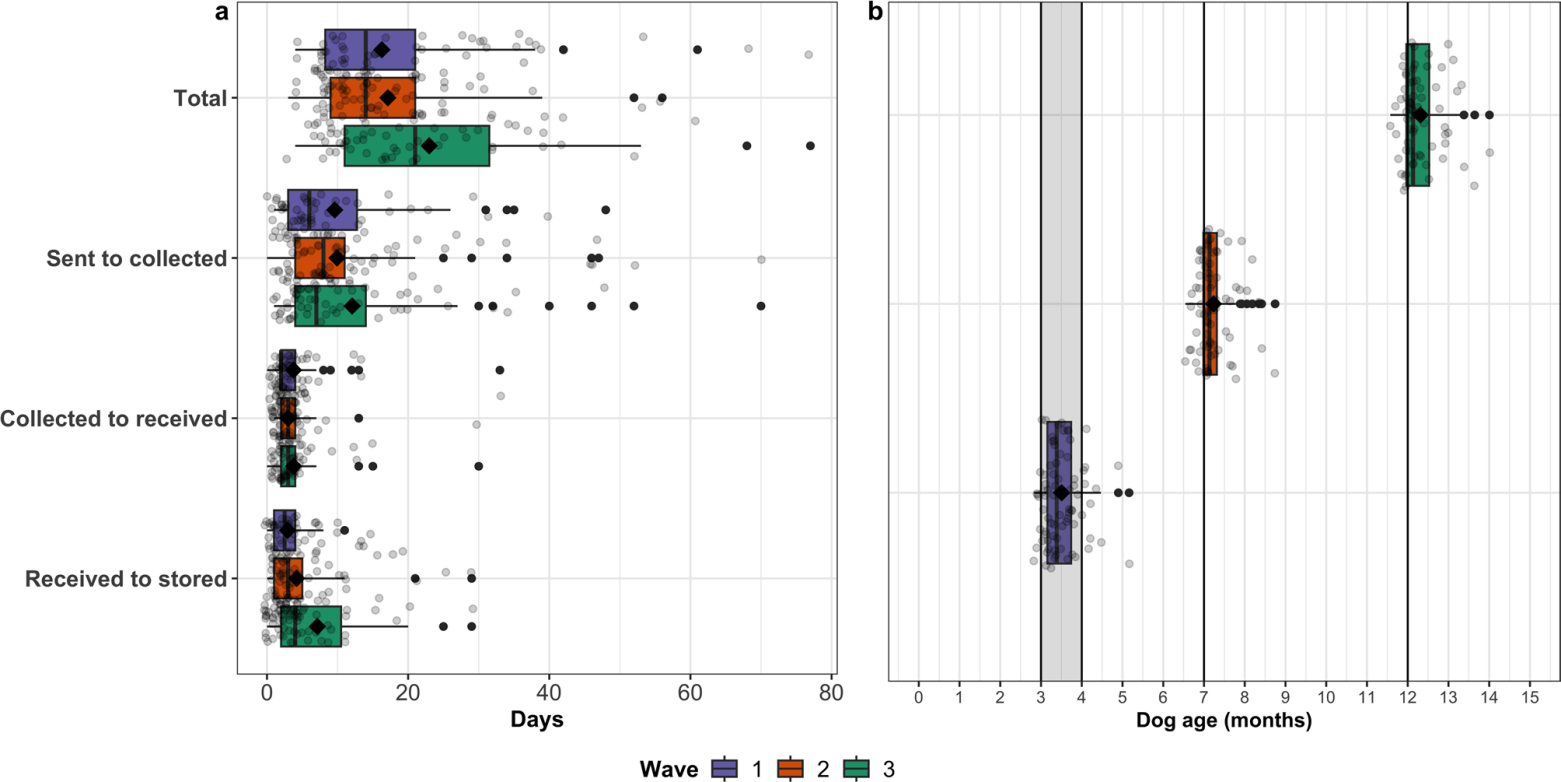
Recruitment, sampling success and retention of puppies recruited for the Dogslife gut microbiota study. (**a**) The time taken between sending sample packs and Dogslife owners collecting the samples, the samples being sent and received, and the samples being received and stored. (**b**) Age of Dogslife puppies at each target collection wave of the study: three to four months (wave one), seven months (wave two) and 12 months (wave three) of age

The mean age of the puppies reported by the owners during wave one of the sample collections was 3.512 (95% CI: 3.407–3.617) months, during wave two was 7.220 (95% CI: 7.121–7.319) months, and during wave three was 12.32 (95% CI: 12.19–12.44) months. The age of the puppies at each sample collection and the duration of each stage of sample collection and storage are shown in Fig. 2.

### Questionnaire results

The owners of the recruited puppies completed 469 Dogslife lifestyle and health questionnaires from when they were registered with Dogslife to six months after each owner’s last sample collection were used in this study. Among the 76 puppies recruited, 71 (93.42%) had at least one Dogslife lifestyle and health questionnaire. All but two DHQs were completed for each of the sample collections, which were in wave three. See Additional File 4 for details on how the questionnaire data were processed and cleaned. All the questionnaire results are reported in Sect. 10.3, Additional File 2. There were no significant differences between waves and the consumption of household or outdoor waste, coprophagia, stress scores or contact with dogs, cats, horses, farm animals or chickens.

Lifetime observed prevalence estimates for having ever received oral and/or injected antibiotics were 0.211 (95% CI: 0.119–0.302) in wave one, 0.310 (95% CI: 0.202–0.417) in wave two and 0.439 (95% CI: 0.320–0.559) in wave three. The lifetime observed prevalence estimates for having ever had a severe vomiting episode were 0.066 (95% CI: 0.010–0.122) in wave one, 0.239 (95% CI: 0.140–0.339) in wave two and 0.318 (95% CI: 0.206–0.431) in wave three. The lifetime observed prevalence estimates for having ever had a severe diarrhoea episode were 0.303 (95% CI: 0.199–0.406) in wave one, 0.493 (95% CI: 0.377–0.609) in wave two and 0.561 (95% CI: 0.441–0.680) in wave three.

### Sequencing results and analysis of laboratory effects

The 16 S rRNA data for 242 samples were successfully sequenced, demultiplexed, filtered and classified, returning 12,106,241 reads from 1814 taxa. The mean number of reads in canine faecal samples (*n* = 224) was 53,558 (95% CI: 51,950–55,166), that in mock community samples (*n* = 3) was 35,174 (95% CI: 19,492–50,855), and that in negative control samples (*n* = 15) was 252 (95% CI: 138–366). Canine faecal samples were compared with negative and mock community controls, and their alpha diversity and community characteristics were evidently different, as expected. See Sect. 7.1, Additional File 2 for further details.

There were differences in read count between repeated canine samples but large differences in taxonomy were not evident on average. The largest mean percentage change in a phylum between repeated samples was in Bacteroidetes, at 3.416% (95% CI: 0.039–6.793). See Sect. 7.2, Additional File 2 for further details. In all the negative controls (*n* = 15), a small amount of contamination was evident, but the mean number of reads for the negative control samples was 212 times less than the mean number of reads in the canine samples. Five genera (*Hymenobacter*,* Rhizobacter*,* Nostoc* PCC-73102, *Rubellimicrobium and Ralstonia*) were detected in the negative controls; these genera were not detected in the canine or mock community samples, which included 72 reads (1.904% of all negative control reads). There were 137 reads (3.623% of all negative control reads) that were not classified at the genus level. See Sect. 7.3, Additional File 2 for further details.

All 20 of the mock community species were detected at least at the genus level in all the mock community controls, demonstrating that the PCR process and taxonomic assignment were effective at detecting the expected organisms. Seven genera (*Prevotella* 9, *Fusobacterium*,* Faecalibacterium*,* Alloprevotella*,* Prevotellaceae* Ga6A1, *Megamonas*,* Sutterella*) were also detected that were not present in the original mock community sample. These genera were also detected in canine samples and thus appear to be the result of a small amount of cross-contamination. These totaled 296 reads (0.281% of mock community control reads). The over-represented phyla were *Firmicutes* (32.49% increase) and *Bacteroidetes* (32.35% increase), and the under-represented phyla were *Episilonbacteraeota* (69.16% decrease), *Deinococcus-Thermus* (34.72% decrease), *Actinobacteria* (32.70% decrease) and *Proteobacteria* (24.66% decrease) compared with the relative abundances expected in the mock community samples. See Sect. 7.4, Additional File 2 for further details.

There were no obvious differences in the number of reads or alpha or beta diversity between the DNA extraction batches or the PCR plates. See Sect. 8, Additional File 2 for further details.

### Data filtering, contaminant removal and normalisation

A total of 22 mitochondrial and chloroplast taxa and four contaminants were identified and removed from the data, reducing the number of taxa identified from 1814 to 1788. The reads were normalised to 29,454 reads, which led to the exclusion of all negative controls and one canine sample with a much lower value (4659 reads), which reduced the number of samples to 226 and the number of taxa to 1765. Finally, three mock community samples, two canine samples with missing DHQs and eight repeat canine samples (excluded at random) were removed from downstream analysis, which reduced the final number of samples to 213 and the number of taxa to 1731. These data were included in the alpha and beta diversity analyses. See Sects. 9 and 10, Additional File 2 for further details. After filtering taxa to those with a prevalence of 10% or more, 204 ASVs were included in the taxonomic and differential abundance analyses.

### Alpha diversity analyses

Linear mixed effects models were fitted to total richness, the Shannon index, the inverse Simpson index and Faith’s PD using all processed variables of interest (see Additional File 4) as predictors and a random effects term for individual puppies. The fits of the models were checked via diagnostic plots and performance metrics (see Sect. 11.1, Additional File 2). The R^2^ and the ICC were used to determine how much of the observed variance in alpha diversity was due to variations between or within individuals. The estimates for R^2^, ICCs, coefficients and *P*-values are shown in Table 2.

**Table 2:** Linear mixed effects models’ outputs for alpha diversity metrics in Labrador retriever puppy faecal samples

Variable	Richness		Faith’s PD		Shannon index		Inverse Simpson index	
	Coef	*P*	Coef	*P*	Coef	*P*	Coef	*P*
Wave of collection (Baseline = One)								
Two	-4.681	0.235	-0.179	0.310	0.027	0.648	1.559	0.113
Three	-10.61	*0.024	-0.447	*0.032	0.001	0.989	1.230	0.283
Sex (Baseline = Female)								
Male	-0.532	0.916	-0.110	0.617	0.034	0.591	0.940	0.396
Coat colour (Baseline = Black)								
Chocolate	13.23	0.126	0.267	0.476	0.038	0.727	0.030	0.987
Fox red	-6.845	0.345	-0.343	0.279	-0.128	0.160	-1.098	0.485
Yellow	-2.311	0.710	-0.025	0.926	-0.046	0.551	-0.224	0.868
Household type (Baseline = Family (One or more adult and one or more children))								
More than one Adult	-2.586	0.670	-0.120	0.650	0.025	0.743	-0.121	0.926
Retired (Single/Couple)	-0.898	0.914	-0.112	0.757	0.028	0.789	0.677	0.710
Single Adult	-12.94	0.126	-0.495	0.177	-0.046	0.657	0.065	0.971
Smoking status (Baseline = No)								
Yes	-11.07	0.339	-0.244	0.627	-0.007	0.962	0.155	0.951
UK region (Baseline = England South)								
Eng Midlands & Wales	0.951	0.880	0.201	0.465	-0.090	0.256	-1.291	0.348
Eng North	-2.466	0.759	-0.006	0.986	-0.128	0.206	-2.068	0.240
Scotland	-9.610	0.236	-0.547	0.123	-0.146	0.154	-1.908	0.281
Area classification (Baseline = Rural)								
Suburban	-3.300	0.666	-0.344	0.304	0.128	0.191	1.241	0.459
Urban	-5.528	0.407	-0.242	0.405	0.035	0.681	0.601	0.681
Frequency of waste consumption								
Household	-2.682	0.122	-0.060	0.438	-0.035	0.161	-0.452	0.277
Outdoors	-0.265	0.862	-0.009	0.890	0.007	0.731	-0.027	0.941
Coprophagia	7.592	*<0.001	0.421	*<0.001	0.080	*0.003	0.909	*0.039
Stress score	0.758	0.853	-0.069	0.703	-0.012	0.832	0.155	0.873
Frequency of contact with other animals								
Dogs	4.754	0.076	0.312	*0.009	0.036	0.308	0.566	0.351
Cats	1.606	0.279	0.003	0.962	0.015	0.439	0.519	0.117
Horses	-5.265	*0.041	-0.277	*0.015	-0.039	0.257	-0.356	0.545
Farm animals	-0.452	0.835	-0.022	0.819	-0.010	0.732	-0.547	0.282
Chickens	1.620	0.504	0.171	0.111	0.016	0.632	-0.070	0.899
Time since last receiving oral and/or injected antibiotics (Baseline = Never)								
Under 1 week	-34.18	*<0.001	-0.950	*0.018	-0.667	*<0.001	-5.346	*0.015
1 to 4 weeks	-3.787	0.708	0.246	0.585	-0.171	0.254	-1.748	0.479
4 to 8 weeks	-11.10	0.238	-0.116	0.780	-0.256	0.066	-3.196	0.162
8 to 16 weeks	-2.528	0.791	0.059	0.890	-0.182	0.197	-1.822	0.433
Over 16 weeks	-1.043	0.884	0.127	0.686	-0.039	0.692	-0.626	0.706
Time since last severe vomiting episode (Baseline = Never)								
Within 4 weeks	7.426	0.407	0.123	0.757	0.255	0.052	1.306	0.544
4 to 8 weeks	4.181	0.677	-0.086	0.847	-0.006	0.966	-0.820	0.737
8 to 16 weeks	9.000	0.558	0.142	0.835	0.275	0.231	2.083	0.580
Over 16 weeks	-6.876	0.368	0.254	0.453	-0.040	0.713	-0.986	0.584
Time since last severe diarrhoea episode (Baseline = Never)								
Within 4 weeks	-1.346	0.837	0.580	*0.046	-0.061	0.514	-0.649	0.677
4 to 8 weeks	12.37	0.206	0.182	0.675	0.243	0.088	2.893	0.220
8 to 16 weeks	3.566	0.630	-0.116	0.723	-0.083	0.440	-1.587	0.372
Over 16 weeks	9.598	0.130	0.231	0.407	0.090	0.297	0.212	0.884
Random effects								
Conditional R^2^	0.521		0.531		0.365		0.320	
Marginal R^2^	0.293		0.331		0.263		0.154	
ICC	0.322		0.299		0.138		0.196	

PD: Phylogenetic diversity. Coef: Coefficients. *P-values lower than 0.05

The conditional R^2^ (the variance attributed to both fixed and random effects) explained between 32.0% and 53.1% of the total models’ variances, depending on the diversity metric used. The marginal R^2^ (the variance attributed to the fixed effects alone) explained between 15.4% and 33.1% of the total models’ variances, depending on the diversity metric used. The ICC was between 13.8% and 32.2% depending on the diversity metric used, indicating that a large proportion of the explained variance is attributed to the interindividual effects of puppies. The variables significantly different in alpha diversity in at least one of the distance measures were wave of collection, coprophagia score, dog contact score, horse contact score, time since last receiving oral and/or injected antibiotics and time since last severe diarrhoea episode. See Sect. 11.1, Additional File 2 for boxplots of alpha diversity metrics by these variables.

**Fig. 3:**
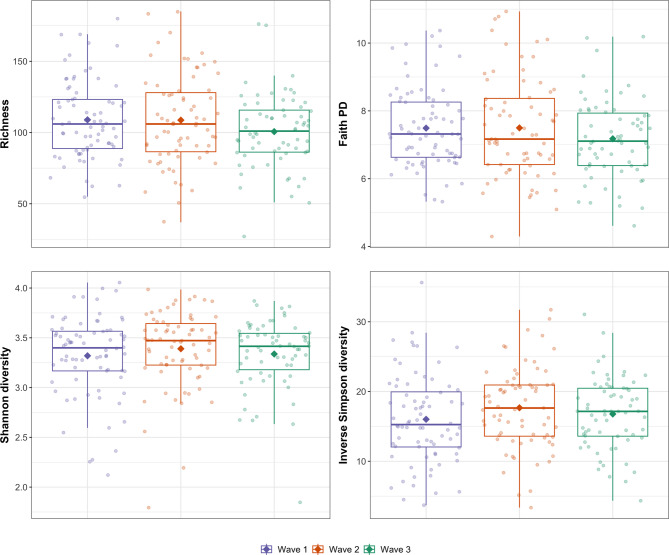
Boxplot showing alpha diversity metrics in Labrador Retriever puppy faecal samples by wave of collection

Samples collected during wave three were associated with a decrease in richness (α = -10.61, *P* = 0.024) and Faith’s PD (α = -0.447, *P* = 0.032) compared with samples collected during wave one (see Fig. 3).

Coprophagia score was associated with an increase in richness (α = 7.592, *P* < 0.001), Faith’s PD (α = 0.421, *P* < 0.001), the Shannon index (α = 0.080, *P* = 0.003) and the inverse Simpson index (α = 0.909, *P* = 0.039), and contact with dogs was associated with increased Faith’s PD (α = 0.312, *P* = 0.009) and trended toward an increase in richness (α = 4.754, *P* = 0.076). Conversely, contact with horses was associated with a decrease in richness (α = -5.265, *P* = 0.041) and Faith’s PD (α = -0.277, *P* = 0.015).

**Fig. 4:**
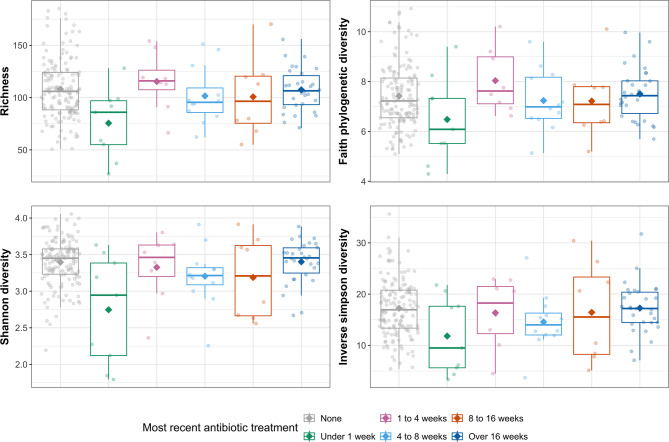
Alpha diversity metrics in Labrador Retriever puppy faecal samples by time since they received antibiotics

Samples from puppies whose owners had reported that they had received oral and/or injected antibiotics within the last week were associated with a substantial reduction in richness (α = -34.18, *P* < 0.001), Faith’s PD (α = -0.950, *P* = 0.018), the Shannon index (α = -0.667, *P* < 0.001) and the inverse Simpson index (α = -5.346, *P* = 0.015) in comparison with samples from puppies whose owners had never reported that they had received oral and/or injected antibiotics (see Fig. 4).

There was a trend toward an increase in the Shannon index (α = 0.255, *P* = 0.054) in puppies that had severe vomiting in the previous 4 weeks and an increase in Faith’s PD (α = 0.580, *P* = 0.046) in puppies that had severe diarrhoea in the previous 4 weeks.

### Beta diversity analyses

PERMANOVA models fit to Jaccard, Bray‒Curtis, unweighted UF and weighted UF distances were used to test for differences in community composition in the variables of interest. These results produced estimates for the r^2^ value, which indicates the degree of community variance explained by the specific variable and associated *P*-values, as shown in Table 3.

**Table 3:** PERMANOVA outputs for beta diversity distances in Labrador retriever puppy faecal samples

Variable	Jaccard		Bray‒Curtis		Unweighted UF		Weighted UF	
	*r* ^2^	*P*	*r* ^2^	*P*	*r* ^2^	*P*	*r* ^2^	*P*
Wave of collection	0.013	*0.001	0.016	*0.002	0.019	*0.001	0.015	*0.031
Sex	0.005	0.818	0.005	0.637	0.005	*0.012	0.008	0.132
Coat colour	0.017	0.862	0.020	0.677	0.022	*0.031	0.022	0.685
Household type	0.016	0.158	0.016	0.392	0.021	0.181	0.013	0.606
Smoking status	0.006	0.700	0.007	0.431	0.006	0.898	0.006	0.191
UK region	0.016	0.608	0.015	0.714	0.022	0.214	0.009	0.888
Area classification	0.011	0.889	0.014	0.751	0.010	0.827	0.013	0.666
Waste consumption scores								
Household	0.004	0.831	0.003	0.803	0.008	*0.002	0.005	0.099
Outdoors	0.004	0.967	0.002	0.917	0.005	0.128	0.004	0.342
Coprophagia	0.006	0.097	0.008	0.121	0.026	*0.005	0.007	0.168
Stress score	0.005	0.471	0.006	0.292	0.004	0.538	0.005	0.637
Contact with other animals scores								
Dogs	0.006	*0.049	0.008	*0.034	0.010	*0.023	0.011	0.062
Cats	0.005	0.278	0.004	0.467	0.008	0.118	0.002	0.701
Horses	0.005	0.337	0.006	0.342	0.012	0.293	0.003	0.563
Farm animals	0.005	0.128	0.005	0.119	0.005	0.833	0.004	0.517
Chickens	0.005	0.581	0.004	0.639	0.004	0.535	0.001	0.638
Antibiotics	0.029	*0.022	0.039	*0.013	0.040	*0.007	0.039	0.115
Vomiting	0.018	0.534	0.018	0.480	0.018	0.563	0.011	0.878
Diarrhoea	0.019	0.392	0.020	0.419	0.019	0.169	0.015	0.759
Total r^2^	0.207		0.239		0.272		0.217	
Individual puppy	0.444	*0.001	0.521	*0.001	0.507	*0.001	0.496	*0.001

UF: UniFrac. *P-values lower than 0.05

Individual dog identity was significant (*P* = 0.001) in all PERMANOVA models and explained between 44.4% and 52.1% of the community variance, depending on the distance metric used. When the effect of individual dog identity was removed, the remaining variables explained between 20.7% and 27.2% of the community variance in the PERMANOVA models collectively, depending on the distance metric used. The variables found to be significantly different in community composition in at least one of the distance measures were wave of collection, sex, colour, household waste score, coprophagia score, dog contact score and time since last receiving oral and/or injected antibiotics. See Sect. 11.2, Additional File 2, for the PCoA ordination of these variables.

Wave of collection explained between 1.3% and 1.9% of the community variation in the Jaccard (r^2^ = 0.013, *P* = 0.001), Bray‒Curtis (r^2^ = 0.016, *P* = 0.002), unweighted UF (r^2^ = 0.019, *P* = 0.001) and weighted UF (r^2^ = 0.015, *P* = 0.031) distances. The sex of the puppies explained 0.5% of the community variation in the unweighted UF (r^2^ = 0.005, *P* = 0.012) distance, and coat colour explained 2.2% of the community variation in the unweighted UF (r^2^ = 0.022, *P* = 0.031) distance. Coprophagia score and household waste score explained 2.6% (r^2^ = 0.026, *P* = 0.005) and 0.8% (r^2^ = 0.008, *P* = 0.002), respectively, of the community variation in the unweighted UF distance. Contact with dogs explained a small amount of community variation in the Jaccard (r^2^ = 0.006, *P* = 0.049), Bray‒Curtis (r^2^ = 0.008, *P* = 0.034) and unweighted UF (r^2^ = 0.010, *P* = 0.023) distances and showed a similar trend in the weighted UF (0.011, *p* = 0.062). Time since last receiving oral and/or injected antibiotics explained between 2.9% and 4.0% of the community variation, with the greatest percentage of any variable (except individual dog identity) in the Jaccard (r^2^ = 0.029 *P* = 0.022), Bray‒Curtis (r^2^ = 0.039, *P* = 0.013) and unweighted UF (r^2^ = 0.040, *P* = 0.007) distances.

For each of the categorical variables, PERMDISP using Jaccard, Bray‒Curtis, unweighted UF and weighted UF distances was performed before ANOVA to test for differences in community dispersion. The estimated adjusted *P-*values (*Q-*values) from all the models are given in Table 4.

**Table 4:** PERMDISP outputs for beta diversity distances in Labrador retriever puppy faecal samples

Variable	Jaccard	Bray‒Curtis	Unweighted UF	Weighted UF
	Q	Q	Q	Q
Wave of collection	*0.002	*0.004	*0.012	*0.021
Sex	0.557	0.454	0.229	0.728
Coat colour	0.612	0.628	0.304	0.767
Household type	0.596	0.628	0.891	0.767
Smoking status	*0.009	0.225	0.891	0.767
UK region	0.612	0.628	0.673	0.767
Area classification	0.612	0.720	0.229	0.767
Antibiotics	0.612	0.225	0.145	0.464
Vomiting	*0.002	0.387	0.229	0.716
Diarrhoea	0.197	0.225	*0.012	0.464

UF: UniFrac. Adjusted *P*-values (*Q*) were estimated. *Q-values lower than 0.05

**Fig. 5:**
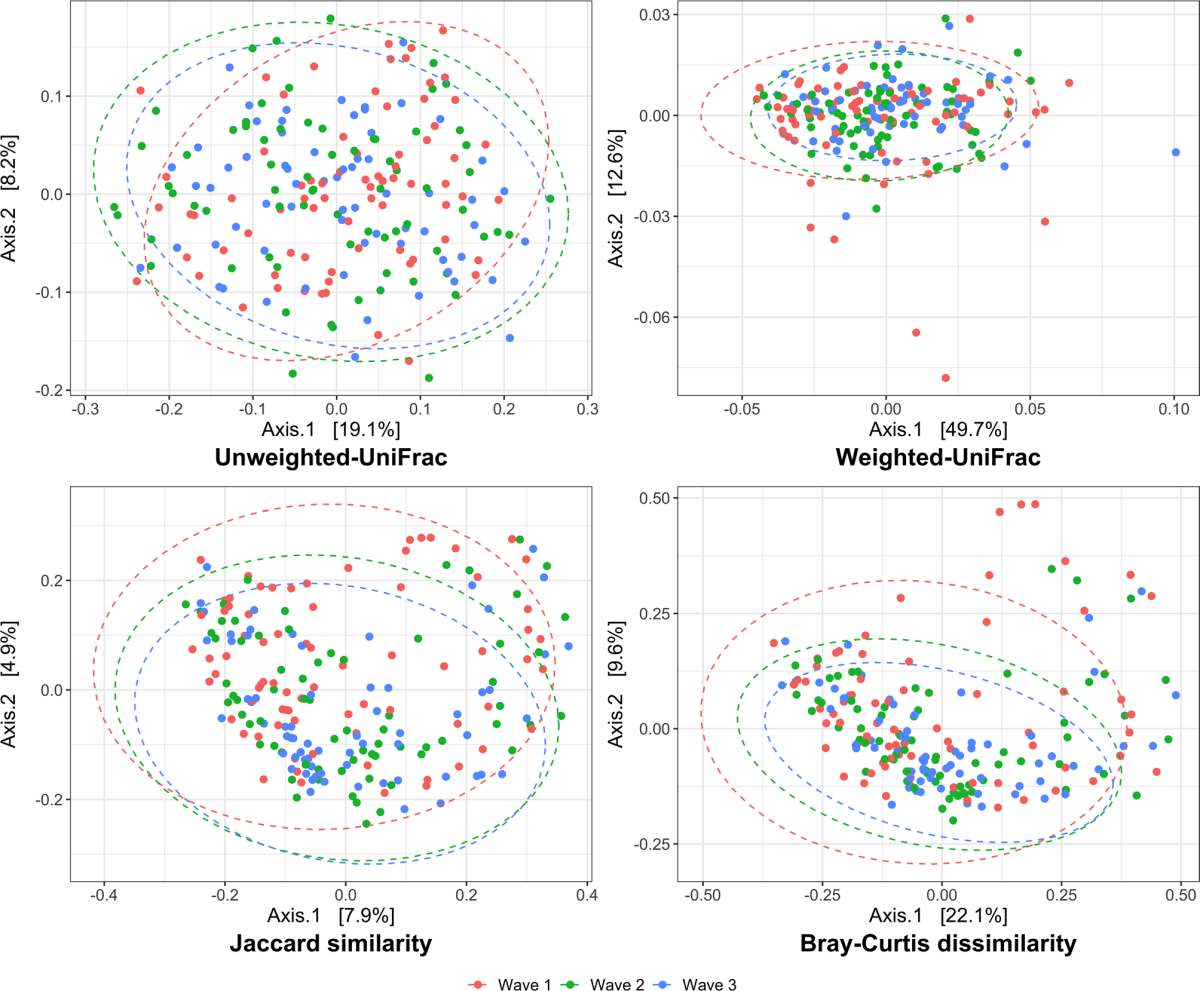
PCoA for beta diversity distances by wave of collection in Labrador Retriever puppy faecal samples

The variables found to be significantly different in community dispersion in at least one of the distance measures were wave of collection, smoking status, time since the last severe vomiting episode and time since the last severe diarrhoea episode. The wave of collection was associated with differences in dispersion in the Jaccard (*Q* = 0.002), Bray‒Curtis (*Q* = 0.004), unweighted UF (*Q* = 0.012) and weighted UF (*Q* = 0.021) distances. The PCoA ordination plotted in Fig. 5 shows that the samples generally became less dispersed as the wave of sample collection increased, or in other words, as the puppies became older.

The only demographic variable associated with community dispersion was smoking status according to the Jaccard index (Q = 0.009). There were also differences in dispersion between vomiting time categories in terms of the Jaccard distance (Q = 0.002) and between diarrhoea time categories in terms of the unweighted UF distance (Q = 0.012).

### Taxonomic analyses

**Fig. 6:**
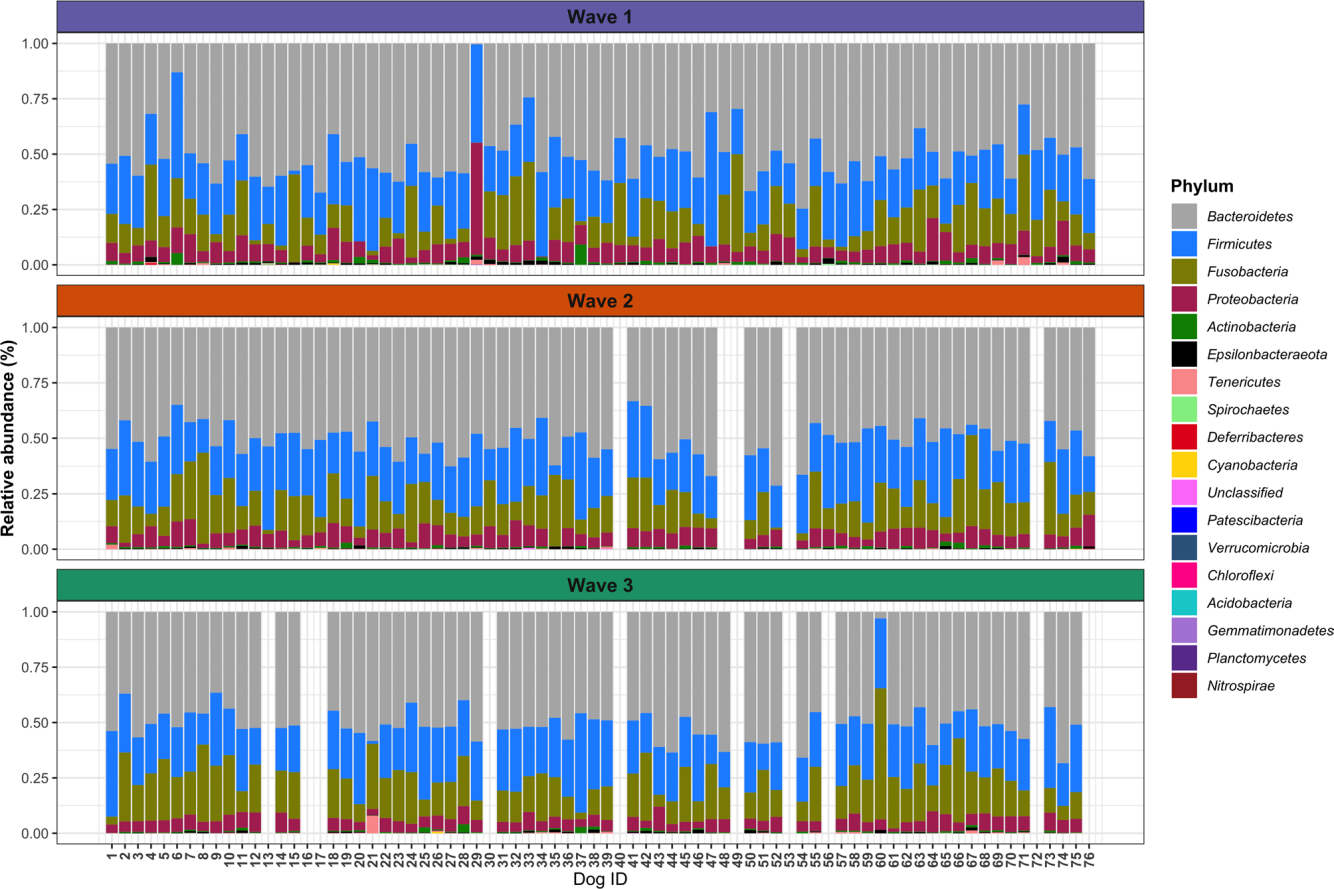
Relative abundance of phyla in individual Labrador Retriever puppy faecal samples by wave of collection

**Fig. 7:**
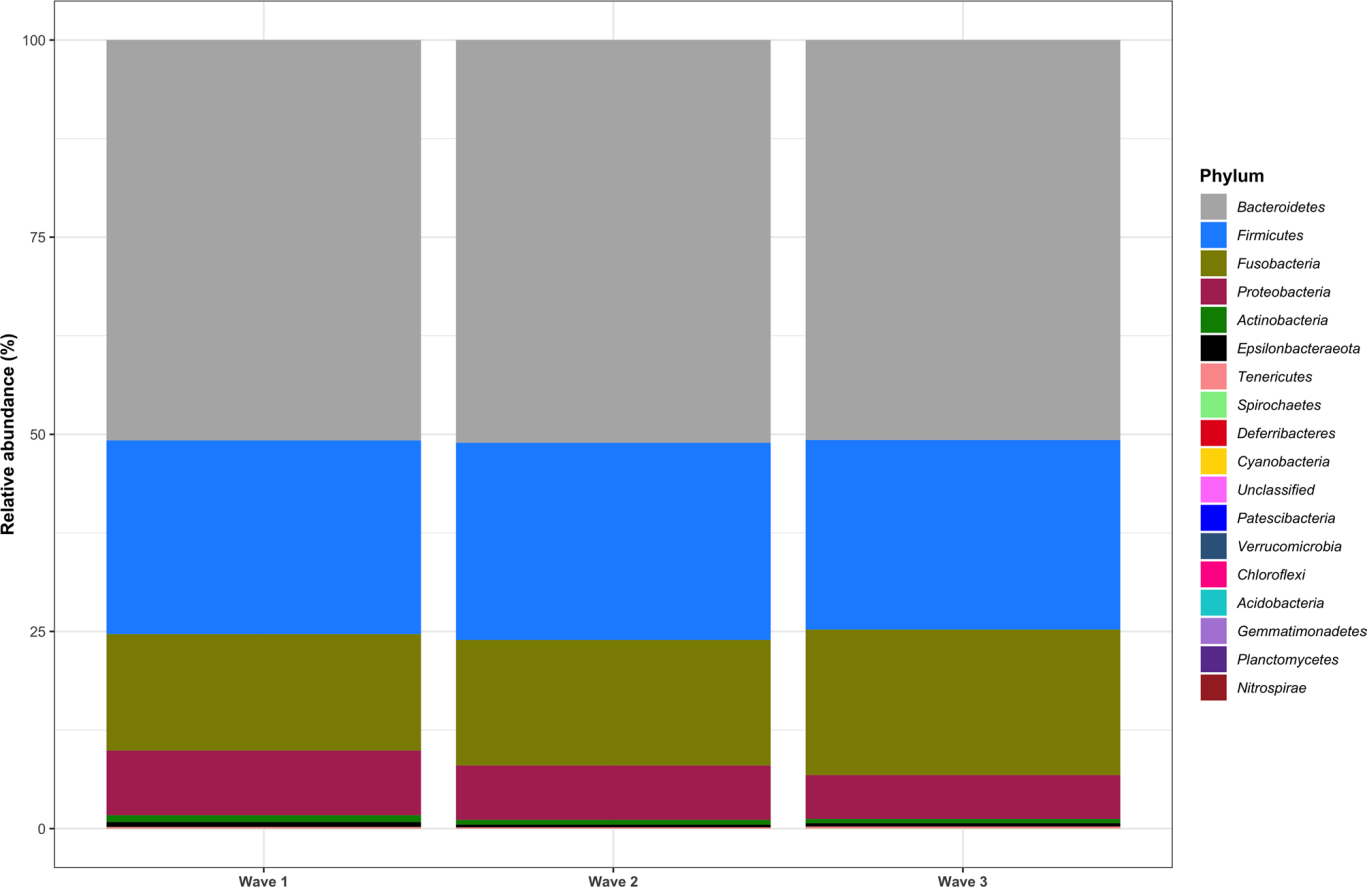
Average relative abundance of phyla in Labrador Retriever puppy faecal samples by wave of collection

The relative abundance and frequency of detection of taxa are summarised by phylum, class, order, family and genus, and all results can be found in Sect. 12, Additional File 2. A total of 17 phyla were classified, and 22 samples contained unclassified phyla (0.004% of the total abundance). The four most abundant phyla were *Bacteroidetes* (50.86%), *Firmicutes* (24.55%), *Fusobacteria* (16.29%) and *Proteobacteria* (6.95%), accounting for 98.65% of the total abundance, and were detected in all the samples. The remaining bacteria included *Actinobacteria* (0.679%, *n* = 209), *Epsilonbacteraeota* (0.465%, *n* = 203), *Tenericutes* (0.166%, *n* = 112), *Spirochaetes* (0.016%, *n* = 51), *Deferribacteres* (0.014%, *n* = 53) and others (see Sect. 12.1, Additional File 2). The relative abundance of phyla was plotted for the faecal samples individually and at each wave of collection and is shown in Figs. 6 and 7, respectively.

A total of 95 families were classified, and 197 samples contained unclassified families (0.868% of the total abundance). The five most abundant families were *Prevotellaceae* (35.83%), *Fusobacteriaceae* (16.29%), *Bacteroidaceae* (14.60%), *Ruminococcaceae* (7.401%) and *Lachnospiraceae* (6.369%), accounting for 80.49% of the total abundance, and were detected in all the samples. All others are described in Sect. 12.4, Additional File 2. The relative abundance of families was plotted for the faecal samples individually and at each wave of collection and are shown in Supplementary Figs. 44 and 45, Additional File 2, respectively.

**Fig. 8:**
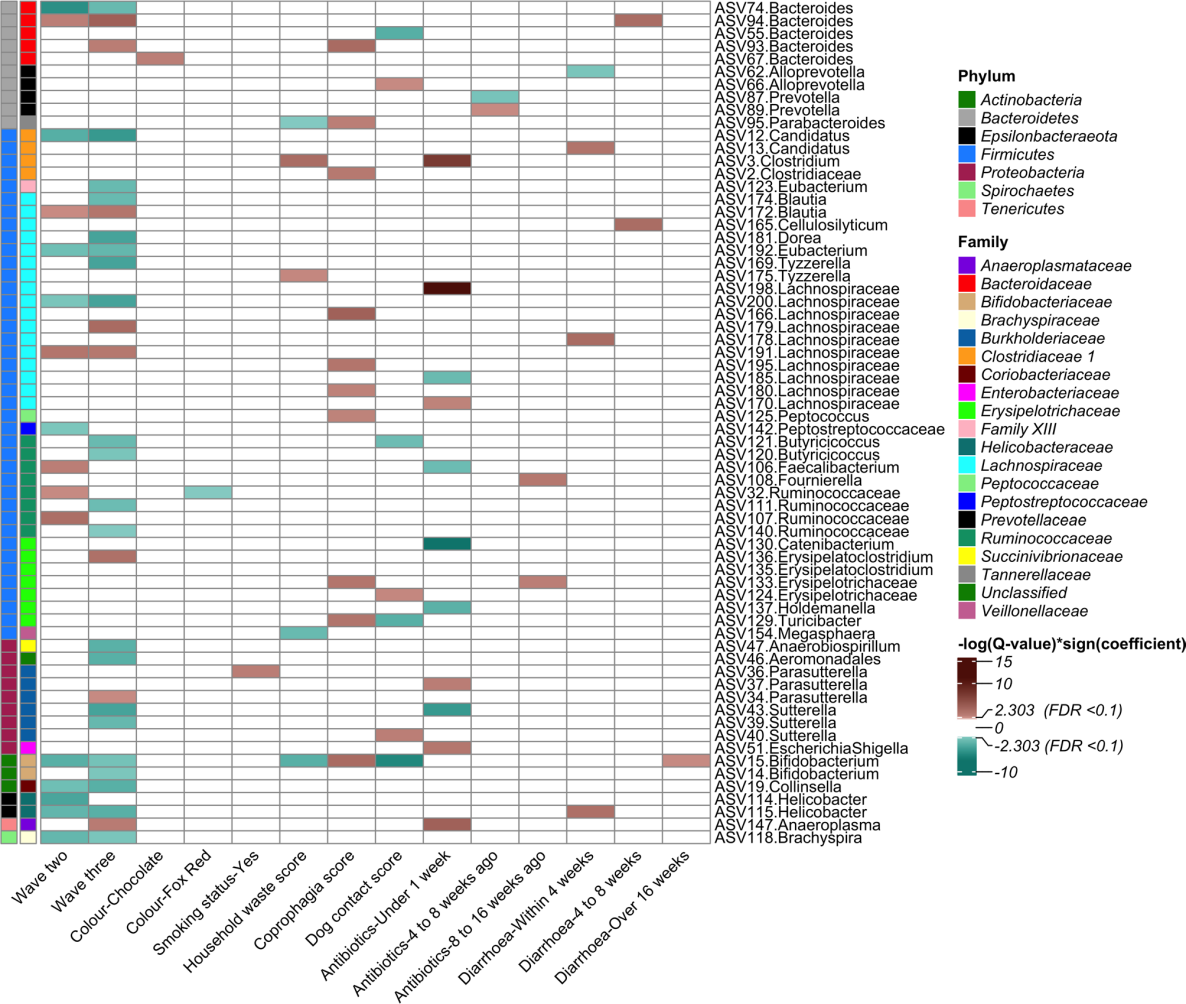
Heatmap showing significantly differentially abundant amplicon sequence variants in Labrador Retriever puppy faecal samples

Differential abundance analyses were performed on the relative abundances of taxa with a prevalence over 10%. MaAsLin was subsequently performed using variables that produced significant results in alpha and beta diversity analyses. Out of 204 models of differential abundance, 31 did not fully converge and were removed from the results. Among the remaining 173 models, 66 were significantly associated with differential abundance in at least one of the following variables: wave of collection, coat colour, smoking status, household waste score, coprophagia score, dog contact score, time since last receiving oral and/or injected antibiotic, time since last severe diarrhoea episode and time since last severe vomiting episode. Most of these ASVs were assigned taxonomy at the genus level, but 15 were assigned taxonomy at three family levels, and one was assigned taxonomy at the order level. To improve the interpretability of the results and aid in comparisons with other research, it is recommended that the reader refer to the full heatmap of all results (see Supplementary Fig. 46, Additional File 2) and classifications of the taxonomy (including phylum, class, order, family and genus) of all significantly abundant ASVs (see Supplementary Table 66, Additional File 2). Figure 8 shows models that demonstrated significant differential abundance, and the results are discussed below.

## Discussion

This study is one of the first longitudinal gut microbiota studies in puppies and the first to investigate canine health factors in addition to a range of novel environmental and health factors. 76 Labrador Retriever puppies enrolled in Dogslife were successfully recruited for the study. Associations were identified between puppies’ gut microbiota and several factors, including wave of sample collection, sex, colour, household smoking status, consumption of household waste, coprophagia, contact with dogs and horses, time since treatment with oral/injected antibiotics, and time since vomiting and diarrhoea.

### Classification

The predominant bacterial phyla in the faecal samples were *Bacteroidetes*, *Firmicutes*, *Fusobacteria*, *Proteobacteria* and *Actinobacteria*, which is in agreement with previous research, although the proportions of these phyla have varied [3, 12, 23]^93^– [95]. Several other phyla were also detected at lower abundances, including *Epsilonbacteraeota* [95], *Tenericutes* [96, 97], *Spirochaetes*, *Deferribacteres* [97], *Cyanobacteria* [98, 99] and seven others, which were classified in fewer than 4% of the samples (see Supplementary Table 59, Additional File 2).

### Individual variation

The greatest source of variation in puppies’ gut microbiota was explained by differences between individual puppies, which explained approximately 25% of the alpha diversity and 50% of the beta diversity. Figure 6 and Figure S36 show that, generally, the relative abundance of taxa varied less between waves of sample collection than between individuals. This finding agrees with previous human research that has shown that individual differences in microbiome composition between individuals are greater than those within individuals over time [19, 20]. Vilson and colleagues [23] reported that related puppies had more similar faecal microbiota than unrelated puppies did, a finding similarly demonstrated in a study of 11 miniature Schnauzers [1]. Furthermore, breed has been shown to impact the composition of the faecal canine gut microbiota [26]. This is particularly significant, as this was a single breed study, so it was less likely to be affected by large differences in host genetics. In a review of the development of the gut microbiota in dogs, Garrigues and colleagues [12] highlighted that many canine microbiome studies do not account for interindividual variability, which leads to poor reproducibility between studies. When treating dysbiosis in puppies, it may also be appropriate to consider personalised treatments or interventions that allow for variation in individuals response.

### Age of puppies

The age of the puppies at the time of faecal sample collection was associated with differences in alpha and beta diversity, as well as differential abundances of various taxa. A reduction in richness and Faith’s PD, but not in the Shannon or inverse Simpson distances, was observed between the first wave of sample collection (puppies aged 3.5 months) and the third wave (puppies aged 12.5 months). Significant differences in community composition and dispersion were noted between collection waves, with PCoA indicating that dispersion decreased as puppies aged.

Studies on the development of the gut microbiota in puppies have been limited and contradictory. Rapid shifts in the diversity and composition of the gut microbiota of puppies occur before weaning, and Garrigues and colleagues suggested that the gut microbiota of dogs stabilises after this [12, 22]. A cohort study of German Shepherd puppies [23] did not report any differences in the Shannon index of faecal samples from puppies aged seven weeks up to 15 to 18 months, whereas a Japanese cross-sectional study demonstrated decreased diversity with ageing in purebred Shiba Inu dogs [53]. Conversely, a small study of 12 Beagles revealed that alpha diversity metrics increased between 20 and 28 weeks of age, after which they remained relatively stable [21]. However, the latter two studies were conducted in controlled environments, limiting their external validity. The DAP reported a decrease in diversity and shifts in the composition of the canine gut composition with age [18], which aligns with the results from our study, although interindividual variability may have impacted their results.

Research in humans has shown that alpha and beta diversity increase from birth into adulthood [53, 100, 101]. The early life experiences of puppies differ from those of humans: puppies are typically removed from their mothers during infancy and placed in new environments, potentially leading to larger shifts in the microbiome at an earlier relative age than in children. Dogslife puppies were first sampled at 3.5 months, soon after the typical age for rehoming, but they were likely to have enough time to experience various new environmental exposures. This might explain why the dispersion of microbiota stabilised over time, as puppies adjusted to their new environments.

In this study, the relative abundance of taxa and differential abundance analyses revealed considerable changes in composition with age, with more significant changes evident at 12.5 months than at 7.5 months and 3.5 months. Within the *Bacteroidetes* phylum, there were both increases and decreases in the differential abundance of taxa within the *Bacteroidaceae* family (genus *Bacteroides*) at both 7.5 and 12.5 months of age. *Bacteroides* play a complex role in health, acting as beneficial microbes, commensals, and opportunistic pathogens depending on the species [102], so differences between genera may reflect different functions of the taxa identified. A similar pattern was observed in the *Firmicutes* phylum, with more changes in differential abundance as puppies aged. There was a decreased differential abundance of the *Clostridiaceae 1* family (genus *Candidatus Arthromitus*) at both 7.5 and 12.5 months of age. *Candidatus Arthromitus* are segmented filamentous bacteria known for their immunomodulatory effects [103].

Within the *Lachnospiraceae* family, there were mixed associations between age and differential abundance. In the *Ruminococcaceae* family, there were only increases in differential abundance at 7.5 months, whereas at 12.5 months, there were decreases. Decreased abundance of *Ruminococcaceae* and *Lachnospiraceae* has been implicated in the development of IBD in dogs [36, 104, 105], which are typically diagnosed in middle age and are associated with early life environmental exposures [106]. Within the *Actinobacteria* phylum, the abundances of the families *Bifidobacteriaceae* (genus *Bifidobacteria*) and *Coriobacteriaceae* (genus *Collinsella*) decreased significantly at both 7.5 and 12.5 months of age. *Collinsella* has been implicated in the immune response to IBD in dogs [107], whereas *Bifidobacteria* are widely considered beneficial bacteria in humans [108]. *Bifidobacteria* have been demonstrated to be beneficial supplements for dogs [109]. Our results align with those of Masouka and colleagues [110], who successfully isolated *Bifidobacterium* in laboratory-housed Beagle puppies aged approximately two and seven weeks, but could not isolate it in dogs aged two years or older. In contrast, a cross-sectional multi-breed study reported a greater abundance of *Bifidobacteria* in adult dogs (5 to 7 years) than in junior dogs (less than 2 years) [111]. Breed or individual variation may explain these differences. *Bifidobacterium* has been shown to be affected by diet in dogs [112], suggesting that dietary changes in the first year of life may be partially responsible.

In addition to changes in beneficial bacteria with age, there were changes in bacteria that can cause disease. Within the *Proteobacteria* phylum, known for including opportunistic pathogens, the family *Succinivibrionaceae* showed a decrease in the genus *Sutterella* and an increase in the genus *Parasutterella* at 12.5 months but not at 7.5 months. Increases in *Sutterella* have been associated with acute haemorrhagic diarrhoea [36] but were not linked to gastrointestinal signs in our study. Within the *Epsilonbacteraeota* phylum and *Helicobacteraceae* family, the genus *Helicobacter* decreased, and within the *Spirochaetes* phylum and *Brachyspiraceae* family, *Brachyspira* decreased in puppies aged 7.5 and 12.5 months compared with puppies aged 3.5 months. The role of *Helicobacter* as both a pathogen causing acute infection and a chronic inflammatory agent in humans is well established [113] but remains controversial in dogs [114–117]. It is notable that in our study, *Helicobacter* had increased differential abundance in puppies that had experienced severe diarrhoea in the last four weeks. *Brachyspira* is a zoonotic pathogen that is particularly harmful to pigs and chickens but is thought to be asymptomatic in dogs [118]. In agreement with our results, one study reported that dogs under one year of age were at greater risk of shedding *Brachyspira* [119]. These results have implications for farmers, who may allow their puppies to mix with farm animals, including pigs and chickens.

In summary, our analysis revealed that the canine gut microbiota is still developing over the first year of life, with most compositional shifts occurring within specific families rather than phyla. The implications of these changes on canine health are not yet fully understood but warrant further investigation.

### Sex

Sex was associated with small changes in community composition, which contrasts with previous canine research that reported no differences [21, 120]. However, in one of these studies, there were some differences in the relative abundance of taxa between sexes [21] and another study reported an association between gut microbiota community differences and dogs’ neutering status [24]. This suggests an endocrine interaction with the microbiome, which has been demonstrated in animal models [121] and is associated with disease in humans [122]. Interestingly, the variation in community composition due to sex was found only in the UF distance. This suggests that the phylogeny of the microbiota driving the difference was important, which is what one might expect when considering the potential impact of hormonal mechanisms. Alternatively, differences may be due to anatomical differences due to the sex of the dog, such as exposure to vaginal microbiota in female dogs (for example, during grooming) but not male dogs.

### Coat colour

To our knowledge, this is the first study to associate coat colour with differences in the gut microbiota composition of puppies while controlling for other demographic and environmental factors. This raises the question of whether certain coat colours could confer a physiological or microbiological advantage. In Labrador Retrievers, yellow and fox red coat colour are caused by the production of phaeomelanin, which is stimulated by a mutation in the melanocortin 1 receptor (*MC1R*) gene [123]. Similarly, coat colour in squirrels is driven by the *MC1R* gene [124], and associations between the coat colour phenotype and the diversity and variation of the faecal microbiota have been reported [125]. Furthermore, the *MC1R* gene in combination with gut microbes has been associated with inflammatory processes [126, 127]. Interestingly, increases in the genus *Bacteroides* were associated with chocolate-coloured puppies. It has been postulated that *Bacteroides* play a role in inhibiting obesity in dogs [128], to which chocolate Labrador Retrievers are particularly susceptible [129]. Like sex, variation in community composition due to colour was found only in UF distances, suggesting that phylogeny was important.

In this study, fox red and yellow puppies were treated as separate groups. A subsequent analysis could investigate whether the strength of associations with coat colour and gut microbiota diversity and composition increases when fox red and yellow dogs are combined, indicating a genetic component of the differences.

### Consumption of household waste and coprophagia

Our results revealed that coprophagia and the consumption of household waste were associated with the composition of the canine gut microbiota. Dogs, being carnivorous scavengers in their natural state, commonly display behaviours such as consuming inedible objects or waste items. Coprophagia, the act of consuming faeces, was associated with increased alpha diversity across all the distance metrics and explained community variation measured by the unweighted UF distance.

Differential abundance analysis revealed decreases in the genera *Parabacteroides* (phylum: *Bacteroidetes*, family: *Tannerellaceae*) and *Bifidobacterium* (phylum: *Actinobacteria*, family: *Bifidobacteriaceae*) in puppies that consumed household waste, both of which are typically viewed as beneficial to dog health. In contrast, coprophagia led to increases in these and several other genera within the phyla *Bacteroidetes* and *Firmicutes*, which are also generally perceived as beneficial [12, 130]. Our results are in agreement with the results from the DAP reported in preprint, which revealed increased alpha diversity and microbial community structure in dogs that displayed coprophagy [18].

Coprophagia is generally considered harmless, although it is deemed very unpleasant for owners [131]. It has been implicated in rare case reports of poisoning [132], and treatment methods have had limited success [133, 134]. A common perception is that coprophagia has a nutritional cause, but there is no evidence to substantiate this claim. One study reported that coprophagy did not influence digestibility, faecal pH, or fermentative metabolites [135]. Another study associated coprophagia with cohabitation with another coprophagic dog but reported no associations other factors, such as sex, diet, lifestyle, or reproductive status [136]. Other studies have positively associated coprophagia with helminthic burden [137], neutering [137, 138], stress [138], age [139], exercise [139] and being a ‘greedy eater’ [133], but little is known about the background cause.

A proposed evolutionary drive of coprophagia is that wolves may have eaten fresh faeces as a parasite defence strategy to remove ova from the den prior to becoming infectious and therefore protecting their pups [133]. An alternative theory is that coprophagia may be an adaptive mechanism. The authors of a study that associated coprophagia with both behavioural and non-behavioural medical diagnoses [140] suggested that canine coprophagia is indicative of microbiome inoculation. Bacteria are recycled by some animals, such as rodents and lagomorphs, but this is not the case for dogs [138], which may explain why associations of coprophagia with the canine microbiome had not been previously suggested.

### Contact with other dogs and horses

Contact with dogs was associated with increased alpha diversity in Faith’s PD, whereas contact with horses had the opposite effect. Contact with dogs also explained community variation measured by several distances, as confirmed by differential abundance analyses, while contact with horses did not. A higher dog contact score was linked to a decreased abundance of several beneficial genera, including *Turicibacter* (phylum: *Firmicutes*, family: *Erysipelotrichaceae*), which is protective against dysbiosis in dogs [38], as well as the beneficial genera *Bifidobacterium* and *Bacteroides*. An increase in Faith’s PD without changes in other alpha diversity metrics indicates a shift toward greater phylogenetic diversity, highlighting the presence of more evolutionary lineages rather than changes in species richness or evenness. This suggests that increased competition from other strains may cause the differential abundance of these specific taxa to decrease in puppies with more frequent socialisation, while less prevalent strains of the same genera were not included in the analysis.

The decreased alpha diversity in puppies with more frequent contact with horses is challenging to interpret. It is possible that the owners of these puppies have different sociodemographic characteristics or display different behaviours, which impact their puppies’ gut microbiota. The “farm-like effect” in humans is associated with rural living and interaction with farm animals with positive health outcomes [56], and some research has indicated the protective effects of farm animals against atopy in dogs [38] and of horses against diarrhoea in dogs [141]. The reasons for these protective mechanisms are unclear, but it is possible that the canine microbiome plays a role via its ability to modulate the immune system.

A limitation of this study is that owners may have interpreted “contact” with animals differently: some owners may have recorded this only when their puppies physically touched another animal, whereas others may have noted this when their puppies were merely in the vicinity of another animal. This variation in interpretation makes understanding our results difficult, as it is possible that differences in owner perceptions caused bias within the data. Therefore, future research should aim to investigate specifically how frequent and how close the interaction must be with other animals to influence the gut microbiota of dogs.

### Antibiotics

The estimated prevalence of oral and injected antibiotic usage in our study was high, increasing from approximately 21% at 3.5 months of age to 44% at 12.5 months. This aligns with recent research using primary care and referral data in the U.S., which estimated the point prevalence of systemic antibiotic usage in 1,987 dogs to be 26.1% [142]. Antibiotic usage was associated with reductions in alpha diversity, changes in microbiota community composition, and shifts in the differential abundance of taxa across the phyla *Firmicutes*, *Proteobacteria*, and *Tenericutes*. However, these changes in diversity and composition appeared to be relatively short-lived, with most alterations occurring within one week of puppies receiving an oral antibiotic.

This contrasts with research in humans, which has demonstrated that antibiotics have lasting effects on the microbiome [43]. Similarly, studies have shown sustained dysbiosis up to 84 days after dogs were administered antibiotics in a randomised, controlled trial [44] and in a prospective, non-randomised controlled study, where *Fusobacteria* remained reduced four weeks after metronidazole administration [45]. It is likely that methodological differences caused these disparities. The former studies administered long durations (three weeks and two weeks) of the same dose and type of antibiotic to previously healthy dogs. In contrast, Dogslife puppies received different types, durations, and doses of antibiotic treatments in response to naturally occurring illnesses. These factors may have caused variability in the response of the gut microbiota to the antibiotics, which was not analysed in this study. The long-term consequences of antibiotics on the gut microbiota for canine health remain unclear. However, achieving shorter-term effects of antibiotics on the microbiota is a desirable outcome for dogs, humans, and other species, and warrants further investigation.

### Vomiting and diarrhoea

The observed prevalence of severe vomiting in puppies increased from approximately 7% at 3.5 months to 32% at 12.5 months. Similarly, the prevalence of severe diarrhoea increased from approximately 30% at 3.5 months to 56% at 12.5 months. These findings align with previous research indicating high morbidity of gastrointestinal symptoms in dogs [31, 32].

Puppies that experienced diarrhoea in the last four weeks had an increase in alpha diversity, specifically in Faith’s PD. There were significant changes in community dispersion related to duration since the puppies experienced severe vomiting and diarrhoea. Other studies have also reported links between canine gastrointestinal microbial changes and both acute [36, 39, 40] and chronic [41] diarrhoea. Differential abundance analysis revealed several changes in taxa within puppies that had diarrhoea, with most changes occurring within the first four weeks after the episode. *Helicobacter* was associated with increased reports of diarrhoea in the last four weeks. Both healthy and diarrhoeic dogs carry various species of *Helicobacter* [114–117], making its association unclear. However, given its well-established role in human pathogenesis [113], our results suggest that dog owners should be cautious during their puppies’ episodes of diarrhoea, as the zoonotic risk is not fully understood. The role of *Helicobacter* in the microbiome warrants further investigation.

### Limitations

We aimed to characterise the gut microbiota of puppies over their first year of life. However, recruitment through Dogslife owners, who are typically not breeders, meant that puppies were over three months old by the time they could be recruited, missing many major shifts in the canine gut microbiota. Questionnaire data are limited by sample bias [143], as Dogslife owners who participated in this gut microbiota study may differ from the general UK dog owner population in observance, experience, access to veterinary information, and other demographic factors. Additionally, Dogslife focuses on Labrador Retrievers, so results may not be generalisable to other breeds. Social desirability bias [144] may have influenced responses, particularly regarding stress levels in puppies, as owners might report lower stress due to perceived social norms or difficulty in judging canine behaviours. Previous Dogslife studies [145] have attempted to address recall bias [146], but such methods introduce subjective cut-offs and limit the data available, which was not deemed appropriate in the current study. Attrition bias^164^ was possible but was likely minimised because of high retention rates (91.57%). Finally, the cleaning, categorising and coding of data from questionnaires is subjective and relies on the expertise and opinions of the person performing the task.

Various biases affect microbiome research methodologies. Precautionary measures were taken to minimise and monitor bias. Mock community samples subjected to PCR and 16 S rRNA gene sequencing misrepresented the abundance of certain phyla in our study, impacting comparisons with other studies. While 16 S rRNA sequencing is cost-effective, it does not infer taxon functionality. Higher sampling depth could provide more insights into microbiota diversity and composition. This study focused on bacterial characterisation of the canine gut microbiota, acknowledging the importance of other organisms and systems to health and the limitations of using faecal samples alone.

## Conclusion

This longitudinal study provides novel insights into the development of the gut microbiota in puppies, highlighting the influence of multiple factors including age, sex, coat colour, environmental exposures, diet, and recent health events. Notably, individual variation accounted for a substantial proportion of the observed microbial diversity, approximately 25% of alpha diversity and 50% of beta diversity, emphasising the importance of longitudinal designs in microbiome research. These findings suggest that cross sectional studies may be limited in their ability to detect group level differences due to high interindividual variability. Variation in the gut microbiota was primarily associated with the individual, although the cause of this variation (such a host genetics or other factors) was not determined in this study.

The results also have potential implications for comparative medicine, particularly in understanding host microbiota interactions, the transient effects of antibiotics in Labrador Retrievers, and the role of specific taxa such as *Helicobacter*. Future research should aim to clarify the mechanisms underlying these associations and explore strategies to mitigate adverse health outcomes. A deeper understanding of the canine gut microbiota may inform veterinary practice and contribute to the development of targeted interventions for both animal and human health.

## Supplementary Information

Below is the link to the electronic supplementary material.

Supplementary Material 1: Additional File 1: Dogslife digestive health questionnaire

Supplementary Material 2: Additional File 2: Supplementary results for ‘The gut microbiota of Labrador Retriever puppies: a longitudinal cohort study’

Supplementary Material 3: Additional File 3: Supplementary code for ‘The gut microbiota of Labrador Retriever puppies: a longitudinal cohort study’

Supplementary Material 4; Additional File 4: Data cleaning and processing for ‘The gut microbiota of Labrador Retriever puppies: a longitudinal cohort study’

## Data Availability

The datasets generated during and/or analysed during the current study are available in the Edinburgh University Datashare repository at [https://doi.org/10.7488/ds/3857](https:/doi.org/10.7488/ds/3857) [65].

## Abbreviations

ANOVA: Analysis of variance
ASV: Amplicon sequence variant
BH: Benjamini and Hochberg
DAP: Dog Aging Project
DHQ: Digestive health questionnaire
HMP: Human Microbiome Project
ICC: Intraclass correlation coefficient
MaAsLin: Microbiome multivariable association with linear models
MC1R: Melanocortin 1 receptor gene
PERMANOVA: Permutational multivariate analysis of variance
PERMDISP: Permutational multivariate analysis of dispersion
rRNA: Ribosomal RNA
PCoA: Principal coordinates analysis
PCR: Polymerase chain reaction
PD: Phylogenetic diversity
UF: UniFrac
