# Supplementary material for: The gut microbiota of Labrador retriever puppies: a longitudinal cohort study
Source: Anim Microbiome. 2025 Oct 17;7:108. doi: 10.1186/s42523-025-00464-2 (PMC12534972; doi:10.1186/s42523-025-00464-2)
Supplement: Supplementary file 1 — Supplementary Material 1: Additional File 1: Dogslife digestive health questionnaire [file 42523_2025_464_MOESM1_ESM.docx]

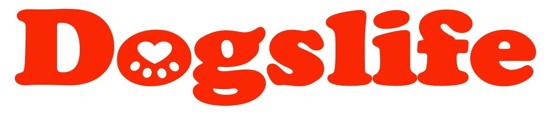
 **
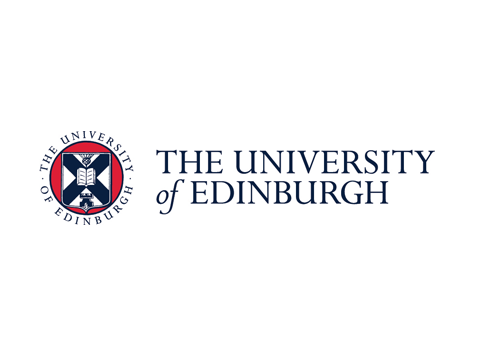
**

**Puppy Digestive Health Questionnaire**

Thank you for participating in the Dogslife project. We are currently investigating factors that cause gut problems (such as diarrhoea and vomiting) in Labrador Retrievers. As your puppy is still very young, it is difficult to tell whether he/she will develop stomach problems later on in life. By gathering information about your puppy as he/she grows up, we will be able to find out more about what keeps dogs guts healthy. We would be very grateful if you could complete the questionnaire and return it to us in the ‘Safebox’ that you received. Thank you for your time and help.

**(* = please circle the most appropriate answer)**

**1. IMPORTANT: What date did you collect your puppy’s faecal and DNA samples?** *……………………….………..….*

**2. Have you changed your puppy’s diet since you got him/her from the breeder?*** *Yes / No / Don’t know*

If YES,

**What** changes have you made?*…………………………………………………………………………………………..*

**Why** did you make these changes?*……..………………………………………………………………………………..*

**3. How often do you feed your puppy ‘titbits’ or treats (including for training), such as*:**

1. Dog chews *Never / Occasionally / Weekly / Every Few Days / Daily*
2. Dog treats *Never / Occasionally / Weekly / Every Few Days / Daily*
3. Bones *Never / Occasionally / Weekly / Every Few Days / Daily*
4. Human food *Never / Occasionally / Weekly / Every Few Days / Daily*
5. Other (please specify below) *Never / Occasionally / Weekly / Every Few Days / Daily*

*…...……………………………………………….*

**4. How often does your puppy eat other things (not their regular food or treats) such as*:**

1. Rubbish (e.g. out of a bin) *Never / Occasionally / Weekly / Every Few Days / Daily*
2. Grass/plants *Never / Occasionally / Weekly / Every Few Days / Daily*
3. Inedible objects (e.g. toys, clothes) *Never / Occasionally / Weekly / Every Few Days / Daily*
4. Other animals’ faeces/poo *Never / Occasionally / Weekly / Every Few Days / Daily*
5. His/her own faeces/poo *Never / Occasionally / Weekly / Every Few Days / Daily*
6. Dead animals (e.g carcasses on walks) *Never / Occasionally / Weekly / Every Few Days / Daily*
7. Untreated water (e.g. sea, puddles, rivers) *Never / Occasionally / Weekly / Every Few Days / Daily*
8. Other (please specify below if known) *Never / Occasionally / Weekly / Every Few Days / Daily*

*……………………………………………………*

**5. Do you give your puppy any dietary supplements?*** *Yes / No*

If YES,

Please provide the **name and brand** of the supplement*………………………………………………………………..*

**How often** do you give your puppy this supplement? *…………………………………………………………………..*

**6. As far as you are aware, has your puppy ever been given antibiotics?*** *Yes / No*

If YES,

**Why** were they given antibiotics?*..………………………………………………………………………………..………*

**How** were they given (e.g. oral tablets, skin creams, injections)?..*..……………………………….…………………..*

**When** were they given antibiotics? *……………………………………………….………………………………………*

**For how long** were they treated with antibiotics?*………………………….………………………………….………..*

If known, what was the **name** of the antibiotics? *……………………………………………………………………….*

**7. Which of the following best describes your puppy’s stress levels (please pick one)?***

1. Low (my puppy is seldom stressed)
2. Medium (my puppy is stressed only in specific situations)
3. High (my puppy is often stressed)
4. Very high (my puppy is always stressed)

**8. How often does your puppy have contact with other animals, such as*:**

1. Dogs *Never / Occasionally / Weekly / Every Few Days / Daily*
2. Cats *Never / Occasionally / Weekly / Every Few Days / Daily*
3. Horses *Never / Occasionally / Weekly / Every Few Days / Daily*
4. Cows *Never / Occasionally / Weekly / Every Few Days / Daily*
5. Sheep *Never / Occasionally / Weekly / Every Few Days / Daily*
6. Pigs *Never / Occasionally / Weekly / Every Few Days / Daily*
7. Chickens *Never / Occasionally / Weekly / Every Few Days / Daily*
8. Other (please specify below) *Never / Occasionally / Weekly / Every Few Days / Daily*

*…...……………………………………………….*

**9**. **Since you have owned your puppy, has he/she had any signs of an upset stomach, such as*:**

1. Diarrhoea *Yes / No*
2. Vomiting *Yes / No*
3. Other (please specify below) *Yes / No*

*…...……………………………………………….*

*If you have not done so already, we would be very grateful if you could update this information the next time you visit your Dogslife profile.*

**10**. **Is there anything else you would like to tell us that you think might be relevant to your puppy’s digestive health**?

*……………………………………………………………………………………………………………………………….*
