# Supplementary material for: The gut microbiota of Labrador retriever puppies: a longitudinal cohort study
Source: Anim Microbiome. 2025 Oct 17;7:108. doi: 10.1186/s42523-025-00464-2 (PMC12534972; doi:10.1186/s42523-025-00464-2)
Supplement: Supplementary file 2 — Supplementary Material 2: Additional File 2: Supplementary results for ‘The gut microbiota of Labrador Retriever puppies: a longitudinal cohort study’ [file 42523_2025_464_MOESM2_ESM.html]

Additional File 2: Supplementary results for ‘The gut microbiota of Labrador Retriever puppies: a longitudinal cohort study’


# Additional File 2: Supplementary results for ‘The gut microbiota of Labrador Retriever puppies: a longitudinal cohort study’

- 1 File setup
- 2 Introduction
- 3 Qiime2 analysis of raw 16S rRNA
  sequences
  - 3.1 Data descriptions
    - 3.1.1 Data used
    - 3.1.2 Data produced
  - 3.2 QIIME code
- 4 R analysis of recruitment and
  sampling data, denoised and classified 16S rRNA sequences and
  metadata
  - 4.1 R packages used and R
    setup
  - 4.2 Data decriptions
    - 4.2.1 Data used
  - 4.3 Read in and set up data for
    analysis
- 5 Recruitment, retention and sampling
  and demographics of sample
  - 5.1 Recruitment
  - 5.2 Retention and sampling
    success
  - 5.3 Recruitment, sampling and
    retention overview
  - 5.4 The time taken between each stage
    of sample collection and the age of Dogslife puppies
  - 5.5 Demographic summaries
    - 5.5.1 Sex
    - 5.5.2 Colour
    - 5.5.3 UK region
    - 5.5.4 Area Classification
    - 5.5.5 Household type
    - 5.5.6 Household smoking
      status
    - 5.5.7 Age at each wave
- 6 Summary of 16S rRNA sequencing
  data
- 7 Analysis of control samples
  - 7.1 Comparison of samples and controls
    by number of reads
    - 7.1.1 Comparison of sample types by
      alpha diversity
    - 7.1.2 Comparison of sample types by
      beta diversity
    - 7.1.3 Comparison of sample types by
      taxonomy
  - 7.2 Comparison of repeat canine
    samples
  - 7.3 Evaluating negative control
    samples
  - 7.4 Evaluating mock community
    samples
    - 7.4.1 Species and genera detected in
      mock community samples
    - 7.4.2 Expected VS actual relative
      abundances in mock community samples
- 8 Analysis of laboratory batch
  effects
  - 8.1 Comparison of sample storage
    batch
  - 8.2 Comparison of DNA extraction
    batch
    - 8.2.1 Comparison of samples and
      controls by number of reads
    - 8.2.2 Comparison of DNA extraction
      batch by alpha diversity
    - 8.2.3 Comparison of DNA extraction
      batches by beta diversity
  - 8.3 Comparison of PCR plates
    - 8.3.1 Comparison of samples and
      controls by number of reads
    - 8.3.2 Comparison of PCR plate by alpha
      diversity
    - 8.3.3 Comparison of PCR platees by
      beta diversity
- 9 Contaminant detection and
  removal
  - 9.1 Mitochondria and cloroplast
    removal
  - 9.2 Removal of other contaminants
    using decontam
- 10 Normalisation of samples and
  filtering data
  - 10.1 Normalisation of samples by SRS
    (alternative to rarefraction)
  - 10.2 Remove unneccessary samples (mock
    community samples, repeats, missing questionnaire dogs)
  - 10.3 Summary of questionnaire data
    - 10.3.1 Household waste
    - 10.3.2 Outdoors waste
    - 10.3.3 Coprophagia
    - 10.3.4 Stress
    - 10.3.5 Contact with dogs
    - 10.3.6 Contact with cats
    - 10.3.7 Contact with horses
    - 10.3.8 Contact with Farm
      animals
    - 10.3.9 Contact with Chickens
    - 10.3.10 Antibiotics
    - 10.3.11 Vomiting
    - 10.3.12 Diarrhoea
- 11 Statistical analysis
  - 11.1 Alpha diversity
    - 11.1.1 Richness (observed number of
      ASVs)
    - 11.1.2 Phylogentic diversity (Faith
      PD)
    - 11.1.3 Shannon diversity
    - 11.1.4 Simpson diversity
    - 11.1.5 Combined results
    - 11.1.6 Boxplot of alpha diversity by
      wave of sample collection
    - 11.1.7 Boxplot of alpha diversity by
      coprophagia score
    - 11.1.8 Boxplot of alpha diversity by
      contact with dogs
    - 11.1.9 Boxplot of alpha diversity by
      contact with horses
    - 11.1.10 Boxplot of alpha diversity by
      antibiotic treatment
    - 11.1.11 Boxplot of alpha diversity by
      diarrhoea
  - 11.2 Beta diversity
    - 11.2.1 PERMANOVA
    - 11.2.2 PERMDISP
    - 11.2.3 PCoA of beta diversity by wave
      of sample collection
    - 11.2.4 PCoA of beta diversity by dog
      sex
    - 11.2.5 PCoA of beta diversity by dog
      colour
    - 11.2.6 PCoA of beta diversity by
      household waste score
    - 11.2.7 PCoA of beta diversity by
      coprophagia score
    - 11.2.8 PCoA of beta diversity by dog
      contact score
    - 11.2.9 PCoA of beta diversity by
      antibiotic treatment
- 12 Taxonomy of canine samples
  - 12.1 Phylum
  - 12.2 Class
  - 12.3 Order
  - 12.4 Family
  - 12.5 Genus
- 13 Differential abundance analysis
  - 13.0.1 Get the full taxonomy of
    differentially abundant ASVs

# 1 File setup

# 2 Introduction

This file is an additional file to the main manuscript entitled “The
gut microbiota of Labrador Retriever puppies: a longitudinal cohort
study”. It contains the programmatic code used for all 16S rRNA sequence
denoising, cleaning and processing, exploratory and statistical
analysis, key result outputs and figure generation of the manuscript. It
also contains information about mock community, negative control
samples, repeat canine samples, laboratory batch effects and model fits
that are not contained in the manuscript. It does not contain details
about study design, the reasoning behind many of the methods used or
explanation or discussion of key results. Please refer to the main
manuscript for more information on these matters.

# 3 Qiime2 analysis of raw 16S rRNA sequences

This part of analysis denoised the raw 16S rRNA reads and
phylogenetic and taxonomic classification was performed. This code was
not run within an RStudio environment but was implemented within a
qiime2-2019.10 environment on a server cluster.

## 3.1 Data descriptions

### 3.1.1 Data used

- The original raw 16S rRNA sequences - “data/raw\_16S\_data/”
- A manifest file containing the file locations of the raw 16S rRNA
  sequences - “data/DOGSLIFE\_MANIFEST.txt”
- The SILVA SSU Ref NR dataset V.132 - downloaded from https://www.arb-silva.de/ and stored in
  “data/silva”

### 3.1.2 Data produced

- Denoised 16S rRNA data with phylogenetic analysis performed and
  taxonomic classifiers assigned - “QIIME2\_results/”

## 3.2 QIIME code

# 4 R analysis of recruitment and sampling data, denoised and classified 16S rRNA sequences and metadata

## 4.1 R packages used and R setup

## 4.2 Data decriptions

### 4.2.1 Data used

All data used in this file is available through the Edinburgh
University Datashare and can be publicly accessed at https://datashare.ed.ac.uk/handle/10283/4866.

- Data about the recruitment of dogs to the study -
  “data/microbiome\_study\_recruitment\_data.csv”
- Data about the sample collections -
  “data/microbiome\_study\_sample\_collection\_data.csv”
- Denoised 16S rRNA data with phylogenetic analysis performed and
  taxonomic classifiers assigned (in .qza format for use within a phyloseq
  object):
  - “data/QIIME2\_results/denoised\_table.qza”
  - “data/QIIME2\_results/rooted-tree.qza”
  - “data/QIIME2\_results/classified\_taxonomy.qza”
- Cleaned metadata file containing sample information and demographic,
  environmental and health information about dogs in the study (in .txt
  format for use within a phyloseq object) -
  “data/microbiome\_study\_cleaned\_metadata.txt”
- Cleaned metadata file containing sample information and demographic,
  environmental and health information about dogs in the study (in .csv
  format for general use) -
  “data/microbiome\_study\_cleaned\_metadata.csv”

## 4.3 Read in and set up data for analysis

# 5 Recruitment, retention and sampling and demographics of sample

## 5.1 Recruitment

There were 363 considered for inclusion into the study. There were
205 dogs that met the criteria to be targeted for the study. There were
83 dogs that were recruited.

Percentage of total pop who were recruited: 22.865. Percentage of
total pop who were targeted: 56.4738. Percentage of targeted pop who
were recruited: 40.4878.

**Supplementary Table 1: Inclusion criteria for dogs into the gut
microbiota study**

| Inclusion status | Number | Percentage |
| --- | --- | --- |
| Admin error | 3 | 0.826 |
| Include | 205 | 56.474 |
| Not contactable | 12 | 3.306 |
| Not healthy | 3 | 0.826 |
| Too old | 140 | 38.567 |

**Supplementary Table 2: Number of dogs targeted for inclusion
into the study and method of owner contact**

| 1st mode of contact | Agreed to participate at 1st contact | 2nd mode of contact | Agreed to participate at 2nd contact | Number | Percentage |
| --- | --- | --- | --- | --- | --- |
| Email | FALSE | No contact | FALSE | 118 | 57.561 |
| Email | FALSE | Phone | TRUE | 2 | 0.976 |
| Email | TRUE | No contact | FALSE | 57 | 27.805 |
| Phone | FALSE | Email | FALSE | 4 | 1.951 |
| Phone | FALSE | Email | TRUE | 9 | 4.390 |
| Phone | TRUE | No contact | FALSE | 15 | 7.317 |

**Supplementary Table 3: Number of dogs targeted into for
inclusion into the study and agreement to participate at 1st
contact**

| 1st mode of contact | Agreed to participate at 1st contact | Number | Percentage |
| --- | --- | --- | --- |
| Email | FALSE | 120 | 58.537 |
| Email | TRUE | 57 | 27.805 |
| Phone | FALSE | 13 | 6.341 |
| Phone | TRUE | 15 | 7.317 |

**Supplementary Table 4: Number of dogs targeted into for
inclusion into the study and agreement to participate at 2nd
contact**

| 2nd mode of contact | Agreed to participate at 2nd contact | Number | Percentage |
| --- | --- | --- | --- |
| Email | FALSE | 4 | 1.951 |
| Email | TRUE | 9 | 4.390 |
| No contact | FALSE | 190 | 92.683 |
| Phone | TRUE | 2 | 0.976 |

## 5.2 Retention and sampling success

**Supplementary Table 5: Number of dogs retained in the study at
each wave of sampling**

| Wave | Sampling success | Number | Percentage of recruited | Percentage of sampled |
| --- | --- | --- | --- | --- |
| 1 | yes | 76 | 91.57 | 100.00 |
| 2 | yes | 72 | 86.75 | 94.74 |
| 3 | yes | 66 | 79.52 | 86.84 |

**Supplementary Table 6: Number of dogs not retained in the study
at each wave of sampling and reason**

| Wave | Sampling success | Questionnaire success | Number | Percentage |
| --- | --- | --- | --- | --- |
| 1 | no | no | 6 | 85.71 |
| 1 | no | yes | 1 | 14.29 |
| 2 | no | no | 11 | 100.00 |
| 3 | no | no | 13 | 76.47 |
| 3 | no | yes | 2 | 11.77 |
| 3 | yes | no | 2 | 11.77 |

## 5.3 Recruitment, sampling and retention overview

**Supplementary Figure 1: The recruitment, sampling success and
retention of puppies recruited to Dogslife gut microbiota
study**

## 5.4 The time taken between each stage of sample collection and the age of Dogslife puppies

**Supplementary Figure 2: The time taken between each stage of
sample collection and the age of Dogslife puppies**

## 5.5 Demographic summaries

### 5.5.1 Sex

**Supplementary Table 7: Number of dogs to the study by
sex**

| Sex | Number | Percentage |
| --- | --- | --- |
| Female | 41 | 53.95 |
| Male | 35 | 46.05 |

### 5.5.2 Colour

**Supplementary Table 8: Number of dogs to the study by
colour**

| Colour | Number | Percentage |
| --- | --- | --- |
| black | 27 | 35.53 |
| chocolate | 11 | 14.47 |
| fox red | 16 | 21.05 |
| yellow | 22 | 28.95 |

### 5.5.3 UK region

**Supplementary Table 9: Number of dogs to the study by UK
region**

| UK region | Number | Percentage |
| --- | --- | --- |
| England Midlands and Wales | 28 | 36.84 |
| England North | 13 | 17.11 |
| England South | 23 | 30.26 |
| Scotland | 12 | 15.79 |

### 5.5.4 Area Classification

**Supplementary Table 10: Number of dogs to the study by area
classification**

| Area classification | Number | Percentage |
| --- | --- | --- |
| rural | 28 | 36.84 |
| suburban | 18 | 23.68 |
| urban | 30 | 39.47 |

### 5.5.5 Household type

**Supplementary Table 11: Number of dogs to the study by household
type**

| Household type | Number | Percentage |
| --- | --- | --- |
| Family (one or more adult and one or more children) | 23 | 30.26 |
| More than one Adult | 33 | 43.42 |
| Retired (Single or Couple) | 11 | 14.47 |
| Single Adult | 9 | 11.84 |

### 5.5.6 Household smoking status

**Supplementary Table 12: Number of dogs to the study by household
smoking status**

| Household smoking status | Number | Percentage |
| --- | --- | --- |
| No | 72 | 94.737 |
| Yes | 4 | 5.263 |

### 5.5.7 Age at each wave

**Supplementary Table 13: Age of recruited dogs at each wave of
study**

| Wave | Min | Max | Median | Mean | Lower CI | Upper CI |
| --- | --- | --- | --- | --- | --- | --- |
| 1 | 2.827 | 5.162 | 3.403 | 3.512 | 3.407 | 3.617 |
| 2 | 6.542 | 8.745 | 7.134 | 7.220 | 7.121 | 7.319 |
| 3 | 11.572 | 14.005 | 12.131 | 12.315 | 12.193 | 12.437 |

# 6 Summary of 16S rRNA sequencing data

A summary of the denoised and classified 16S data before filtering is
shown here:

```
## phyloseq-class experiment-level object
## otu_table()   OTU Table:         [ 1814 taxa and 242 samples ]
## sample_data() Sample Data:       [ 242 samples by 37 sample variables ]
## tax_table()   Taxonomy Table:    [ 1814 taxa by 7 taxonomic ranks ]
## phy_tree()    Phylogenetic Tree: [ 1814 tips and 1749 internal nodes ]
```

There are a total of 12106241 reads in the dataset. It is evident
from the number of internal nodes that there are multichotomies -
resolve these:

```
## phyloseq-class experiment-level object
## otu_table()   OTU Table:         [ 1814 taxa and 242 samples ]
## sample_data() Sample Data:       [ 242 samples by 37 sample variables ]
## tax_table()   Taxonomy Table:    [ 1814 taxa by 7 taxonomic ranks ]
## phy_tree()    Phylogenetic Tree: [ 1814 tips and 1813 internal nodes ]
```

# 7 Analysis of control samples

In order to assess contamination during sample processing, storage
and DNA extraction, negative controls (preservative or water) were
processed and stored at the same time as dog faecal samples (approx one
in every four times the samples were stored). One or two negative
control samples were randomly assigned to each of the 10 batches of DNA
extractions and all negative controls were included in the 3 96 well
plates during transportation, PCR and 16S sequencing.

In order to assess the performance of PCR and sequencing techniques,
a positive control sample from a mock microbial community were included
in each of the 3 96 well plates plates during transportation, PCR and
16S sequencing. The mock community was made up of 20 bacterial species
(5% of each) - see (https://www.atcc.org/products/msa-1002#generalinformation).

8 of the canine samples were replicated during the DNA extraction
phase and sent for PCR and 16S sequencing. All repeats were performed in
batch 10 and plate 3. To check that there were no major differences
between the repeats, various different analyses and visualisations were
performed.

## 7.1 Comparison of samples and controls by number of reads

**Supplementary Figure 3: Boxplot with mean (black diamond) of
reads in different sample types**

It is evident that the reads of canine feacal samples and mock
community samples are much higher than negative control sanmples. There
does not appear to be much contamination in negative control samples
(this will be investigated further in downstream analysis).

**Supplementary Table 14: Summary statistics of number of reads in
different sample types**

| Wave | Min | Max | Median | Mean | Lower CI | Upper CI |
| --- | --- | --- | --- | --- | --- | --- |
| canine faeces | 4659 | 96197 | 52056 | 53557.8 | 51950.1 | 55165.5 |
| mock community | 30312 | 42308 | 32901 | 35173.7 | 19492.1 | 50855.2 |
| negative control | 57 | 900 | 197 | 252.1 | 138.4 | 365.7 |

### 7.1.1 Comparison of sample types by alpha diversity

**Supplementary Figure 4: Alpha diversity in different sample
types**

**Supplementary Table 15: Summary statistics of Richness in
different sample types**

| Wave | Min | Max | Median | Mean | Lower CI | Upper CI |
| --- | --- | --- | --- | --- | --- | --- |
| canine faeces | 27 | 186 | 105 | 106.74 | 102.964 | 110.52 |
| mock community | 27 | 32 | 29 | 29.33 | 23.082 | 35.59 |
| negative control | 4 | 28 | 11 | 12.87 | 9.042 | 16.69 |

**Supplementary Table 16: Summary statistics of number of Shannon
diversity in different sample types**

| Wave | Min | Max | Median | Mean | Lower CI | Upper CI |
| --- | --- | --- | --- | --- | --- | --- |
| canine faeces | 1.794 | 4.055 | 3.418 | 3.349 | 3.299 | 3.399 |
| mock community | 2.941 | 2.968 | 2.945 | 2.951 | 2.914 | 2.988 |
| negative control | 1.091 | 3.054 | 2.030 | 1.997 | 1.705 | 2.289 |

**Supplementary Table 17: Summary statistics of number of Faith PD
in different sample types**

| Wave | Min | Max | Median | Mean | Lower CI | Upper CI |
| --- | --- | --- | --- | --- | --- | --- |
| canine faeces | 4.295 | 11.651 | 7.464 | 7.607 | 7.425 | 7.788 |
| mock community | 4.415 | 5.031 | 4.890 | 4.779 | 3.976 | 5.581 |
| negative control | 2.437 | 4.668 | 3.060 | 3.254 | 2.891 | 3.616 |

It is evident that the alpha diversity of canine feacal samples is
much higher than mock community and negative control samples.

### 7.1.2 Comparison of sample types by beta diversity

**Supplementary Figure 5: PCoA using beta diversity metrics of
samples by sample type (raw abundances)**

**Supplementary Figure 6: PCoA using beta diversity metrics of
samples by sample type (relative abundances)**

The 3 sample types look to have different community characteristics,
as expected.

### 7.1.3 Comparison of sample types by taxonomy

**Supplementary Figure 7: Phyla of samples by sample
type**

The classification of taxa between sample types have some
similarities and differences, as expected.

## 7.2 Comparison of repeat canine samples

The reads in the original and repeated samples are shown in the
figure below.

**Supplementary Figure 8: Total reads in repeated dog
samples**

The taxonomy at Phylum level were examined and the percentage change
in relative abundances between the original and repeated sample were
calculated.

**Supplementary Table 18: The minimum, maximum, mean and 95% CI
percentage change in the Phylum relative abundance of eight dog samples
that were repeated**

| Phylum | Minimum | Maximum | Mean | CI Lower | CI Upper |
| --- | --- | --- | --- | --- | --- |
| Bacteroidetes | 0.023 | 11.766 | 3.416 | 0.039 | 6.793 |
| Firmicutes | 0.330 | 7.041 | 3.109 | 1.034 | 5.185 |
| Fusobacteria | 0.005 | 9.284 | 2.405 | -0.115 | 4.925 |
| Proteobacteria | 0.249 | 5.332 | 1.362 | -0.020 | 2.745 |
| Actinobacteria | 0.002 | 1.797 | 0.453 | -0.079 | 0.985 |
| Epsilonbacteraeota | 0.011 | 0.396 | 0.124 | 0.009 | 0.238 |
| Tenericutes | 0.000 | 0.082 | 0.041 | -0.480 | 0.562 |
| Cyanobacteria | 0.025 | 0.043 | 0.034 | -0.079 | 0.148 |
| Deferribacteres | 0.002 | 0.003 | 0.003 | -0.004 | 0.010 |

There were no large mean percentage changes in relative abundance of
Phyla between repeat samples. Therefore, repeats will be removed at
random from the data before all downstream analyses.

## 7.3 Evaluating negative control samples

There were 32 genera detected in negative control samples. The total
raw and relatives abundances of each of the genera in the negative
controls are shown in the table below.

**Supplementary Table 19: The total raw and relative abundance of
genera-level contaminants detected in negative controls and the number
of samples that contained the genera detected**

| Genus | Total raw abundance | Total relative abundance (%) | Number of samples | Detected in non-negatives |
| --- | --- | --- | --- | --- |
| Prevotella 9 | 1704 | 45.067 | 15 | TRUE |
| Fusobacterium | 587 | 15.525 | 15 | TRUE |
| Bacteroides | 368 | 9.733 | 14 | TRUE |
| Alloprevotella | 345 | 9.125 | 11 | TRUE |
| Faecalibacterium | 217 | 5.739 | 12 | TRUE |
| Not classified | 137 | 3.623 | 11 | FALSE |
| Sutterella | 101 | 2.671 | 9 | TRUE |
| Blautia | 59 | 1.560 | 4 | TRUE |
| Hymenobacter | 59 | 1.560 | 1 | FALSE |
| Phascolarctobacterium | 36 | 0.952 | 5 | TRUE |
| Catenibacterium | 30 | 0.793 | 3 | TRUE |
| Megamonas | 23 | 0.608 | 4 | TRUE |
| Holdemanella | 17 | 0.450 | 1 | TRUE |
| Anaerobiospirillum | 17 | 0.450 | 2 | TRUE |
| Deinococcus | 9 | 0.238 | 1 | TRUE |
| Prevotellaceae Ga6A1 group | 8 | 0.212 | 1 | TRUE |
| Cutibacterium | 8 | 0.212 | 2 | TRUE |
| Parasutterella | 6 | 0.159 | 1 | TRUE |
| Oscillibacter | 6 | 0.159 | 1 | TRUE |
| Flavobacterium | 5 | 0.132 | 1 | TRUE |
| Rhizobacter | 5 | 0.132 | 1 | FALSE |
| Collinsella | 5 | 0.132 | 2 | TRUE |
| Nocardioides | 4 | 0.106 | 1 | TRUE |
| Lactobacillus | 4 | 0.106 | 1 | TRUE |
| Streptococcus | 4 | 0.106 | 2 | TRUE |
| Nostoc PCC-73102 | 3 | 0.079 | 1 | FALSE |
| Escherichia-Shigella | 3 | 0.079 | 1 | TRUE |
| Rubellimicrobium | 3 | 0.079 | 1 | FALSE |
| Ralstonia | 2 | 0.053 | 1 | FALSE |
| Pedobacter | 2 | 0.053 | 1 | TRUE |
| Campylobacter | 2 | 0.053 | 1 | TRUE |
| Pseudomonas | 2 | 0.053 | 1 | TRUE |

The top 5 most abundant genera were Prevotella 9, Fusobacterium,
Bacteroides, Alloprevotella, Faecalibacterium

These made up 85.1891 percent of the negative controls and a total of
3221 reads. These were all found in canine or mock community samples,
indicating that the main source of contamination was
cross-contamination.

There were 5 genera that were detected in negative controls that were
not found in canine or mock community samples Hymenobacter, Rhizobacter,
Nostoc PCC-73102, Rubellimicrobium, Ralstonia

These made up 1.9043 percent of the negative controls and a total of
72 reads.

There were genera that were not classified, which made up 3.6234
percent of the negative controls and a total of 137 reads.

The taxonomic classifications at Genus and Phylum level of the
negative samples are shown in the figures below.

**Supplementary Figure 9: Raw abundance of Genera in negative
control samples**

**Supplementary Figure 10: Raw abundance of Phyla in negative
control samples**

## 7.4 Evaluating mock community samples

### 7.4.1 Species and genera detected in mock community samples

All 20 species that were expected to be found in the mock community
were found in all 3 mock community sampples. The lowest level of
classification that could be accurately assigned and whether they were
also detected in canine samples are shown in the table below.

**Supplementary Table 20: The mock community species, lowest level
of classification that could be accurately assigned and which samples
they were detected in**

| Species | Lowest classification detected | Number of samples | Detected in canine samples |
| --- | --- | --- | --- |
| Clostridium beijerinckii | Genus | 3 | TRUE |
| Streptococcus agalactiae | Genus | 3 | TRUE |
| Streptococcus mutans | Species | 3 | TRUE |
| Staphylococcus aureus | Genus | 3 | TRUE |
| Staphylococcus epidermidis | Genus | 3 | TRUE |
| Bacillus pacificus | Genus | 3 | TRUE |
| Bacteroides vulgatus | Genus | 3 | TRUE |
| Escherichia coli | Genus | 3 | TRUE |
| Porphyromonas gingivalis | Species | 3 | TRUE |
| Lactobacillus gasseri | Species | 3 | TRUE |
| Bifidobacterium adolescentis | Genus | 3 | TRUE |
| Acinetobacter baumannii | Genus | 3 | TRUE |
| Rhodobacter sphaeroides | Genus | 3 | FALSE |
| Deinococcus radiodurans | Species | 3 | FALSE |
| Pseudomonas aeruginosa | Genus | 3 | TRUE |
| Enterococcus faecalis | Genus | 3 | TRUE |
| Neisseria meningitidis | Genus | 3 | FALSE |
| Cutibacterium acnes | Genus | 3 | FALSE |
| Schaalia odontolytica/Actinomyces odontolyticus | Species | 3 | TRUE |
| Helicobacter pylori | Genus | 3 | TRUE |

There were also 7 genera detected that were not in the original mock
community sample, which appear to be contaminants. These made up 0.2805
percent of the mock community controls and a total of 296 reads. These
are shown in the table below.

**Supplementary Table 21: The total raw and relative abundance of
genera-level contaminants detected in mock community samples and the
number of samples that contained the genera detected**

| Genus | Total raw abundance | Total relative abundance (%) | Number of samples | Detected in canine samples |
| --- | --- | --- | --- | --- |
| Prevotella 9 | 183 | 0.173 | 3 | TRUE |
| Fusobacterium | 38 | 0.036 | 2 | TRUE |
| Faecalibacterium | 28 | 0.027 | 2 | TRUE |
| Alloprevotella | 26 | 0.025 | 2 | TRUE |
| Prevotellaceae Ga6A1 group | 9 | 0.009 | 1 | TRUE |
| Megamonas | 8 | 0.008 | 2 | TRUE |
| Sutterella | 4 | 0.004 | 1 | TRUE |

The taxonomic classifications at Genus and Phylum level of the three
mock community samples are shown in the figures below.

**Supplementary Figure 11: Raw abundance of Genera in mock
community samples**

**Supplementary Figure 12: Raw abundance of Phyla in mock
community samples**

### 7.4.2 Expected VS actual relative abundances in mock community samples

Underrepresented and overrepresented Genera and Phyla are shown in
the respective Tables below.

**Supplementary Table 22: The expected and actual relative
abundance of genera in mock community samples and the percentage
difference between them**

| Genus | Expected relative abundance | Total raw abundance | Total relative abundance (%) | Difference to expected (%) |
| --- | --- | --- | --- | --- |
| Clostridium sensu stricto 1 | 5 | 15239 | 14.442 | 188.834 |
| Bacillus | 5 | 9742 | 9.232 | 84.646 |
| Helicobacter | 5 | 1627 | 1.542 | -69.163 |
| Bacteroides | 5 | 7880 | 7.468 | 49.354 |
| Actinomyces | 5 | 2729 | 2.586 | -48.276 |
| Cutibacterium | 5 | 2902 | 2.750 | -44.997 |
| Neisseria | 5 | 2930 | 2.777 | -44.466 |
| Enterococcus | 5 | 3219 | 3.051 | -38.988 |
| Pseudomonas | 5 | 3293 | 3.121 | -37.586 |
| Deinococcus | 5 | 3444 | 3.264 | -34.724 |
| Rhodobacter | 5 | 3573 | 3.386 | -32.279 |
| Acinetobacter | 5 | 4124 | 3.908 | -21.835 |
| Streptococcus | 10 | 12428 | 11.778 | 17.778 |
| Escherichia-Shigella | 5 | 5950 | 5.639 | 12.774 |
| Porphyromonas | 5 | 5868 | 5.561 | 11.220 |
| Staphylococcus | 10 | 9841 | 9.326 | -6.739 |
| Bifidobacterium | 5 | 5021 | 4.758 | -4.834 |
| Lactobacillus | 5 | 5415 | 5.132 | 2.634 |
| Prevotella 9 | NA | 183 | 0.173 | NA |
| Fusobacterium | NA | 38 | 0.036 | NA |
| Faecalibacterium | NA | 28 | 0.027 | NA |
| Alloprevotella | NA | 26 | 0.025 | NA |
| Prevotellaceae Ga6A1 group | NA | 9 | 0.009 | NA |
| Megamonas | NA | 8 | 0.008 | NA |
| Sutterella | NA | 4 | 0.004 | NA |

**Supplementary Table 23: The expected and actual relative
abundance of phyla in mock community samples and the percentage
difference between them**

| Phylum | Expected relative abundance | Total raw abundance | Total relative abundance (%) | Difference to expected (%) |
| --- | --- | --- | --- | --- |
| Epsilonbacteraeota | 5 | 1627 | 1.542 | -69.16 |
| Deinococcus-Thermus | 5 | 3444 | 3.264 | -34.72 |
| Actinobacteria | 15 | 10652 | 10.095 | -32.70 |
| Firmicutes | 40 | 55920 | 52.994 | 32.48 |
| Bacteroidetes | 10 | 13966 | 13.235 | 32.35 |
| Proteobacteria | 25 | 19874 | 18.834 | -24.66 |

# 8 Analysis of laboratory batch effects

## 8.1 Comparison of sample storage batch

**Supplementary Table 24: Number of samples per sample storage
batch**

| Number of samples | Number of batches |
| --- | --- |
| 1 | 5 |
| 2 | 7 |
| 3 | 9 |
| 4 | 7 |
| 5 | 9 |
| 6 | 6 |
| 8 | 2 |
| 9 | 4 |
| 10 | 2 |
| 15 | 1 |

It was challenging to investigate the number of samples per batch
further, as some batches of storage only had very small numbers in.
Also, as the study progressed there were more likely to be older dogs in
each storage sample, so any results are likely to be confounded by age
of dogs, antibiotic exposure etc. Therefore, it was decided not to
included this in analysis.

## 8.2 Comparison of DNA extraction batch

**Supplementary Table 25: Number of samples per DNA extraction
batch**

| Number of samples | Number of DNA extraction batches |
| --- | --- |
| 3 | 1 |
| 22 | 1 |
| 23 | 1 |
| 24 | 7 |
| 26 | 1 |

### 8.2.1 Comparison of samples and controls by number of reads

**Supplementary Figure 13: Boxplot with mean (black diamond) of
reads by DNA extraction batch**

**Supplementary Table 26: Summary statistics of number of reads in
different DNA extraction batches**

| Wave | Min | Max | Median | Mean | Lower CI | Upper CI |
| --- | --- | --- | --- | --- | --- | --- |
| 1 | 249 | 80802 | 60162 | 57155 | 50649 | 63661 |
| 2 | 295 | 89637 | 63064 | 59429 | 50164 | 68694 |
| 3 | 122 | 67777 | 54020 | 48434 | 41138 | 55730 |
| 4 | 197 | 63039 | 43467 | 40675 | 34273 | 47076 |
| 5 | 244 | 96197 | 57006 | 60741 | 52662 | 68820 |
| 6 | 280 | 74552 | 52336 | 49762 | 43959 | 55566 |
| 7 | 62 | 69614 | 51359 | 46118 | 37592 | 54643 |
| 8 | 188 | 62941 | 45496 | 43912 | 39035 | 48788 |
| 9 | 130 | 68202 | 50136 | 49826 | 43838 | 55815 |
| 10 | 57 | 64929 | 47497 | 45982 | 40193 | 51771 |
| NA | 30312 | 42308 | 32901 | 35174 | 19492 | 50855 |

### 8.2.2 Comparison of DNA extraction batch by alpha diversity

**Supplementary Figure 14: Alpha diversity in different DNA
extraction batches**

**Supplementary Table 27: Summary statistics of Richness in
different DNA extraction batches**

| Wave | Min | Max | Median | Mean | Lower CI | Upper CI |
| --- | --- | --- | --- | --- | --- | --- |
| 1 | 10 | 182 | 113.5 | 116.04 | 100.35 | 131.73 |
| 2 | 14 | 186 | 101.0 | 99.88 | 82.01 | 117.74 |
| 3 | 5 | 157 | 115.5 | 111.00 | 95.71 | 126.29 |
| 4 | 11 | 180 | 95.0 | 95.74 | 79.70 | 111.78 |
| 5 | 13 | 176 | 92.5 | 98.17 | 83.96 | 112.38 |
| 6 | 14 | 170 | 97.5 | 98.88 | 86.87 | 110.88 |
| 7 | 4 | 163 | 98.0 | 90.35 | 73.64 | 107.05 |
| 8 | 17 | 175 | 94.0 | 100.17 | 85.52 | 114.81 |
| 9 | 10 | 164 | 104.5 | 100.64 | 84.31 | 116.96 |
| 10 | 7 | 163 | 101.0 | 98.29 | 86.08 | 110.50 |
| NA | 27 | 32 | 29.0 | 29.33 | 23.08 | 35.59 |

**Supplementary Table 28: Summary statistics of number of Shannon
diversity in different DNA extraction batches**

| Wave | Min | Max | Median | Mean | Lower CI | Upper CI |
| --- | --- | --- | --- | --- | --- | --- |
| 1 | 1.771 | 3.911 | 3.537 | 3.412 | 3.195 | 3.629 |
| 2 | 1.794 | 3.871 | 3.399 | 3.166 | 2.923 | 3.409 |
| 3 | 1.215 | 3.815 | 3.520 | 3.341 | 3.098 | 3.583 |
| 4 | 2.117 | 3.973 | 3.301 | 3.281 | 3.095 | 3.467 |
| 5 | 2.191 | 3.797 | 3.346 | 3.230 | 3.051 | 3.409 |
| 6 | 2.030 | 3.986 | 3.253 | 3.213 | 3.005 | 3.421 |
| 7 | 1.091 | 3.685 | 3.366 | 3.113 | 2.856 | 3.370 |
| 8 | 2.438 | 3.996 | 3.381 | 3.389 | 3.242 | 3.537 |
| 9 | 1.847 | 3.876 | 3.411 | 3.236 | 2.993 | 3.478 |
| 10 | 1.641 | 4.055 | 3.340 | 3.267 | 3.059 | 3.475 |
| NA | 2.941 | 2.968 | 2.945 | 2.951 | 2.914 | 2.988 |

**Supplementary Table 29: Summary statistics of number of Faith PD
in different DNA extraction batches**

| Wave | Min | Max | Median | Mean | Lower CI | Upper CI |
| --- | --- | --- | --- | --- | --- | --- |
| 1 | 2.996 | 10.707 | 8.042 | 8.029 | 7.319 | 8.739 |
| 2 | 3.312 | 10.938 | 7.161 | 7.313 | 6.541 | 8.086 |
| 3 | 2.506 | 10.014 | 8.082 | 7.900 | 7.173 | 8.627 |
| 4 | 3.060 | 10.368 | 6.882 | 6.947 | 6.269 | 7.625 |
| 5 | 3.326 | 10.403 | 7.162 | 7.079 | 6.440 | 7.717 |
| 6 | 3.248 | 10.105 | 6.808 | 7.247 | 6.636 | 7.859 |
| 7 | 2.437 | 11.651 | 7.193 | 7.013 | 6.132 | 7.894 |
| 8 | 3.723 | 10.151 | 6.967 | 7.174 | 6.492 | 7.857 |
| 9 | 2.798 | 10.755 | 7.160 | 7.379 | 6.556 | 8.203 |
| 10 | 2.791 | 10.375 | 7.039 | 7.269 | 6.638 | 7.899 |
| NA | 4.415 | 5.031 | 4.890 | 4.779 | 3.976 | 5.581 |

### 8.2.3 Comparison of DNA extraction batches by beta diversity

**Supplementary Figure 15: PCoA using beta diversity metrics of
samples by DNA extraction batch (raw abundances)**

**Supplementary Figure 16: PCoA using beta diversity metrics of
samples by DNA extraction batch (relative abundances)**

There are no obvious trends or outliers in reads, alpha or beta
diversity by DNA extraction batch based on these visualisations.

## 8.3 Comparison of PCR plates

**Supplementary Table 30: Number of samples per PCR plate**

| Number of samples | Number of PCR plates |
| --- | --- |
| 52 | 1 |
| 95 | 2 |

### 8.3.1 Comparison of samples and controls by number of reads

**Supplementary Figure 17: Boxplot with mean (black diamond) of
reads by DNA extraction batch**

**Supplementary Table 31: Summary statistics of number of reads in
different PCR plates**

| Wave | Min | Max | Median | Mean | Lower CI | Upper CI |
| --- | --- | --- | --- | --- | --- | --- |
| Plate1 | 122 | 89637 | 54473 | 51972 | 48277 | 55667 |
| Plate2 | 163 | 96197 | 51674 | 50807 | 47411 | 54203 |
| Plate3 | 57 | 68202 | 48607 | 45043 | 40239 | 49848 |

### 8.3.2 Comparison of PCR plate by alpha diversity

**Supplementary Figure 18: Alpha diversity in different PCR
plates**

**Supplementary Table 32: Summary statistics of Richness in
different PCR plates**

| Wave | Min | Max | Median | Mean | Lower CI | Upper CI |
| --- | --- | --- | --- | --- | --- | --- |
| Plate1 | 5 | 186 | 108 | 105.81 | 97.97 | 113.6 |
| Plate2 | 9 | 176 | 96 | 96.28 | 89.82 | 102.7 |
| Plate3 | 4 | 175 | 101 | 96.00 | 84.69 | 107.3 |

**Supplementary Table 33: Summary statistics of number of Shannon
diversity in different PCR plates**

| Wave | Min | Max | Median | Mean | Lower CI | Upper CI |
| --- | --- | --- | --- | --- | --- | --- |
| Plate1 | 1.215 | 3.973 | 3.465 | 3.301 | 3.194 | 3.408 |
| Plate2 | 1.671 | 3.996 | 3.339 | 3.247 | 3.158 | 3.335 |
| Plate3 | 1.091 | 4.055 | 3.340 | 3.209 | 3.044 | 3.374 |

**Supplementary Table 34: Summary statistics of number of Faith PD
in different PCR plates**

| Wave | Min | Max | Median | Mean | Lower CI | Upper CI |
| --- | --- | --- | --- | --- | --- | --- |
| Plate1 | 2.506 | 10.94 | 7.652 | 7.558 | 7.206 | 7.909 |
| Plate2 | 2.841 | 11.65 | 7.075 | 7.127 | 6.805 | 7.449 |
| Plate3 | 2.437 | 10.76 | 7.047 | 7.155 | 6.617 | 7.692 |

### 8.3.3 Comparison of PCR platees by beta diversity

**Supplementary Figure 19: PCoA using beta diversity metrics of
samples by PCR plate (raw abundances)**

**Supplementary Figure 20: PCoA using beta diversity metrics of
samples by PCR plate (relative abundances)**

The number of reads in Plate 3 seems to be slightly decreased in
comparison with the other plates. However, this can be explained by the
fact there are more negative controls proportionally. None of the
differences were significant (i.e., all confidence intervals
overlapped), so it was considered reasonable to discount PCR plate from
further analyses.

# 9 Contaminant detection and removal

## 9.1 Mitochondria and cloroplast removal

```
## phyloseq-class experiment-level object
## otu_table()   OTU Table:         [ 1792 taxa and 242 samples ]
## sample_data() Sample Data:       [ 242 samples by 61 sample variables ]
## tax_table()   Taxonomy Table:    [ 1792 taxa by 7 taxonomic ranks ]
## phy_tree()    Phylogenetic Tree: [ 1792 tips and 1791 internal nodes ]
```

Removing chloroplasts and mitochondria reduced the number of taxa in
the data by 22, from 1814 to 1792.

## 9.2 Removal of other contaminants using decontam

```
## phyloseq-class experiment-level object
## otu_table()   OTU Table:         [ 1788 taxa and 242 samples ]
## sample_data() Sample Data:       [ 242 samples by 62 sample variables ]
## tax_table()   Taxonomy Table:    [ 1788 taxa by 7 taxonomic ranks ]
## phy_tree()    Phylogenetic Tree: [ 1788 tips and 1787 internal nodes ]
```

There were 4 contaminants identified. The maximum number of samples
that any of these contaminants were found in was 17. The percentage of
all taxa that these contaminants contributed was 0.034%. They were
removed from remaining analyses, which reduced the number of taxa in the
data by 4, from 1792 to 1788.

# 10 Normalisation of samples and filtering data

## 10.1 Normalisation of samples by SRS (alternative to rarefraction)

First the cutoff for SRS is determined, based on the number of
reads/sample.

| Number of samples | Number of PCR plates |
| --- | --- |
| 52 | 1 |
| 95 | 2 |

**Supplementary Table 35: Number of reads in samples with lowest
20 read counts**

| Sample type | Number of reads |
| --- | --- |
| negative control | 57 |
| negative control | 62 |
| negative control | 122 |
| negative control | 130 |
| negative control | 163 |
| negative control | 188 |
| negative control | 190 |
| negative control | 197 |
| negative control | 244 |
| negative control | 247 |
| negative control | 249 |
| negative control | 280 |
| negative control | 295 |
| negative control | 457 |
| negative control | 900 |
| canine faeces | 4659 |
| canine faeces | 29454 |
| canine faeces | 29555 |
| mock community | 30312 |
| canine faeces | 30469 |

Based on this, the cutoff of 29454 would result in only one canine
sample being lost and a very high (>25000) readcount for remaining
samples.

This reduced the number of samples in the data by 16, from 242 to
226. This reduced the number of taxa in the data by 23, from 1788 to
1765.

## 10.2 Remove unneccessary samples (mock community samples, repeats, missing questionnaire dogs)

This reduced the number of samples in the data by 13, from 226 to
213. This reduced the number of taxa in the data by 34, from 1765 to
1731.

## 10.3 Summary of questionnaire data

### 10.3.1 Household waste

**Supplementary Table 36: Household waste score by wave of
collection**

| Wave | Median | Mean | Lower CI | Upper CI |
| --- | --- | --- | --- | --- |
| Wave 1 | 1 | 1.066 | 0.769 | 1.363 |
| Wave 2 | 1 | 1.099 | 0.824 | 1.373 |
| Wave 3 | 1 | 0.985 | 0.739 | 1.231 |

### 10.3.2 Outdoors waste

**Supplementary Table 37: Outdoors waste score by wave of
collection**

| Wave | Median | Mean | Lower CI | Upper CI |
| --- | --- | --- | --- | --- |
| Wave 1 | 3 | 2.368 | 2.048 | 2.689 |
| Wave 2 | 3 | 2.648 | 2.340 | 2.955 |
| Wave 3 | 3 | 2.515 | 2.213 | 2.818 |

### 10.3.3 Coprophagia

**Supplementary Table 38: Coprophagia score by wave of
collection**

| Wave | Median | Mean | Lower CI | Upper CI |
| --- | --- | --- | --- | --- |
| Wave 1 | 0 | 0.776 | 0.522 | 1.031 |
| Wave 2 | 1 | 1.183 | 0.897 | 1.470 |
| Wave 3 | 1 | 1.015 | 0.741 | 1.290 |

### 10.3.4 Stress

**Supplementary Table 39: Stress score by wave of
collection**

| Wave | Median | Mean | Lower CI | Upper CI |
| --- | --- | --- | --- | --- |
| Wave 1 | 1 | 1.263 | 1.162 | 1.364 |
| Wave 2 | 1 | 1.310 | 1.193 | 1.427 |
| Wave 3 | 1 | 1.379 | 1.244 | 1.514 |

### 10.3.5 Contact with dogs

**Supplementary Table 40: Contact with dogs score by wave of
collection**

| Wave | Median | Mean | Lower CI | Upper CI |
| --- | --- | --- | --- | --- |
| Wave 1 | 4 | 3.539 | 3.337 | 3.742 |
| Wave 2 | 4 | 3.648 | 3.469 | 3.827 |
| Wave 3 | 4 | 3.682 | 3.498 | 3.866 |

### 10.3.6 Contact with cats

**Supplementary Table 41: Contact with cats score by wave of
collection**

| Wave | Median | Mean | Lower CI | Upper CI |
| --- | --- | --- | --- | --- |
| Wave 1 | 1 | 1.645 | 1.257 | 2.032 |
| Wave 2 | 1 | 1.732 | 1.332 | 2.132 |
| Wave 3 | 1 | 1.773 | 1.351 | 2.195 |

### 10.3.7 Contact with horses

**Supplementary Table 42: Contact with horses score by wave of
collection**

| Wave | Median | Mean | Lower CI | Upper CI |
| --- | --- | --- | --- | --- |
| Wave 1 | 0 | 0.487 | 0.258 | 0.715 |
| Wave 2 | 0 | 0.704 | 0.450 | 0.959 |
| Wave 3 | 1 | 0.970 | 0.701 | 1.239 |

### 10.3.8 Contact with Farm animals

**Supplementary Table 43: Contact with farm animals score by wave
of collection**

| Wave | Median | Mean | Lower CI | Upper CI |
| --- | --- | --- | --- | --- |
| Wave 1 | 0 | 0.684 | 0.400 | 0.969 |
| Wave 2 | 1 | 0.901 | 0.610 | 1.193 |
| Wave 3 | 1 | 1.030 | 0.741 | 1.319 |

### 10.3.9 Contact with Chickens

**Supplementary Table 44: Contact with chickens score by wave of
collection**

| Wave | Median | Mean | Lower CI | Upper CI |
| --- | --- | --- | --- | --- |
| Wave 1 | 0 | 0.408 | 0.163 | 0.653 |
| Wave 2 | 0 | 0.437 | 0.184 | 0.689 |
| Wave 3 | 0 | 0.652 | 0.348 | 0.955 |

### 10.3.10 Antibiotics

**Supplementary Table 45: Time since antibiotics by wave of
collection**

| Antibiotics | Wave | Number | Percentage |
| --- | --- | --- | --- |
| None | Wave 1 | 60 | 41.10 |
| None | Wave 2 | 49 | 33.56 |
| None | Wave 3 | 37 | 25.34 |
| Under 1 week | Wave 1 | 3 | 33.33 |
| Under 1 week | Wave 2 | 4 | 44.44 |
| Under 1 week | Wave 3 | 2 | 22.22 |
| 1 to 4 weeks | Wave 1 | 5 | 62.50 |
| 1 to 4 weeks | Wave 2 | 2 | 25.00 |
| 1 to 4 weeks | Wave 3 | 1 | 12.50 |
| 4 to 8 weeks | Wave 1 | 7 | 58.33 |
| 4 to 8 weeks | Wave 2 | 3 | 25.00 |
| 4 to 8 weeks | Wave 3 | 2 | 16.67 |
| 8 to 16 weeks | Wave 1 | 1 | 12.50 |
| 8 to 16 weeks | Wave 2 | 3 | 37.50 |
| 8 to 16 weeks | Wave 3 | 4 | 50.00 |
| Over 16 weeks | Wave 2 | 10 | 33.33 |
| Over 16 weeks | Wave 3 | 20 | 66.67 |

**Supplementary Table 46: Prevalence of antibiotics by wave of
collection**

| Had antibiotics | Wave | Number | Prevalence | Prevalence lower CI | Prevalence upper CI |
| --- | --- | --- | --- | --- | --- |
| Wave 1 | FALSE | 60 | 0.789 | 0.698 | 0.881 |
| Wave 1 | TRUE | 16 | 0.211 | 0.119 | 0.302 |
| Wave 2 | FALSE | 49 | 0.690 | 0.583 | 0.798 |
| Wave 2 | TRUE | 22 | 0.310 | 0.202 | 0.417 |
| Wave 3 | FALSE | 37 | 0.561 | 0.441 | 0.680 |
| Wave 3 | TRUE | 29 | 0.439 | 0.320 | 0.559 |

### 10.3.11 Vomiting

**Supplementary Table 47: Time since vomiting by wave of
collection**

| Vomiting | Wave | Number | Percentage |
| --- | --- | --- | --- |
| Never | Wave 1 | 71 | 41.765 |
| Never | Wave 2 | 54 | 31.765 |
| Never | Wave 3 | 45 | 26.471 |
| Within 4 weeks | Wave 1 | 3 | 27.273 |
| Within 4 weeks | Wave 2 | 7 | 63.636 |
| Within 4 weeks | Wave 3 | 1 | 9.091 |
| 4 to 8 weeks | Wave 1 | 2 | 22.222 |
| 4 to 8 weeks | Wave 2 | 5 | 55.556 |
| 4 to 8 weeks | Wave 3 | 2 | 22.222 |
| 8 to 16 weeks | Wave 2 | 2 | 66.667 |
| 8 to 16 weeks | Wave 3 | 1 | 33.333 |
| Over 16 weeks | Wave 2 | 3 | 15.000 |
| Over 16 weeks | Wave 3 | 17 | 85.000 |

**Supplementary Table 48: Prevalence of vomiting by wave of
collection**

| Vomiting | Wave | Number | Prevalence | Prevalence lower CI | Prevalence upper CI |
| --- | --- | --- | --- | --- | --- |
| Wave 1 | FALSE | 71 | 0.934 | 0.878 | 0.990 |
| Wave 1 | TRUE | 5 | 0.066 | 0.010 | 0.122 |
| Wave 2 | FALSE | 54 | 0.761 | 0.661 | 0.860 |
| Wave 2 | TRUE | 17 | 0.239 | 0.140 | 0.339 |
| Wave 3 | FALSE | 45 | 0.682 | 0.569 | 0.794 |
| Wave 3 | TRUE | 21 | 0.318 | 0.206 | 0.431 |

### 10.3.12 Diarrhoea

**Supplementary Table 49: Time since diarrhoea by wave of
collection**

| Vomiting | Wave | Number | Percentage |
| --- | --- | --- | --- |
| Never | Wave 1 | 53 | 44.915 |
| Never | Wave 2 | 36 | 30.508 |
| Never | Wave 3 | 29 | 24.576 |
| Within 4 weeks | Wave 1 | 11 | 47.826 |
| Within 4 weeks | Wave 2 | 10 | 43.478 |
| Within 4 weeks | Wave 3 | 2 | 8.696 |
| 4 to 8 weeks | Wave 1 | 8 | 57.143 |
| 4 to 8 weeks | Wave 2 | 5 | 35.714 |
| 4 to 8 weeks | Wave 3 | 1 | 7.143 |
| 8 to 16 weeks | Wave 1 | 2 | 13.333 |
| 8 to 16 weeks | Wave 2 | 8 | 53.333 |
| 8 to 16 weeks | Wave 3 | 5 | 33.333 |
| Over 16 weeks | Wave 1 | 2 | 4.651 |
| Over 16 weeks | Wave 2 | 12 | 27.907 |
| Over 16 weeks | Wave 3 | 29 | 67.442 |

**Supplementary Table 50: Prevalence of diarrhoea by wave of
collection**

| Diarrhoea | Wave | Number | Prevalence | Prevalence lower CI | Prevalence upper CI |
| --- | --- | --- | --- | --- | --- |
| Wave 1 | FALSE | 53 | 0.697 | 0.594 | 0.801 |
| Wave 1 | TRUE | 23 | 0.303 | 0.199 | 0.406 |
| Wave 2 | FALSE | 36 | 0.507 | 0.391 | 0.623 |
| Wave 2 | TRUE | 35 | 0.493 | 0.377 | 0.609 |
| Wave 3 | FALSE | 29 | 0.439 | 0.320 | 0.559 |
| Wave 3 | TRUE | 37 | 0.561 | 0.441 | 0.680 |

# 11 Statistical analysis

## 11.1 Alpha diversity

### 11.1.1 Richness (observed number of ASVs)

**Supplementary Figure 21: Distribution of richness in canine
samples**

**Supplementary Figure 22: Quantile-quantile plot (a) and
histogram of model residuals (b) for assessing model to predict richness
in canine samples**

**Supplementary Table 51: Performance metrics of model to predict
richness in canine samples**

| AIC | AICc | BIC | R2\_conditional | R2\_marginal | ICC | RMSE | Sigma |
| --- | --- | --- | --- | --- | --- | --- | --- |
| 1813 | 1832 | 1948 | 0.521 | 0.293 | 0.322 | 17.11 | 20.78 |

### 11.1.2 Phylogentic diversity (Faith PD)

**Supplementary Figure 23: Distribution of faith PD in canine
samples**

**Supplementary Figure 24: Quantile-quantile plot (a) and
histogram of model residuals (b) for assessing model to predict faith PD
in canine samples**

**Supplementary Table 52: Performance metrics of model to predict
faith PD in canine samples**

| AIC | AICc | BIC | R2\_conditional | R2\_marginal | ICC | RMSE | Sigma |
| --- | --- | --- | --- | --- | --- | --- | --- |
| 723 | 742.1 | 857.5 | 0.531 | 0.331 | 0.299 | 0.77 | 0.93 |

### 11.1.3 Shannon diversity

**Supplementary Figure 25: Distribution of Shannon diversity in
canine samples**

**Supplementary Figure 26: Quantile-quantile plot (a) and
histogram of model residuals (b) for assessing model to predict Shannon
diversity in canine samples**

**Supplementary Table 53: Performance metrics of model to predict
Shannon diversity in canine samples**

| AIC | AICc | BIC | R2\_conditional | R2\_marginal | ICC | RMSE | Sigma |
| --- | --- | --- | --- | --- | --- | --- | --- |
| 332.4 | 351.5 | 466.9 | 0.365 | 0.263 | 0.138 | 0.28 | 0.324 |

### 11.1.4 Simpson diversity

**Supplementary Figure 27: Distribution of Simpson diversity in
canine samples**

**Supplementary Figure 28: Quantile-quantile plot (a) and
histogram of model residuals (b) for assessing model to predict Simpson
diversity in canine samples**

**Supplementary Table 54: Performance metrics of model to predict
Simpson diversity in canine samples**

| AIC | AICc | BIC | R2\_conditional | R2\_marginal | ICC | RMSE | Sigma |
| --- | --- | --- | --- | --- | --- | --- | --- |
| 1314 | 1333 | 1449 | 0.32 | 0.154 | 0.196 | 4.457 | 5.247 |

### 11.1.5 Combined results

**Supplementary Table 55: Combined results for models predicting
alpha diversity in canine samples**

| Variable | Richness coefficient | Richness P-value | Faith PD coefficient | Faith PD P-value | Shannon index coeffiecient | Shannon index P-value | Simpson index coeffiecient | Simpson index P-value |
| --- | --- | --- | --- | --- | --- | --- | --- | --- |
| (Intercept) | 97.468 | 0.000 | 6.655 | 0.000 | 3.241 | 0.000 | 14.216 | 0.000 |
| waveWave 2 | -4.681 | 0.235 | -0.179 | 0.310 | 0.027 | 0.648 | 1.559 | 0.113 |
| waveWave 3 | -10.605 | 0.024 | -0.447 | 0.032 | 0.001 | 0.989 | 1.230 | 0.283 |
| sexMale | -0.532 | 0.916 | -0.110 | 0.617 | 0.034 | 0.591 | 0.940 | 0.396 |
| colourchocolate | 13.234 | 0.126 | 0.267 | 0.476 | 0.038 | 0.727 | 0.030 | 0.987 |
| colourfox red | -6.845 | 0.345 | -0.343 | 0.279 | -0.128 | 0.160 | -1.098 | 0.485 |
| colouryellow | -2.311 | 0.710 | -0.025 | 0.926 | -0.046 | 0.551 | -0.224 | 0.868 |
| household\_typeMore than one Adult | -2.586 | 0.670 | -0.120 | 0.650 | 0.025 | 0.743 | -0.121 | 0.926 |
| household\_typeRetired (Single or Couple) | -0.898 | 0.914 | -0.112 | 0.757 | 0.028 | 0.789 | 0.677 | 0.710 |
| household\_typeSingle Adult | -12.935 | 0.126 | -0.495 | 0.177 | -0.046 | 0.657 | 0.065 | 0.971 |
| household\_smoking\_statusYes | -11.072 | 0.339 | -0.244 | 0.627 | -0.007 | 0.962 | 0.155 | 0.951 |
| UK\_regionEngland Midlands and Wales | 0.951 | 0.880 | 0.201 | 0.465 | -0.090 | 0.256 | -1.291 | 0.348 |
| UK\_regionEngland North | -2.466 | 0.759 | -0.006 | 0.986 | -0.128 | 0.206 | -2.068 | 0.240 |
| UK\_regionScotland | -9.610 | 0.236 | -0.547 | 0.123 | -0.146 | 0.154 | -1.908 | 0.281 |
| area\_classsuburban | -3.300 | 0.666 | -0.344 | 0.304 | 0.128 | 0.191 | 1.241 | 0.459 |
| area\_classurban | -5.528 | 0.407 | -0.242 | 0.405 | 0.035 | 0.681 | 0.601 | 0.681 |
| household\_waste\_score | -2.682 | 0.122 | -0.060 | 0.438 | -0.035 | 0.161 | -0.452 | 0.277 |
| outdoors\_waste\_score | -0.265 | 0.862 | -0.009 | 0.890 | 0.007 | 0.731 | -0.027 | 0.941 |
| coprophagia\_score | 7.592 | 0.000 | 0.421 | 0.000 | 0.080 | 0.003 | 0.909 | 0.039 |
| stress\_levels | 0.758 | 0.853 | -0.069 | 0.703 | -0.012 | 0.832 | 0.155 | 0.873 |
| contact\_dogs\_score | 4.754 | 0.076 | 0.312 | 0.009 | 0.036 | 0.308 | 0.566 | 0.351 |
| contact\_cats\_score | 1.606 | 0.279 | 0.003 | 0.962 | 0.015 | 0.439 | 0.519 | 0.117 |
| contact\_horses\_score | -5.265 | 0.041 | -0.277 | 0.015 | -0.039 | 0.257 | -0.356 | 0.545 |
| contact\_farm\_animals\_score | -0.452 | 0.835 | -0.022 | 0.819 | -0.010 | 0.732 | -0.547 | 0.282 |
| contact\_chickens\_score | 1.620 | 0.504 | 0.171 | 0.111 | 0.016 | 0.632 | -0.070 | 0.899 |
| antibioticsUnder 1 week | -34.179 | 0.000 | -0.950 | 0.018 | -0.667 | 0.000 | -5.346 | 0.015 |
| antibiotics1 to 4 weeks | -3.787 | 0.708 | 0.246 | 0.585 | -0.171 | 0.254 | -1.748 | 0.479 |
| antibiotics4 to 8 weeks | -11.095 | 0.238 | -0.116 | 0.780 | -0.256 | 0.066 | -3.196 | 0.162 |
| antibiotics8 to 16 weeks | -2.528 | 0.791 | 0.059 | 0.890 | -0.182 | 0.197 | -1.822 | 0.433 |
| antibioticsOver 16 weeks | -1.043 | 0.884 | 0.127 | 0.686 | -0.039 | 0.692 | -0.626 | 0.706 |
| vomitingWithin 4 weeks | 7.426 | 0.407 | 0.123 | 0.757 | 0.255 | 0.052 | 1.306 | 0.544 |
| vomiting4 to 8 weeks | 4.181 | 0.677 | -0.086 | 0.847 | -0.006 | 0.966 | -0.820 | 0.737 |
| vomiting8 to 16 weeks | 9.000 | 0.558 | 0.142 | 0.835 | 0.275 | 0.231 | 2.083 | 0.580 |
| vomitingOver 16 weeks | -6.876 | 0.368 | 0.254 | 0.453 | -0.040 | 0.713 | -0.986 | 0.584 |
| diarrhoeaWithin 4 weeks | -1.346 | 0.837 | 0.580 | 0.046 | -0.061 | 0.514 | -0.649 | 0.677 |
| diarrhoea4 to 8 weeks | 12.370 | 0.206 | 0.182 | 0.675 | 0.243 | 0.088 | 2.893 | 0.220 |
| diarrhoea8 to 16 weeks | 3.566 | 0.630 | -0.116 | 0.723 | -0.083 | 0.440 | -1.587 | 0.372 |
| diarrhoeaOver 16 weeks | 9.598 | 0.130 | 0.231 | 0.407 | 0.090 | 0.297 | 0.212 | 0.884 |

### 11.1.6 Boxplot of alpha diversity by wave of sample collection

**Supplementary Figure 29: Boxplots of alpha diversity metrics
in Labrador Retriever puppy faecal samples by wave of sample
collection**

### 11.1.7 Boxplot of alpha diversity by coprophagia score

**Supplementary Figure 30: Boxplots of alpha diversity metrics
in Labrador Retriever puppy faecal samples by coprophagia
score**

### 11.1.8 Boxplot of alpha diversity by contact with dogs

**Supplementary Figure 31: Boxplots of alpha diversity metrics
in Labrador Retriever puppy faecal samples by dog contact
score**

### 11.1.9 Boxplot of alpha diversity by contact with horses

**Supplementary Figure 32: Boxplots of alpha diversity metrics
in Labrador Retriever puppy faecal samples by horse contact
score**

### 11.1.10 Boxplot of alpha diversity by antibiotic treatment

**Supplementary Figure 33: Boxplots of alpha diversity metrics
in Labrador Retriever puppy faecal samples by oral antibiotic
treatment**

### 11.1.11 Boxplot of alpha diversity by diarrhoea

**Supplementary Figure 34: Boxplots of alpha diversity metrics
in Labrador Retriever puppy faecal samples by the time since
diarrhoea**

## 11.2 Beta diversity

### 11.2.1 PERMANOVA

**Supplementary Table 56: Combined results for PERMANOVA analysis
of beta diversity in canine samples, using only individual dog ID to
explain variance**

| Variable | Jaccard R^2 | Jaccard P-value | Bray-curtis R^2 | Bray-curtis P-value | Unweighted unifrac R^2 | Unweighted unifrac P-value | Weighted unifrac R^2 | Weighted unifrac P-value |
| --- | --- | --- | --- | --- | --- | --- | --- | --- |
| dog\_ID | 0.444 | 0.001 | 0.521 | 0.001 | 0.507 | 0.001 | 0.496 | 0.001 |
| Residual | 0.556 | NA | 0.479 | NA | 0.493 | NA | 0.504 | NA |
| Total | 1.000 | NA | 1.000 | NA | 1.000 | NA | 1.000 | NA |
| R2\_explained | 0.444 | NA | 0.521 | NA | 0.507 | NA | 0.496 | NA |

**Supplementary Table 57: Combined results for PERMANOVA analysis
of beta diversity in canine samples, after removing effect of dog
ID**

| Variable | Jaccard R^2 | Jaccard P-value | Bray-curtis R^2 | Bray-curtis P-value | Unweighted unifrac R^2 | Unweighted unifrac P-value | Weighted unifrac R^2 | Weighted unifrac P-value |
| --- | --- | --- | --- | --- | --- | --- | --- | --- |
| wave | 0.013 | 0.001 | 0.016 | 0.002 | 0.019 | 0.001 | 0.015 | 0.031 |
| sex | 0.005 | 0.818 | 0.005 | 0.637 | 0.005 | 0.012 | 0.008 | 0.132 |
| colour | 0.017 | 0.862 | 0.020 | 0.677 | 0.022 | 0.031 | 0.022 | 0.685 |
| household\_type | 0.016 | 0.158 | 0.016 | 0.392 | 0.021 | 0.181 | 0.013 | 0.606 |
| household\_smoking\_status | 0.006 | 0.700 | 0.007 | 0.431 | 0.006 | 0.898 | 0.006 | 0.191 |
| UK\_region | 0.016 | 0.608 | 0.015 | 0.714 | 0.022 | 0.214 | 0.009 | 0.888 |
| area\_class | 0.011 | 0.889 | 0.014 | 0.751 | 0.010 | 0.827 | 0.013 | 0.666 |
| household\_waste\_score | 0.004 | 0.831 | 0.003 | 0.803 | 0.008 | 0.002 | 0.005 | 0.099 |
| outdoors\_waste\_score | 0.004 | 0.967 | 0.002 | 0.917 | 0.005 | 0.128 | 0.004 | 0.342 |
| coprophagia\_score | 0.006 | 0.097 | 0.008 | 0.121 | 0.026 | 0.005 | 0.007 | 0.168 |
| stress\_levels | 0.005 | 0.471 | 0.006 | 0.292 | 0.004 | 0.538 | 0.005 | 0.637 |
| contact\_dogs\_score | 0.006 | 0.049 | 0.008 | 0.034 | 0.010 | 0.023 | 0.011 | 0.062 |
| contact\_cats\_score | 0.005 | 0.278 | 0.004 | 0.467 | 0.008 | 0.118 | 0.002 | 0.701 |
| contact\_horses\_score | 0.005 | 0.337 | 0.006 | 0.342 | 0.012 | 0.293 | 0.003 | 0.563 |
| contact\_farm\_animals\_score | 0.005 | 0.128 | 0.005 | 0.119 | 0.005 | 0.833 | 0.004 | 0.517 |
| contact\_chickens\_score | 0.005 | 0.581 | 0.004 | 0.639 | 0.004 | 0.535 | 0.001 | 0.638 |
| antibiotics | 0.029 | 0.022 | 0.039 | 0.013 | 0.040 | 0.007 | 0.039 | 0.115 |
| vomiting | 0.018 | 0.534 | 0.018 | 0.480 | 0.018 | 0.563 | 0.011 | 0.878 |
| diarrhoea | 0.019 | 0.392 | 0.020 | 0.419 | 0.019 | 0.169 | 0.015 | 0.759 |
| Residual | 0.793 | NA | 0.761 | NA | 0.728 | NA | 0.783 | NA |
| Total | 1.000 | NA | 1.000 | NA | 1.000 | NA | 1.000 | NA |
| R2\_explained | 0.207 | NA | 0.239 | NA | 0.272 | NA | 0.217 | NA |

### 11.2.2 PERMDISP

**Supplementary Table 58: Combined results for PERMDISP analysis
of beta diversity in canine samples**

| Variable | Jaccard FDR | Bray-curtis FDR | Unweighted unifrac FDR | Weighted unifrac FDR |
| --- | --- | --- | --- | --- |
| wave | 0.002 | 0.004 | 0.012 | 0.021 |
| sex | 0.557 | 0.454 | 0.229 | 0.728 |
| colour | 0.612 | 0.628 | 0.304 | 0.767 |
| household\_type | 0.596 | 0.628 | 0.891 | 0.767 |
| household\_smoking\_status | 0.009 | 0.225 | 0.891 | 0.767 |
| UK\_region | 0.612 | 0.628 | 0.673 | 0.767 |
| area\_class | 0.612 | 0.720 | 0.229 | 0.767 |
| antibiotics | 0.612 | 0.225 | 0.145 | 0.464 |
| vomiting | 0.002 | 0.387 | 0.229 | 0.716 |
| diarrhoea | 0.197 | 0.225 | 0.012 | 0.464 |

### 11.2.3 PCoA of beta diversity by wave of sample collection

**Supplementary Figure 35: PCoA for beta diversity distances in
Labrador Retriever puppy faecal samples by wave of sample
collection**

### 11.2.4 PCoA of beta diversity by dog sex

**Supplementary Figure 36: PCoA for beta diversity distances in
Labrador Retriever puppy faecal samples by dog sex**

### 11.2.5 PCoA of beta diversity by dog colour

**Supplementary Figure 37: PCoA for beta diversity distances in
Labrador Retriever puppy faecal samples by dog colour**

### 11.2.6 PCoA of beta diversity by household waste score

**Supplementary Figure 38: PCoA for beta diversity distances in
Labrador Retriever puppy faecal samples by household waste
score**

### 11.2.7 PCoA of beta diversity by coprophagia score

**Supplementary Figure 39: PCoA for beta diversity distances in
Labrador Retriever puppy faecal samples by coprophagia
score**

### 11.2.8 PCoA of beta diversity by dog contact score

**Supplementary Figure 40: PCoA for beta diversity distances in
Labrador Retriever puppy faecal samples by dog contact
score**

### 11.2.9 PCoA of beta diversity by antibiotic treatment

**Supplementary Figure 41: PCoA for beta diversity distances in
Labrador Retriever puppy faecal samples by oral antibiotic
treatment**

# 12 Taxonomy of canine samples

## 12.1 Phylum

**Supplementary Table 59: Relative abundance of microbial phyla in
Labrador Retriever puppy faecal samples**

| Phylum Number | Phylum | Number of samples | Abundance (percentage) |
| --- | --- | --- | --- |
| 1 | Bacteroidetes | 213 | 50.857747 |
| 2 | Firmicutes | 213 | 24.554602 |
| 3 | Fusobacteria | 213 | 16.287925 |
| 4 | Proteobacteria | 213 | 6.947623 |
| 5 | Actinobacteria | 209 | 0.679109 |
| 6 | Epsilonbacteraeota | 203 | 0.464576 |
| 7 | Tenericutes | 112 | 0.165946 |
| 8 | Spirochaetes | 51 | 0.015685 |
| 9 | Deferribacteres | 53 | 0.013957 |
| 10 | Cyanobacteria | 9 | 0.007500 |
| 11 | Unclassified | 22 | 0.004207 |
| 12 | Patescibacteria | 8 | 0.000375 |
| 13 | Verrucomicrobia | 4 | 0.000342 |
| 14 | Chloroflexi | 1 | 0.000228 |
| 15 | Acidobacteria | 1 | 0.000065 |
| 16 | Gemmatimonadetes | 1 | 0.000065 |
| 17 | Planctomycetes | 1 | 0.000033 |
| 18 | Nitrospirae | 1 | 0.000016 |

**Supplementary Figure 42: The relative abundance of phyla in
individual Labrador Retriever puppy faecal samples**

**Supplementary Table 60: Relative abundance of microbial phyla in
Labrador Retriever puppy faecal samples, by wave of sample
collection**

| Phylum Number | Phylum | Wave | Number of samples | Abundance (percentage) |
| --- | --- | --- | --- | --- |
| 1 | Bacteroidetes | Wave 1 | 76 | 50.778233 |
| 1 | Bacteroidetes | Wave 2 | 71 | 51.075475 |
| 1 | Bacteroidetes | Wave 3 | 66 | 50.715086 |
| 2 | Firmicutes | Wave 1 | 76 | 24.563018 |
| 2 | Firmicutes | Wave 2 | 71 | 25.013781 |
| 2 | Firmicutes | Wave 3 | 66 | 24.050945 |
| 3 | Fusobacteria | Wave 1 | 76 | 14.776186 |
| 3 | Fusobacteria | Wave 2 | 71 | 15.904121 |
| 3 | Fusobacteria | Wave 3 | 66 | 18.441596 |
| 4 | Proteobacteria | Wave 1 | 76 | 8.183987 |
| 4 | Proteobacteria | Wave 2 | 71 | 6.906080 |
| 4 | Proteobacteria | Wave 3 | 66 | 5.568622 |
| 5 | Actinobacteria | Wave 1 | 76 | 0.874512 |
| 5 | Actinobacteria | Wave 2 | 69 | 0.584119 |
| 5 | Actinobacteria | Wave 3 | 64 | 0.556283 |
| 6 | Epsilonbacteraeota | Wave 1 | 71 | 0.606419 |
| 6 | Epsilonbacteraeota | Wave 2 | 69 | 0.367092 |
| 6 | Epsilonbacteraeota | Wave 3 | 63 | 0.406110 |
| 7 | Tenericutes | Wave 1 | 34 | 0.155455 |
| 7 | Tenericutes | Wave 2 | 38 | 0.111571 |
| 7 | Tenericutes | Wave 3 | 40 | 0.236520 |
| 8 | Spirochaetes | Wave 1 | 21 | 0.028651 |
| 8 | Spirochaetes | Wave 2 | 18 | 0.009929 |
| 8 | Spirochaetes | Wave 3 | 12 | 0.006946 |
| 9 | Deferribacteres | Wave 1 | 21 | 0.022528 |
| 9 | Deferribacteres | Wave 2 | 20 | 0.011397 |
| 9 | Deferribacteres | Wave 3 | 12 | 0.006840 |
| 10 | Cyanobacteria | Wave 1 | 4 | 0.008499 |
| 10 | Cyanobacteria | Wave 2 | 4 | 0.003717 |
| 10 | Cyanobacteria | Wave 3 | 1 | 0.010418 |
| 11 | Unclassified | Wave 1 | 7 | 0.000548 |
| 11 | Unclassified | Wave 2 | 10 | 0.011446 |
| 11 | Unclassified | Wave 3 | 5 | 0.000631 |
| 12 | Patescibacteria | Wave 1 | 1 | 0.000228 |
| 12 | Patescibacteria | Wave 2 | 7 | 0.000880 |
| 12 | Patescibacteria | Wave 3 | 0 | 0.000000 |
| 13 | Verrucomicrobia | Wave 1 | 3 | 0.000594 |
| 13 | Verrucomicrobia | Wave 2 | 1 | 0.000391 |
| 13 | Verrucomicrobia | Wave 3 | 0 | 0.000000 |
| 14 | Chloroflexi | Wave 1 | 1 | 0.000640 |
| 14 | Chloroflexi | Wave 2 | 0 | 0.000000 |
| 14 | Chloroflexi | Wave 3 | 0 | 0.000000 |
| 15 | Acidobacteria | Wave 1 | 1 | 0.000183 |
| 15 | Acidobacteria | Wave 2 | 0 | 0.000000 |
| 15 | Acidobacteria | Wave 3 | 0 | 0.000000 |
| 16 | Gemmatimonadetes | Wave 1 | 1 | 0.000183 |
| 16 | Gemmatimonadetes | Wave 2 | 0 | 0.000000 |
| 16 | Gemmatimonadetes | Wave 3 | 0 | 0.000000 |
| 17 | Planctomycetes | Wave 1 | 1 | 0.000091 |
| 17 | Planctomycetes | Wave 2 | 0 | 0.000000 |
| 17 | Planctomycetes | Wave 3 | 0 | 0.000000 |
| 18 | Nitrospirae | Wave 1 | 1 | 0.000046 |
| 18 | Nitrospirae | Wave 2 | 0 | 0.000000 |
| 18 | Nitrospirae | Wave 3 | 0 | 0.000000 |

**Supplementary Figure 43: The relative abundance of phyla by
wave of sample collection in Labrador Retriever puppy faecal
samples**

## 12.2 Class

**Supplementary Table 61: Relative abundance of microbial class in
Labrador Retriever puppy faecal samples**

| Class Number | Class | Number of samples | Abundance (percentage) |
| --- | --- | --- | --- |
| 1 | Bacteroidia | 213 | 50.857747 |
| 2 | Clostridia | 213 | 16.763767 |
| 3 | Fusobacteriia | 213 | 16.287925 |
| 4 | Gammaproteobacteria | 213 | 6.927096 |
| 5 | Negativicutes | 210 | 3.895597 |
| 6 | Erysipelotrichia | 213 | 3.218445 |
| 7 | Bacilli | 166 | 0.676402 |
| 8 | Coriobacteriia | 207 | 0.569755 |
| 9 | Campylobacteria | 203 | 0.464576 |
| 10 | Mollicutes | 112 | 0.165946 |
| 11 | Actinobacteria | 84 | 0.108538 |
| 12 | Alphaproteobacteria | 25 | 0.019141 |
| 13 | Brachyspirae | 50 | 0.015489 |
| 14 | Deferribacteres | 53 | 0.013957 |
| 15 | Melainabacteria | 8 | 0.007467 |
| 16 | Unclassified | 32 | 0.004647 |
| 17 | Deltaproteobacteria | 15 | 0.001370 |
| 18 | Thermoleophilia | 1 | 0.000685 |
| 19 | Saccharimonadia | 8 | 0.000375 |
| 20 | Verrucomicrobiae | 4 | 0.000342 |
| 21 | Spirochaetia | 2 | 0.000196 |
| 22 | MB-A2-108 | 1 | 0.000098 |
| 23 | Blastocatellia (Subgroup 4) | 1 | 0.000065 |
| 24 | Gitt-GS-136 | 1 | 0.000065 |
| 25 | KD4-96 | 1 | 0.000065 |
| 26 | Gemmatimonadetes | 1 | 0.000065 |
| 27 | Chloroflexia | 1 | 0.000065 |
| 28 | Planctomycetacia | 1 | 0.000033 |
| 29 | Oxyphotobacteria | 1 | 0.000033 |
| 30 | Dehalococcoidia | 1 | 0.000033 |
| 31 | Nitrospira | 1 | 0.000016 |

## 12.3 Order

**Supplementary Table 62: Relative abundance of microbial order in
Labrador Retriever puppy faecal samples**

| Order Number | Order | Number of samples | Abundance (percentage) |
| --- | --- | --- | --- |
| 1 | Bacteroidales | 213 | 50.857682 |
| 2 | Clostridiales | 213 | 16.763735 |
| 3 | Fusobacteriales | 213 | 16.287925 |
| 4 | Betaproteobacteriales | 212 | 4.522711 |
| 5 | Selenomonadales | 210 | 3.895597 |
| 6 | Erysipelotrichales | 213 | 3.218445 |
| 7 | Aeromonadales | 196 | 1.874103 |
| 8 | Lactobacillales | 162 | 0.671054 |
| 9 | Coriobacteriales | 207 | 0.569755 |
| 10 | Enterobacteriales | 158 | 0.528864 |
| 11 | Campylobacterales | 203 | 0.464576 |
| 12 | Anaeroplasmatales | 95 | 0.142337 |
| 13 | Bifidobacteriales | 79 | 0.104380 |
| 14 | Mycoplasmatales | 22 | 0.021995 |
| 15 | Rhodospirillales | 16 | 0.016141 |
| 16 | Brachyspirales | 50 | 0.015489 |
| 17 | Deferribacterales | 53 | 0.013957 |
| 18 | Gastranaerophilales | 8 | 0.007467 |
| 19 | Bacillales | 14 | 0.005348 |
| 20 | Unclassified | 33 | 0.004859 |
| 21 | Rhizobiales | 5 | 0.001777 |
| 22 | Mollicutes RF39 | 9 | 0.001598 |
| 23 | Corynebacteriales | 5 | 0.001288 |
| 24 | Desulfovibrionales | 14 | 0.001190 |
| 25 | Propionibacteriales | 4 | 0.001027 |
| 26 | Pseudomonadales | 8 | 0.000750 |
| 27 | Streptomycetales | 2 | 0.000717 |
| 28 | Micromonosporales | 2 | 0.000522 |
| 29 | Sphingomonadales | 4 | 0.000391 |
| 30 | Micrococcales | 5 | 0.000375 |
| 31 | Saccharimonadales | 8 | 0.000375 |
| 32 | Solirubrobacterales | 1 | 0.000342 |
| 33 | Gaiellales | 1 | 0.000342 |
| 34 | Reyranellales | 1 | 0.000310 |
| 35 | Pasteurellales | 4 | 0.000310 |
| 36 | Paracaedibacterales | 4 | 0.000277 |
| 37 | Verrucomicrobiales | 3 | 0.000179 |
| 38 | Chthoniobacterales | 1 | 0.000163 |
| 39 | Brevinematales | 1 | 0.000130 |
| 40 | Diplorickettsiales | 3 | 0.000130 |
| 41 | Elsterales | 1 | 0.000114 |
| 42 | Frankiales | 1 | 0.000114 |
| 43 | Myxococcales | 1 | 0.000114 |
| 44 | Pseudonocardiales | 1 | 0.000098 |
| 45 | metagenome | 1 | 0.000098 |
| 46 | Blastocatellales | 1 | 0.000065 |
| 47 | uncultured Chloroflexus sp. | 1 | 0.000065 |
| 48 | uncultured Chloroflexi bacterium | 1 | 0.000065 |
| 49 | Gemmatimonadales | 1 | 0.000065 |
| 50 | Spirochaetales | 1 | 0.000065 |
| 51 | Gammaproteobacteria Incertae Sedis | 1 | 0.000065 |
| 52 | Caulobacterales | 1 | 0.000065 |
| 53 | Thermomicrobiales | 1 | 0.000065 |
| 54 | Rickettsiales | 1 | 0.000033 |
| 55 | Desulfarculales | 1 | 0.000033 |
| 56 | Gemmatales | 1 | 0.000033 |
| 57 | Acetobacterales | 1 | 0.000033 |
| 58 | Nostocales | 1 | 0.000033 |
| 59 | S085 | 1 | 0.000033 |
| 60 | Flavobacteriales | 2 | 0.000033 |
| 61 | Sphingobacteriales | 1 | 0.000016 |
| 62 | Xanthomonadales | 1 | 0.000016 |
| 63 | Nitrospirales | 1 | 0.000016 |
| 64 | Actinomycetales | 1 | 0.000016 |
| 65 | Izimaplasmatales | 1 | 0.000016 |
| 66 | Cytophagales | 1 | 0.000016 |

## 12.4 Family

**Supplementary Table 63: Relative abundance of microbial families
in Labrador Retriever puppy faecal samples**

| Family Number | Family | Number of samples | Abundance (percentage) |
| --- | --- | --- | --- |
| 1 | Prevotellaceae | 213 | 35.831420 |
| 2 | Fusobacteriaceae | 213 | 16.287925 |
| 3 | Bacteroidaceae | 213 | 14.598273 |
| 4 | Ruminococcaceae | 213 | 7.400819 |
| 5 | Lachnospiraceae | 213 | 6.369275 |
| 6 | Burkholderiaceae | 212 | 4.522662 |
| 7 | Erysipelotrichaceae | 218 | 3.218999 |
| 8 | Peptostreptococcaceae | 212 | 2.181912 |
| 9 | Acidaminococcaceae | 197 | 2.091342 |
| 10 | Veillonellaceae | 208 | 1.804255 |
| 11 | Succinivibrionaceae | 190 | 1.075777 |
| 12 | Unclassified | 197 | 0.868402 |
| 13 | Clostridiaceae 1 | 199 | 0.702456 |
| 14 | Enterobacteriaceae | 158 | 0.528864 |
| 15 | Coriobacteriaceae | 207 | 0.520337 |
| 16 | Streptococcaceae | 120 | 0.354081 |
| 17 | Helicobacteraceae | 193 | 0.294701 |
| 18 | Lactobacillaceae | 56 | 0.246277 |
| 19 | Tannerellaceae | 100 | 0.195196 |
| 20 | Campylobacteraceae | 149 | 0.169875 |
| 21 | Muribaculaceae | 113 | 0.167918 |
| 22 | Anaeroplasmataceae | 95 | 0.142337 |
| 23 | Bifidobacteriaceae | 79 | 0.104380 |
| 24 | Enterococcaceae | 63 | 0.068250 |
| 25 | Peptococcaceae | 136 | 0.058141 |
| 26 | Family XIII | 81 | 0.045554 |
| 27 | Eggerthellaceae | 154 | 0.034011 |
| 28 | Rikenellaceae | 31 | 0.024668 |
| 29 | Mycoplasmataceae | 22 | 0.021995 |
| 30 | Brachyspiraceae | 50 | 0.015489 |
| 31 | Deferribacteraceae | 53 | 0.013957 |
| 32 | Marinifilaceae | 28 | 0.012261 |
| 33 | Christensenellaceae | 33 | 0.004418 |
| 34 | Atopobiaceae | 11 | 0.004011 |
| 35 | Bacillaceae | 7 | 0.003016 |
| 36 | Leuconostocaceae | 2 | 0.002168 |
| 37 | Xanthobacteraceae | 4 | 0.001582 |
| 38 | Corynebacteriaceae | 5 | 0.001207 |
| 39 | Desulfovibrionaceae | 14 | 0.001190 |
| 40 | Paenibacillaceae | 5 | 0.001141 |
| 41 | Planococcaceae | 2 | 0.000978 |
| 42 | Streptomycetaceae | 2 | 0.000717 |
| 43 | Barnesiellaceae | 5 | 0.000652 |
| 44 | Propionibacteriaceae | 2 | 0.000620 |
| 45 | Pseudomonadaceae | 5 | 0.000620 |
| 46 | Micromonosporaceae | 2 | 0.000522 |
| 47 | Nocardioidaceae | 3 | 0.000408 |
| 48 | Sphingomonadaceae | 4 | 0.000391 |
| 49 | Reyranellaceae | 1 | 0.000310 |
| 50 | Pasteurellaceae | 4 | 0.000310 |
| 51 | Paracaedibacteraceae | 4 | 0.000277 |
| 52 | Carnobacteriaceae | 2 | 0.000261 |
| 53 | Saccharimonadaceae | 6 | 0.000245 |
| 54 | Staphylococcaceae | 4 | 0.000196 |
| 55 | 67-14 | 1 | 0.000196 |
| 56 | Microbacteriaceae | 2 | 0.000179 |
| 57 | Akkermansiaceae | 3 | 0.000179 |
| 58 | Rhizobiaceae | 1 | 0.000163 |
| 59 | Chthoniobacteraceae | 1 | 0.000163 |
| 60 | Solirubrobacteraceae | 1 | 0.000147 |
| 61 | Brevinemataceae | 1 | 0.000130 |
| 62 | Moraxellaceae | 3 | 0.000130 |
| 63 | Eubacteriaceae | 3 | 0.000130 |
| 64 | Diplorickettsiaceae | 3 | 0.000130 |
| 65 | Nakamurellaceae | 1 | 0.000114 |
| 66 | Coriobacteriales Incertae Sedis | 3 | 0.000098 |
| 67 | Pseudonocardiaceae | 1 | 0.000098 |
| 68 | Mycobacteriaceae | 1 | 0.000082 |
| 69 | Blastocatellaceae | 1 | 0.000065 |
| 70 | uncultured Chloroflexus sp. | 1 | 0.000065 |
| 71 | uncultured Chloroflexi bacterium | 1 | 0.000065 |
| 72 | Gemmatimonadaceae | 1 | 0.000065 |
| 73 | Spirochaetaceae | 1 | 0.000065 |
| 74 | Caulobacteraceae | 1 | 0.000065 |
| 75 | Nannocystaceae | 1 | 0.000065 |
| 76 | JG30-KF-CM45 | 1 | 0.000065 |
| 77 | Micrococcaceae | 2 | 0.000049 |
| 78 | Desulfarculaceae | 1 | 0.000033 |
| 79 | Gemmataceae | 1 | 0.000033 |
| 80 | Clostridiales vadinBB60 group | 1 | 0.000033 |
| 81 | Acetobacteraceae | 1 | 0.000033 |
| 82 | Porphyromonadaceae | 1 | 0.000033 |
| 83 | Xenococcaceae | 1 | 0.000033 |
| 84 | Devosiaceae | 1 | 0.000033 |
| 85 | BIrii41 | 1 | 0.000033 |
| 86 | Sphingobacteriaceae | 1 | 0.000016 |
| 87 | Alicyclobacillaceae | 1 | 0.000016 |
| 88 | Rhodanobacteraceae | 1 | 0.000016 |
| 89 | SC-I-84 | 1 | 0.000016 |
| 90 | Nitrospiraceae | 1 | 0.000016 |
| 91 | Actinomycetaceae | 1 | 0.000016 |
| 92 | Weeksellaceae | 1 | 0.000016 |
| 93 | Izimaplasmataceae | 1 | 0.000016 |
| 94 | Aerococcaceae | 1 | 0.000016 |
| 95 | Flavobacteriaceae | 1 | 0.000016 |
| 96 | Spirosomaceae | 1 | 0.000016 |

**Supplementary Figure 44: The relative abundance of 30 most
abundant microbial families in individual Labrador Retriever puppy
faecal samples**

**Supplementary Table 64: Relative abundance of 30 most abundant
microbial families in Labrador Retriever puppy faecal samples, by wave
of sample collection**

| Family Number | Family | Wave | Number of samples | Abundance (percentage) |
| --- | --- | --- | --- | --- |
| 1 | Prevotellaceae | Wave 1 | 76 | 36.222069 |
| 1 | Prevotellaceae | Wave 2 | 71 | 35.401393 |
| 1 | Prevotellaceae | Wave 3 | 66 | 35.844186 |
| 2 | Fusobacteriaceae | Wave 1 | 76 | 14.776186 |
| 2 | Fusobacteriaceae | Wave 2 | 71 | 15.904121 |
| 2 | Fusobacteriaceae | Wave 3 | 66 | 18.441596 |
| 3 | Bacteroidaceae | Wave 1 | 76 | 13.953354 |
| 3 | Bacteroidaceae | Wave 2 | 71 | 15.305474 |
| 3 | Bacteroidaceae | Wave 3 | 66 | 14.580130 |
| 4 | Ruminococcaceae | Wave 1 | 76 | 6.261641 |
| 4 | Ruminococcaceae | Wave 2 | 71 | 8.092661 |
| 4 | Ruminococcaceae | Wave 3 | 66 | 7.968345 |
| 5 | Lachnospiraceae | Wave 1 | 76 | 6.869614 |
| 5 | Lachnospiraceae | Wave 2 | 71 | 6.248004 |
| 5 | Lachnospiraceae | Wave 3 | 66 | 5.923587 |
| 6 | Burkholderiaceae | Wave 1 | 75 | 4.914459 |
| 6 | Burkholderiaceae | Wave 2 | 71 | 4.523379 |
| 6 | Burkholderiaceae | Wave 3 | 66 | 4.070730 |
| 7 | Erysipelotrichaceae | Wave 1 | 76 | 3.179371 |
| 7 | Erysipelotrichaceae | Wave 2 | 71 | 3.333032 |
| 7 | Erysipelotrichaceae | Wave 3 | 66 | 3.141960 |
| 8 | Peptostreptococcaceae | Wave 1 | 75 | 2.295080 |
| 8 | Peptostreptococcaceae | Wave 2 | 71 | 2.081543 |
| 8 | Peptostreptococcaceae | Wave 3 | 66 | 2.159571 |
| 9 | Acidaminococcaceae | Wave 1 | 68 | 2.022190 |
| 9 | Acidaminococcaceae | Wave 2 | 65 | 2.192624 |
| 9 | Acidaminococcaceae | Wave 3 | 64 | 2.062016 |
| 10 | Veillonellaceae | Wave 1 | 72 | 1.986365 |
| 10 | Veillonellaceae | Wave 2 | 71 | 1.785619 |
| 10 | Veillonellaceae | Wave 3 | 65 | 1.614601 |
| 11 | Succinivibrionaceae | Wave 1 | 63 | 1.304411 |
| 11 | Succinivibrionaceae | Wave 2 | 67 | 1.132239 |
| 11 | Succinivibrionaceae | Wave 3 | 60 | 0.751761 |
| 12 | Unclassified | Wave 1 | 63 | 1.134197 |
| 12 | Unclassified | Wave 2 | 61 | 0.937565 |
| 12 | Unclassified | Wave 3 | 47 | 0.487932 |
| 13 | Clostridiaceae 1 | Wave 1 | 73 | 0.914815 |
| 13 | Clostridiaceae 1 | Wave 2 | 66 | 0.721076 |
| 13 | Clostridiaceae 1 | Wave 3 | 60 | 0.437892 |
| 14 | Enterobacteriaceae | Wave 1 | 50 | 0.886667 |
| 14 | Enterobacteriaceae | Wave 2 | 58 | 0.357799 |
| 14 | Enterobacteriaceae | Wave 3 | 50 | 0.300873 |
| 15 | Coriobacteriaceae | Wave 1 | 74 | 0.612817 |
| 15 | Coriobacteriaceae | Wave 2 | 69 | 0.478272 |
| 15 | Coriobacteriaceae | Wave 3 | 64 | 0.459097 |
| 16 | Streptococcaceae | Wave 1 | 39 | 0.540253 |
| 16 | Streptococcaceae | Wave 2 | 38 | 0.252783 |
| 16 | Streptococcaceae | Wave 3 | 43 | 0.248675 |
| 17 | Helicobacteraceae | Wave 1 | 62 | 0.435108 |
| 17 | Helicobacteraceae | Wave 2 | 68 | 0.181076 |
| 17 | Helicobacteraceae | Wave 3 | 63 | 0.255253 |
| 18 | Lactobacillaceae | Wave 1 | 23 | 0.359757 |
| 18 | Lactobacillaceae | Wave 2 | 18 | 0.087261 |
| 18 | Lactobacillaceae | Wave 3 | 15 | 0.286666 |
| 19 | Tannerellaceae | Wave 1 | 36 | 0.331609 |
| 19 | Tannerellaceae | Wave 2 | 36 | 0.129620 |
| 19 | Tannerellaceae | Wave 3 | 28 | 0.108657 |
| 20 | Campylobacteraceae | Wave 1 | 54 | 0.171311 |
| 20 | Campylobacteraceae | Wave 2 | 56 | 0.186016 |
| 20 | Campylobacteraceae | Wave 3 | 39 | 0.150857 |
| 21 | Muribaculaceae | Wave 1 | 34 | 0.205079 |
| 21 | Muribaculaceae | Wave 2 | 39 | 0.156864 |
| 21 | Muribaculaceae | Wave 3 | 40 | 0.137019 |
| 22 | Anaeroplasmataceae | Wave 1 | 25 | 0.096645 |
| 22 | Anaeroplasmataceae | Wave 2 | 31 | 0.108196 |
| 22 | Anaeroplasmataceae | Wave 3 | 39 | 0.231680 |
| 23 | Bifidobacteriaceae | Wave 1 | 34 | 0.170306 |
| 23 | Bifidobacteriaceae | Wave 2 | 27 | 0.069310 |
| 23 | Bifidobacteriaceae | Wave 3 | 18 | 0.066194 |
| 24 | Enterococcaceae | Wave 1 | 21 | 0.020974 |
| 24 | Enterococcaceae | Wave 2 | 24 | 0.110103 |
| 24 | Enterococcaceae | Wave 3 | 18 | 0.077665 |
| 25 | Peptococcaceae | Wave 1 | 42 | 0.049122 |
| 25 | Peptococcaceae | Wave 2 | 47 | 0.061484 |
| 25 | Peptococcaceae | Wave 3 | 47 | 0.064931 |
| 26 | Family XIII | Wave 1 | 27 | 0.043684 |
| 26 | Family XIII | Wave 2 | 29 | 0.041821 |
| 26 | Family XIII | Wave 3 | 25 | 0.051724 |
| 27 | Eggerthellaceae | Wave 1 | 59 | 0.038155 |
| 27 | Eggerthellaceae | Wave 2 | 49 | 0.033114 |
| 27 | Eggerthellaceae | Wave 3 | 46 | 0.030203 |
| 28 | Rikenellaceae | Wave 1 | 15 | 0.020106 |
| 28 | Rikenellaceae | Wave 2 | 12 | 0.042701 |
| 28 | Rikenellaceae | Wave 3 | 4 | 0.010524 |
| 29 | Mycoplasmataceae | Wave 1 | 12 | 0.057667 |
| 29 | Mycoplasmataceae | Wave 2 | 5 | 0.001272 |
| 29 | Mycoplasmataceae | Wave 3 | 5 | 0.003210 |
| 30 | Other | Wave 1 | 51 | 0.126987 |
| 30 | Other | Wave 2 | 49 | 0.043582 |
| 30 | Other | Wave 3 | 35 | 0.032466 |

**Supplementary Figure 45: The relative abundance of of 30 most
abundant microbial families by wave of sample collection in Labrador
Retriever puppy faecal samples**

## 12.5 Genus

**Supplementary Table 65: Relative abundance of 30 most abundant
microbial genera in Labrador Retriever puppy faecal samples**

| Genus Number | Genus | Number of samples | Abundance (percentage) |
| --- | --- | --- | --- |
| 1 | Prevotella 9 | 213 | 27.6508 |
| 2 | Fusobacterium | 213 | 16.1072 |
| 3 | Bacteroides | 213 | 14.5983 |
| 4 | Unclassified | 213 | 7.0394 |
| 5 | Alloprevotella | 208 | 6.9746 |
| 6 | Faecalibacterium | 213 | 5.6152 |
| 7 | Sutterella | 209 | 3.8767 |
| 8 | Phascolarctobacterium | 197 | 2.0908 |
| 9 | Blautia | 211 | 1.9939 |
| 10 | Catenibacterium | 184 | 1.7124 |
| 11 | Megamonas | 208 | 1.6091 |
| 12 | Prevotellaceae Ga6A1 group | 113 | 1.1858 |
| 13 | Anaerobiospirillum | 174 | 0.9128 |
| 14 | Parasutterella | 168 | 0.6413 |
| 15 | Collinsella | 207 | 0.5203 |
| 16 | Escherichia-Shigella | 154 | 0.5105 |
| 17 | Holdemanella | 185 | 0.4715 |
| 18 | Fournierella | 199 | 0.4486 |
| 19 | Streptococcus | 119 | 0.3536 |
| 20 | uncultured | 208 | 0.3347 |
| 21 | Helicobacter | 193 | 0.2947 |
| 22 | Turicibacter | 144 | 0.2891 |
| 23 | Lachnospira | 154 | 0.2814 |
| 24 | Negativibacillus | 149 | 0.2770 |
| 25 | Clostridium sensu stricto 1 | 153 | 0.2561 |
| 26 | Lactobacillus | 56 | 0.2463 |
| 27 | Parabacteroides | 100 | 0.1952 |
| 28 | Candidatus Arthromitus | 140 | 0.1939 |
| 29 | Erysipelotrichaceae UCG-003 | 156 | 0.1936 |
| 30 | Butyricicoccus | 212 | 0.1870 |

# 13 Differential abundance analysis

Get the “core” microbiome (taxa are in more than 10% of samples) and
transform to relative abundances

Results for 204 models were produced. The number of models that
converged.fit was 173. The number of models that produced a qval (FDR)
of less than 0.1 was 66.

**Supplementary Figure 46: Differential abundance of amplicon
sequence variants analysed by multivariable association with linear
models (MaAsLin) in Labrador Retriever puppy faecal samples**

**Supplementary Figure 47: Significantly differentially
abundant amplicon sequence variants analysed by multivariable
association with linear models (MaAsLin) in Labrador Retriever puppy
faecal samples**

### 13.0.1 Get the full taxonomy of differentially abundant ASVs

**Supplementary Table 66: Taxonomy table of all significantly
abundant ASVs**

| ASV name | Phylum | Class | Order | Family | Genus |
| --- | --- | --- | --- | --- | --- |
| ASV74.Bacteroides | Bacteroidetes | Bacteroidia | Bacteroidales | Bacteroidaceae | Bacteroides |
| ASV94.Bacteroides | Bacteroidetes | Bacteroidia | Bacteroidales | Bacteroidaceae | Bacteroides |
| ASV55.Bacteroides | Bacteroidetes | Bacteroidia | Bacteroidales | Bacteroidaceae | Bacteroides |
| ASV93.Bacteroides | Bacteroidetes | Bacteroidia | Bacteroidales | Bacteroidaceae | Bacteroides |
| ASV67.Bacteroides | Bacteroidetes | Bacteroidia | Bacteroidales | Bacteroidaceae | Bacteroides |
| ASV62.Alloprevotella | Bacteroidetes | Bacteroidia | Bacteroidales | Prevotellaceae | Alloprevotella |
| ASV66.Alloprevotella | Bacteroidetes | Bacteroidia | Bacteroidales | Prevotellaceae | Alloprevotella |
| ASV87.Prevotella | Bacteroidetes | Bacteroidia | Bacteroidales | Prevotellaceae | Prevotella 9 |
| ASV89.Prevotella | Bacteroidetes | Bacteroidia | Bacteroidales | Prevotellaceae | Prevotella 9 |
| ASV95.Parabacteroides | Bacteroidetes | Bacteroidia | Bacteroidales | Tannerellaceae | Parabacteroides |
| ASV12.Candidatus | Firmicutes | Clostridia | Clostridiales | Clostridiaceae 1 | Candidatus Arthromitus |
| ASV13.Candidatus | Firmicutes | Clostridia | Clostridiales | Clostridiaceae 1 | Candidatus Arthromitus |
| ASV3.Clostridium | Firmicutes | Clostridia | Clostridiales | Clostridiaceae 1 | Clostridium sensu stricto 1 |
| ASV2.Clostridiaceae | Firmicutes | Clostridia | Clostridiales | Clostridiaceae 1 | NA |
| ASV123.Eubacterium | Firmicutes | Clostridia | Clostridiales | Family XIII | Eubacterium nodatum group |
| ASV174.Blautia | Firmicutes | Clostridia | Clostridiales | Lachnospiraceae | Blautia |
| ASV172.Blautia | Firmicutes | Clostridia | Clostridiales | Lachnospiraceae | Blautia |
| ASV165.Cellulosilyticum | Firmicutes | Clostridia | Clostridiales | Lachnospiraceae | Cellulosilyticum |
| ASV181.Dorea | Firmicutes | Clostridia | Clostridiales | Lachnospiraceae | Dorea |
| ASV192.Eubacterium | Firmicutes | Clostridia | Clostridiales | Lachnospiraceae | Eubacterium hallii group |
| ASV169.Tyzzerella | Firmicutes | Clostridia | Clostridiales | Lachnospiraceae | Tyzzerella |
| ASV175.Tyzzerella | Firmicutes | Clostridia | Clostridiales | Lachnospiraceae | Tyzzerella 3 |
| ASV198.Lachnospiraceae | Firmicutes | Clostridia | Clostridiales | Lachnospiraceae | NA |
| ASV200.Lachnospiraceae | Firmicutes | Clostridia | Clostridiales | Lachnospiraceae | NA |
| ASV166.Lachnospiraceae | Firmicutes | Clostridia | Clostridiales | Lachnospiraceae | NA |
| ASV179.Lachnospiraceae | Firmicutes | Clostridia | Clostridiales | Lachnospiraceae | NA |
| ASV178.Lachnospiraceae | Firmicutes | Clostridia | Clostridiales | Lachnospiraceae | NA |
| ASV191.Lachnospiraceae | Firmicutes | Clostridia | Clostridiales | Lachnospiraceae | NA |
| ASV195.Lachnospiraceae | Firmicutes | Clostridia | Clostridiales | Lachnospiraceae | NA |
| ASV185.Lachnospiraceae | Firmicutes | Clostridia | Clostridiales | Lachnospiraceae | NA |
| ASV180.Lachnospiraceae | Firmicutes | Clostridia | Clostridiales | Lachnospiraceae | NA |
| ASV170.Lachnospiraceae | Firmicutes | Clostridia | Clostridiales | Lachnospiraceae | NA |
| ASV125.Peptococcus | Firmicutes | Clostridia | Clostridiales | Peptococcaceae | Peptococcus |
| ASV142.Peptostreptococcaceae | Firmicutes | Clostridia | Clostridiales | Peptostreptococcaceae | NA |
| ASV121.Butyricicoccus | Firmicutes | Clostridia | Clostridiales | Ruminococcaceae | Butyricicoccus |
| ASV120.Butyricicoccus | Firmicutes | Clostridia | Clostridiales | Ruminococcaceae | Butyricicoccus |
| ASV106.Faecalibacterium | Firmicutes | Clostridia | Clostridiales | Ruminococcaceae | Faecalibacterium |
| ASV108.Fournierella | Firmicutes | Clostridia | Clostridiales | Ruminococcaceae | Fournierella |
| ASV32.Ruminococcaceae | Firmicutes | Clostridia | Clostridiales | Ruminococcaceae | Ruminococcaceae UCG005 |
| ASV111.Ruminococcaceae | Firmicutes | Clostridia | Clostridiales | Ruminococcaceae | Ruminococcaceae UCG014 |
| ASV107.Ruminococcaceae | Firmicutes | Clostridia | Clostridiales | Ruminococcaceae | NA |
| ASV140.Ruminococcaceae | Firmicutes | Clostridia | Clostridiales | Ruminococcaceae | NA |
| ASV130.Catenibacterium | Firmicutes | Erysipelotrichia | Erysipelotrichales | Erysipelotrichaceae | Catenibacterium |
| ASV136.Erysipelatoclostridium | Firmicutes | Erysipelotrichia | Erysipelotrichales | Erysipelotrichaceae | Erysipelatoclostridium |
| ASV135.Erysipelatoclostridium | Firmicutes | Erysipelotrichia | Erysipelotrichales | Erysipelotrichaceae | Erysipelatoclostridium |
| ASV133.Erysipelotrichaceae | Firmicutes | Erysipelotrichia | Erysipelotrichales | Erysipelotrichaceae | Erysipelotrichaceae UCG004 |
| ASV124.Erysipelotrichaceae | Firmicutes | Erysipelotrichia | Erysipelotrichales | Erysipelotrichaceae | Erysipelotrichaceae UCG010 |
| ASV137.Holdemanella | Firmicutes | Erysipelotrichia | Erysipelotrichales | Erysipelotrichaceae | Holdemanella |
| ASV129.Turicibacter | Firmicutes | Erysipelotrichia | Erysipelotrichales | Erysipelotrichaceae | Turicibacter |
| ASV154.Megasphaera | Firmicutes | Negativicutes | Selenomonadales | Veillonellaceae | Megasphaera |
| ASV47.Anaerobiospirillum | Proteobacteria | Gammaproteobacteria | Aeromonadales | Succinivibrionaceae | Anaerobiospirillum |
| ASV46.Aeromonadales | Proteobacteria | Gammaproteobacteria | Aeromonadales | Unclassified | NA |
| ASV36.Parasutterella | Proteobacteria | Gammaproteobacteria | Betaproteobacteriales | Burkholderiaceae | Parasutterella |
| ASV37.Parasutterella | Proteobacteria | Gammaproteobacteria | Betaproteobacteriales | Burkholderiaceae | Parasutterella |
| ASV34.Parasutterella | Proteobacteria | Gammaproteobacteria | Betaproteobacteriales | Burkholderiaceae | Parasutterella |
| ASV43.Sutterella | Proteobacteria | Gammaproteobacteria | Betaproteobacteriales | Burkholderiaceae | Sutterella |
| ASV39.Sutterella | Proteobacteria | Gammaproteobacteria | Betaproteobacteriales | Burkholderiaceae | Sutterella |
| ASV40.Sutterella | Proteobacteria | Gammaproteobacteria | Betaproteobacteriales | Burkholderiaceae | Sutterella |
| ASV51.EscherichiaShigella | Proteobacteria | Gammaproteobacteria | Enterobacteriales | Enterobacteriaceae | EscherichiaShigella |
| ASV15.Bifidobacterium | Actinobacteria | Actinobacteria | Bifidobacteriales | Bifidobacteriaceae | Bifidobacterium |
| ASV14.Bifidobacterium | Actinobacteria | Actinobacteria | Bifidobacteriales | Bifidobacteriaceae | Bifidobacterium |
| ASV19.Collinsella | Actinobacteria | Coriobacteriia | Coriobacteriales | Coriobacteriaceae | Collinsella |
| ASV114.Helicobacter | Epsilonbacteraeota | Campylobacteria | Campylobacterales | Helicobacteraceae | Helicobacter |
| ASV115.Helicobacter | Epsilonbacteraeota | Campylobacteria | Campylobacterales | Helicobacteraceae | Helicobacter |
| ASV147.Anaeroplasma | Tenericutes | Mollicutes | Anaeroplasmatales | Anaeroplasmataceae | Anaeroplasma |
| ASV118.Brachyspira | Spirochaetes | Brachyspirae | Brachyspirales | Brachyspiraceae | Brachyspira |

15 ASVs could not be assigned taxonomy at Genus level. 0 ASVs could
not be assigned taxonomy at Family level. 0 ASVs could not be assigned
taxonomy at Order level.
