# Supplementary material for: The gut microbiota of Labrador retriever puppies: a longitudinal cohort study
Source: Anim Microbiome. 2025 Oct 17;7:108. doi: 10.1186/s42523-025-00464-2 (PMC12534972; doi:10.1186/s42523-025-00464-2)
Supplement: Supplementary file 4 — Supplementary Material 4; Additional File 4: Data cleaning and processing for ‘The gut microbiota of Labrador Retriever puppies: a longitudinal cohort study’ [file 42523_2025_464_MOESM4_ESM.docx]

**Additional File 4. Data cleaning and processing for ‘The gut microbiota of Labrador Retriever puppies: a longitudinal cohort study’**

**General information about data cleaning and processing**

Data collected from Dogslife registration (including geographical and demographic information), Dogslife lifestyle and health questionnaires and digestive health questionnaires (DHQs) were combined and cleaned in the context of all available information for each puppy. For example, if the owner answered “Never” to a DHQ question that asked about their puppy’s consumption of a certain food, but in their Dogslife questionnaire they mentioned feeding this item (within the relevant time frame), the item would be given a value of “Frequency not specified” and later assigned a positive value at random. The number of times this occurred for each variable of interest are shown under the headings below.

When questions were not circled in the DHQ, negative responses were assumed. This decision was based on follow up phone-calls with owners to understand why their data was missing, who told us they thought the negative response was inferred when they did not circle any answer. The number of times this occurred for each variable of interest are shown under the headings below. When owners circled more than one Likert score as an answer to a single question, the most frequent category selected was assumed.

Information from the DHQ about dietary changes (Q1) and “titbits” (Q2) were excluded from analysis due to wide variability in the answers, which made it difficult to classify the data into meaningful categories. In this analysis, we used data from Dogslife registration (available for all 76 puppies), 469 Dogslife lifestyle and health questionnaires and 214 DHQs to produce ‘variables of interest’, which were wave of collection, age (descriptive purposes only), sex, coat colour, household type, household smoking status, UK region, area classification, household waste score, outdoors waste score, coprophagia score, stress levels, dog contact score, cat contact score, horse contact score, farm animal contact score, chicken contact score, antibiotics, vomiting and diarrhoea. Details of how these variables were derived are given below. The cleaned data from this file were uploaded under the names “microbiome_study_cleaned_metadata.txt” and “microbiome_study_cleaned_metadata.csv” to the Edinburgh University Datashare and can be publicly accessed at <https://doi.org/10.7488/ds/3857>.

**Wave of collection and age**

*Dogslife registration:*

- *Q: What is your dog's date of birth?*
- *A: [Calendar box]*

*DHQ:*

- *Q: IMPORTANT: What date did you collect your puppy’s faecal and DNA samples?*
- *A: [Free text]*

Cleaning and processing data:

- Date of birth data was not cleaned as it was not suspected any values were incorrect. No date of birth data was missing.
- Date of faecal sample collection was checked for errors by comparison to the dates of sample pack postage to the owner and return from the owner.
- If the date of faecal sample collection was missing, it was estimated as the mid-point between the date the sample pack was sent and was returned.
- Wave of collection was simply the order of DHQ questionnaire that had been returned for that puppy in order of date of the faecal sample collection (e.g., the first questionnaire was wave 1, the 2^nd^ wave 2 etc).
- The age of the puppy at sample was calculated as the faecal sample collection date minus the puppy’s date of birth and divided into days, months and years.

**Sex**

*Dogslife registration:*

- *Q: What sex is your dog?*
- *A: [Female/Male]*

Cleaning and processing data:

- Sex data was not cleaned as it was not suspected any values were incorrect. No sex data was missing.
- No further processing was needed.

**Coat colour**

*Dogslife registration:*

- *Q: What colour is your dog?*
- *A: [Free text]*

Cleaning and processing data:

- Coat colour was categorised as “Black”, “Chocolate”, “Yellow” or “Fox red”. There were 23 puppies that had their colours reclassified from free text. No coat colour data was missing.
- No further processing was needed.

**Household type**

*Dogslife registration:*

- *Q: How would you describe your household?*
- *A: [Single adult/More than one adult/Family (One or more adult and one or more children)/Retired (Single or couple)/Other [Free text]*

Cleaning and processing data:

- Household type was not cleaned as it was not suspected any values were incorrect. No household type data was missing.
- No further processing was needed.

**Household smoking status**

*Dogslife registration:*

- *Q: Does anybody in the household smoke?*
- *A: [Yes/No]*

Cleaning and processing data:

- Smoking status was not cleaned as it was not suspected any values were incorrect. No smoking status data was missing.
- No further processing was needed.

**UK region and area classification**

*Dogslife registration:*

- *Q: Post Code (must be valid UK post code)*
- *A: [Free text]*

Cleaning and processing data:

- Postcodes were checked for validity against UK region and area classification information publicly available from the Office for National Statistics [REF]. Data from the Office for National Statistics is licensed under the Open Government Licence V. 3.0.
- No postcode data was missing, and all postcodes were found to be valid, so the UK region and area classification information were retained for analysis.
- UK region was reclassified into four categories:
  - 28 values from Wales, East Midlands, East of England and West Midlands were reclassified as “England Midlands and Wales”.
  - 13 values from “North East”, “North West” and “Yorkshire and The Humber” were reclassified as “England North”.
  - 23 values from South East and South West were reclassified as “England South.
  - 12 values from Scotland remained in the same category.
- Area class was reclassified into three categories:
  - 28 values containing the words “rural” and/or “farming” were reclassified as “Rural”
  - 18 values containing the words “suburbanites” and/or “suburbia” were reclassified as “Suburban”
  - 30 values containing the words “city dwellers”, “hard-pressed living”, “metropolitans”, “urban” and/or “urbanites” were reclassified as “Urban”

**Household waste score, outdoors waste score and coprophagia**

*DHQ:*

- *Q: How often does your puppy eat other things (not their regular food or treats) such as:*
  - *[Rubbish (e.g. out of a bin)]?*
  - *[Inedible objects (e.g. toys, clothes)]?*
  - *[Grass or Plants/Dead animals (e.g. carcasses on walks)]?*
  - *[Untreated water (e.g. sea, puddles, rivers)]?*
  - *[Other animals’ faeces or poo]?*
  - *[His or her own faeces or poo]?*
- *A: [Never/Occasionally/Weekly/Every Few Days/Daily]*

Cleaning and processing data:

- Data was cross validated against Dogslife questionnaires, when possible, to check for discrepancies in DHQ data, but none were found.
- DHQ Likert scores were converted to numeric values between 0 (“Never”) and 4 (“Daily”) and combined into single variables by using the answer with the highest Likert score across those questions:
  - Household waste score was derived from answers to [Rubbish (e.g. out of a bin)] and [Inedible objects (e.g. toys, clothes)]
  - Outdoors waste score was derived from answers to [Grass or Plants/Dead animals (e.g. carcasses on walks)] and [Untreated water (e.g. sea, puddles, rivers)]
  - Coprophagia score was derived from answers to [Other animals’ faeces or poo] and [His or her own faeces or poo]
- When questions were not circled in the DHQ, negative responses were assumed:
  - In household waste score, 2 missing values were replaced with a zero.
  - In outdoors waste score, 2 missing values were replaced with a zero.
  - In coprophagia score, 4 missing values were replaced with a zero

**Dog contact score, cat contact score, horse contact score, farm animal contact score and chicken contact score**

*Dogslife registration:*

- *Q: Do you have any other pets at home:*
  - *[Dogs]?*
  - *[Cats]?*
  - *[Other]?*
- *A: [Tick box]*
- *Q: EITHER: How many [Dogs/Cats]? OR: Tell us about your other pets?*
- *A: [Free text]*

*DHQ:*

- *Q: How often does your puppy have contact with other animals, such as:*
  - *[Dogs]?*
  - *[Cats]?*
  - *[Horses]?*
  - *[Cows]?*
  - *[Pigs]?*
  - *[Sheep]?*
  - *[Chickens]?*
- *A: [Never/Occasionally/Weekly/Every Few Days/Daily]*

Cleaning and processing data:

- Data was cross validated against Dogslife questionnaires, when possible, to check for discrepancies in DHQ data:
  - In dog contact score, 4 values were changed to “daily”.
  - In cat contact score, 14 values were changed to “daily”.
  - In horse contact score, 0 values were changed.
  - In cow contact score, 0 values were changed.
  - In pig contact score, 0 values were changed.
  - In sheep contact score, 4 values were changed to “daily”.
  - In chicken contact score, 5 values were changed to “daily”.
- DHQ Likert scores were converted to numeric values between 0 (“Never”) and 4 (“Daily”)
- Farm animal contact score was derived by combining answers to [Cows], [Pigs] and [Sheep] into a single variable by using the answer with the highest Likert score across those questions.
- When questions were not circled in the DHQ, negative responses were assumed
  - In dog contact score, 0 values were missing.
  - In cat contact score, 2 missing values were replaced with a zero.
  - In contact with horses score, 4 missing values were replaced with a zero.
  - In contact with farm animals score, 3 values were replaced with a zero.
  - In contact with chickens score, 6 values were replaced with a zero.
- When a positive response was indicated in the owner’s answer, but the frequency was missing, a random positive value with limits and weighted probabilities based on the distribution of the frequency scores of non-missing data was generated:
  - In dog contact score, 0 values were ‘freq not specified’.
  - In cat contact score, 1 value of ‘freq not specified’ was replaced with a random positive value.
  - In horse contact score, 0 values were ‘freq not specified’.
  - In contact with farm animals score, 1 value of ‘freq not specified’ was replaced with a random value.
  - In contact with chickens score, 2 values of ‘freq not specified’ were replaced with a random value.

**Stress levels**

*DHQ:*

- *Q: Which of the following best describes your puppy’s stress levels (please pick one)?*
- *A: [Low (my puppy is seldom stressed)/Medium (my puppy is stressed only in specific situations)/High (my puppy is often stressed)/Very high (my puppy is always stressed)]*

Cleaning and processing data:

- DHQ Likert scores were converted to numeric values between 1 (“Low”) and 3 (“High”).
- Where there were missing values, stress levels were assumed to be the same as the previous data entry. There were 3 missing entries filled in from previous data entries.

**Antibiotics**

*Dogslife lifestyle and health questionnaire:*

- *Q: Has [dog name] had any of the following problems: [Any illness]?*
- *A: [Yes/No]*
- *Q: Did you take [dog name] to your vet for the [Any illness]?*
- *A: [Yes/No]*
- *Q: Approximately when did you visit the vet?*
- *A: [Calendar box]*
- *Q: What is/are the treatment/s?*
- *A: [Free text]*

*DHQ:*

- *Q: As far as you are aware, has your puppy ever been given antibiotics?*
- *A: [Yes/No]*
- *Q: How were they given (e.g. oral tablets, skin creams, injections)?*
- *A: [Free text]*
- *Q: When were they given antibiotics?*
- *A: [Free text]*
- *Q: For how long were they treated with antibiotics?*
- *A: [Free text]*

Cleaning and processing data:

- Treatment with antibiotics was identified in free text within DHQs and Dogslife illness reports with the guidance and expertise of an experienced Veterinarian (Dylan Clements)
- Suspected duplications in illness reports (e.g., when two illnesses with similar descriptions and dates occurred simultaneously) were removed while maximising the information they contained by coalescing where information was missing between the otherwise duplicated entries
- One Dogslife illness report was removed due to the illness occurring after Wave 3 of sample collection
- 36 incidents were identified for antibiotic treatments given by oral or injection methods:
  - 19 were identified in both DHQs and Dogslife illness reports
  - 15 were identified only in DHQs
  - 2 were identified only in Dogslife illness reports
- When illness start, end or veterinary visit dates were suspected to be erroneous (e.g., when the date was before the dog’s date of birth), they were corrected (e.g., when the date was suspected to be a unit error away from the correct date) or removed.
- Dates of antibiotic administration was derived from owner reported information and were carefully cross-checked between DHQs and Dogslife illness reports. If the date that antibiotics were last administered was missing, the date that related to when the illness occurred or was reported to Dogslife was used alternatively.
- The time difference between when the antibiotics were last reported to have been administered orally and the date of faecal sample collection was grouped into time categories: “None”, “Within 1 week”, “1 to 4 weeks”, “4 to 8 weeks”, “8 to 16 weeks” and “Over 16 weeks”. This was based on the distribution of the data and what was deemed to be clinically relevant.

**Vomiting and Diarrhoea**

*Dogslife lifestyle and health questionnaire:*

- *Q: Has [dog name] had any of the following problems:*
  - *[Vomiting]?*
  - *[Diarrhoea]?*
- *A: [Yes/No]*
- *Q: Approximately when did the [Vomiting/Diarrhoea] start?*
- *A: [Calendar box]*
- *Q: Approximately when did the [Vomiting/Diarrhoea] get better?*
- *A: [Calendar box]*
- *Q: Approximately how often did the [Vomiting/Diarrhoea] happen?*
- *A: [Once/Continuous/Every hour/Every 2 hours/Every 6 hour/Every 12 hours/Once a day/Once every 2 days/Once every 3 days/Once weekly/Once every two weeks/Once a month]*
- *Q: Did you take [Dog name] to your vet for the [Vomiting/Diarrhoea]?*
- *A: [Yes/No]*
- *Q: Approximately when did you visit the vet?*
- *A: [Calendar box]*
- *Q: What is/are the treatment/s?*
- *A: [Free text]*

*DHQ:*

- Q: Since you have owned your puppy, has he/she had any signs of an upset stomach, such as:
  - [Vomiting]?
  - *[Diarrhoea]?*
- A: [Yes/No]

Cleaning and processing data:

- Cases of severe vomiting or diarrhoea were defined with the guidance and expertise of an experienced Veterinarian (Dylan Clements) as:
  - In DHQs, if the puppy had been treated with antibiotics (indicates vet treatment) (mentioned in Q6) OR if the puppy visited the vet (mentioned in q10 or q9)
  - In Dogslife illness reports, if the puppy has visited the vet OR if there is more than one occurrence (frequency) of a symptom in a 24-hour period OR if there is one occurrence a day for 3 days or more (duration)
- Suspected duplications in illness reports (e.g., when two illnesses with similar descriptions and dates occurred simultaneously) were removed while maximising the information they contained by coalescing where information was missing between the otherwise duplicated entries.
- 8 Dogslife illness reports were removed due to the illness occurring after Wave 3 of sample collection
- 25 episodes were identified for vomiting:
  - 8 were identified in both DHQs and Dogslife illness reports
  - 6 were identified only in DHQs
  - 11 were identified only in Dogslife illness reports
- 55 episodes were identified for diarrhoea:
  - 18 were identified in both DHQs and Dogslife illness reports
  - 7 were identified only in DHQs
  - 30 were identified only in Dogslife illness reports
- When illness start, end or veterinary visit dates were suspected to be erroneous (e.g., when the date was before the dog’s date of birth), they were corrected (e.g., when the date was suspected to be a unit error away from the correct date) or removed.
- Dates of the vomiting and diarrhoea episode were derived from owner reported information and were carefully cross-checked between DHQs and Dogslife illness reports. If the date the date that the symptom last occurred was missing, the date that the owner recorded the episode to Dogslife was used alternatively.
- The time difference between when a symptom was last reported to have been ongoing and the date of faecal sample collection on the DHQ was grouped into time categories: “Never”, “Within 1 week”, “Within 8 weeks” and “Over 8 weeks”. This was based on the distribution of the data and what was deemed to be clinically relevant.
